# Polyoxymethylene Upcycling into Methanol and Methyl Groups Catalyzed by a Manganese Pincer Complex

**DOI:** 10.1021/jacs.4c07468

**Published:** 2024-07-24

**Authors:** Lijun Lu, Jie Luo, Michael Montag, Yael Diskin-Posner, David Milstein

**Affiliations:** † Department of Molecular Chemistry and Materials Science, Weizmann Institute of Science, Rehovot 7610001, Israel; ‡ Department of Chemical Research Support, Weizmann Institute of Science, Rehovot 7610001, Israel

## Abstract

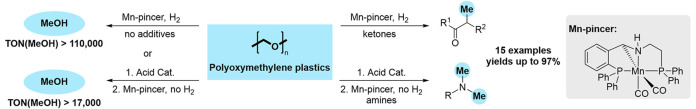

Polyoxymethylene (POM) is a commonly used engineering thermoplastic, but its recycling by conventional means, i.e., mechanical recycling, is not practiced to any meaningful extent, due to technical limitations. Instead, waste POM is typically incinerated or disposed in landfills, where it becomes a persistent environmental pollutant. An attractive alternative to mechanical recycling is upcycling, namely, the conversion of waste POM into value-added chemicals, but this has received very little attention. Herein, we report the upcycling of POM into useful chemicals through three different reactions, all of which are efficiently catalyzed by a single pincer complex of earth-abundant manganese. One method involves hydrogenation of POM into methanol using H_2_ gas as the only reagent, whereas another method converts POM into methanol and CO_2_ through a one-pot process comprising acidolysis followed by Mn-catalyzed disproportionation. The third method utilizes POM as a reagent for the methylation of ketones and amines.

## Introduction

Plastics are an inseparable part of modern human life, being applied in practically every industrial sector, and used for the manufacture of countless consumer products.^1^ However, despite their widely appreciated advantages, plastics are very difficult to degrade and accumulate in the environment as persistent pollutants on a staggering scale, reaching 82 million tonnes (Mt) globally in 2019 alone, according to a recent report.^2^ The environmental impact of plastic production and disposal can be mitigated by recycling, which can reduce our dependence on primary (virgin) plastics and decrease the amount of existing plastic waste. However, the actual rate of recycling is very low, as reflected by the estimate that only 9% of plastic waste was recycled globally in 2019.^2^ One way to overcome this obstacle is to improve and diversify the available recycling processes. At present, almost all recycling is done mechanically, i.e., through grinding and remelting, without chemically altering the primary plastic.^3^ An alternative approach, which is more technologically challenging, and still far less common, is chemical recycling,^4,5^ whereby waste plastic is broken down chemically through various means, such as catalytic depolymerization.

**Figure 1:**
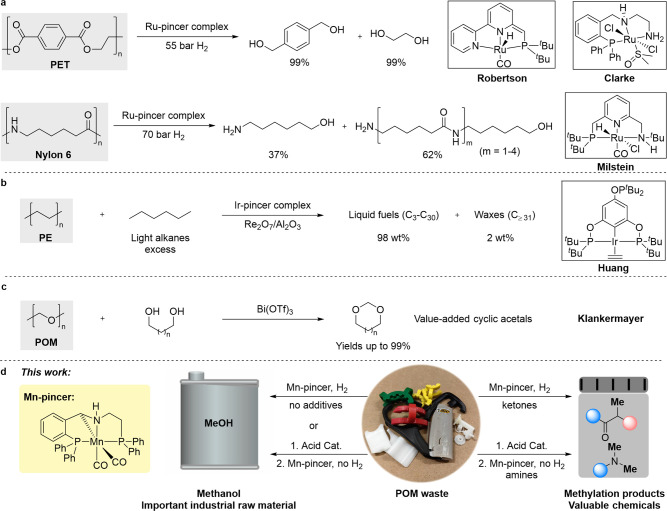
Recycling and upcycling of plastics using metal complexes as catalysts. (a) Examples of plastic depolymerization catalyzed by pincer complexes. (b) Example of plastic upcycling into value-added chemicals catalyzed by a pincer complex. (c) Example of catalytic POM upcycling into value-added chemicals. (d) Mn-catalyzed upcycling of POM into methanol and other chemicals (this work).

Catalytic depolymerization converts waste plastic into monomers or oligomers that can be repolymerized into high-quality primary plastic, and can do so with high selectivity and well-defined products.^6^ As part of the ongoing efforts to develop catalysts for such chemical recycling, several transition metal complexes of pincer ligands have been previously examined by our group,^7,8^ as well as others,^9−12^ and shown to promote the hydrogenolysis-based depolymerization of various plastics, namely, polycarbonates, polyesters, polyamides, and polyureas (see Figure 1a for examples). Pincer-type catalysts have also been instrumental in promoting another kind of chemical plastic recycling, beyond depolymerization, which is known as upcycling.^13^ This involves the conversion of low-cost plastic waste into value-added products, like fuels and fine chemicals, and has been the focus of various catalytic studies.^5,14^ For instance, as shown in Figure 1b, Ir-based pincer complexes have been reported to degrade polyethylene (PE) into liquid fuels and waxes,^15^ and were also involved in the generation of propylene from PE under ethylene gas.^16,17^ Nonpincer catalysts have also been applied for polymer upcycling, such as the conversion of PE and polypropylene (PP) into fatty acids,^18^ or into aldehydes and alcohols.^19^

Polyoxymethylene (POM; Figure 1c) is a relatively common engineering thermoplastic,^20,21^ which is manufactured globally on a multimillion ton scale (e.g., ∼2 Mt in 2020),^22^ and is used primarily in the automotive and electronics industries.^23^ It is also applied in numerous other sectors, and can be found in everyday household items, such as buckles, lighters, and knife handles. Although POM is much less prevalent than commodity plastics like PE, polyethylene terephthalate, or PP,^22^ it poses an environmental concern at both ends of its life cycle. Thus, according to a recent study, the environmental impact of POM manufacturing, in terms of fossil fuel depletion, energy consumption, and climate change potential (CO_2_ equivalents), is higher than that of commodity plastics, when assessed per unit weight of plastic.^24^ Another recent study indicates that POM waste is a more persistent pollutant than commodity plastics, because of its higher density and slower degradation, and is also more hazardous, since it emits toxic formaldehyde (CH_2_O) upon decomposition.^25^ Therefore, POM recycling is imperative for minimizing its effect on the environment. Nevertheless, although POM is recyclable,^20,21^ in practice it is not commercially recycled to any meaningful extent, due to technical limitations, and is instead incinerated or landfilled,^26^ inevitably leading to pollution. This problem is likely to be exacerbated in the future, as POM production is expected to grow markedly in the coming years (i.e., by ∼5% annually, as recently estimated for 2022–2028).^27^

Mechanical recycling of POM, even if it were widely practiced, is hampered by reprocessing-induced degradation,^28^ as is often the case for plastic materials. A favorable alternative is therefore chemical recycling, but this has thus far received relatively little attention. As mentioned above, one approach to such recycling is depolymerization. Previous reports have shown that POM can be converted into CH_2_O and its trimer (1,3,5-trioxane) by treatment with strong inorganic or organic Bro̷nsted acids,^29^ or through a mild process involving *in situ* electrogenerated acid.^30^ Aside from such depolymerization efforts, POM upcycling into useful chemicals has also been explored. Thus, it was recently demonstrated that POM can be converted into hydrogen-rich syngas in supercritical water,^31^ and into liquid petroleum-like mixtures in supercritical toluene.^32^ Catalytic POM upcycling has also been examined, but reports thereof are rare. To the best of our knowledge, only three such systems are currently known, two of which employ Bi(OTf)_3_ as the catalyst – one produces cyclic acetals from POM and diols (Figure 1c),^33^ and the other affords various oxygenates from a mixture of POM and either a diol or carboxylic acid.^34^ The third system involves alumina-catalyzed copyrolysis of POM and lignin to generate biofuel.^35^ These three contributions represent initial efforts to catalytically upcycle POM, and it is clear that much room is left for development. Against this backdrop, we sought to construct a new upcycling system for this plastic, capitalizing on our own experience with pincer-type catalysts that promote a range of hydrogenation and dehydrogenation reactions through metal–ligand cooperation.^36^ Herein, we report the unprecedented one-pot transformation of POM into methanol (MeOH), carried out through two alternative processes – hydrogenation or disproportionation – both catalyzed by the same Mn(I)-pincer complex (Figure 1d). In this manner, waste POM can be turned into a valuable alcohol that is an important industrial raw material.^37^ In addition, we show that POM can be directly employed as a methylating reagent for ketones and amines, applying the same Mn(I) catalyst.

## Results and Discussion

### Catalyst Synthesis

**Figure 2:**
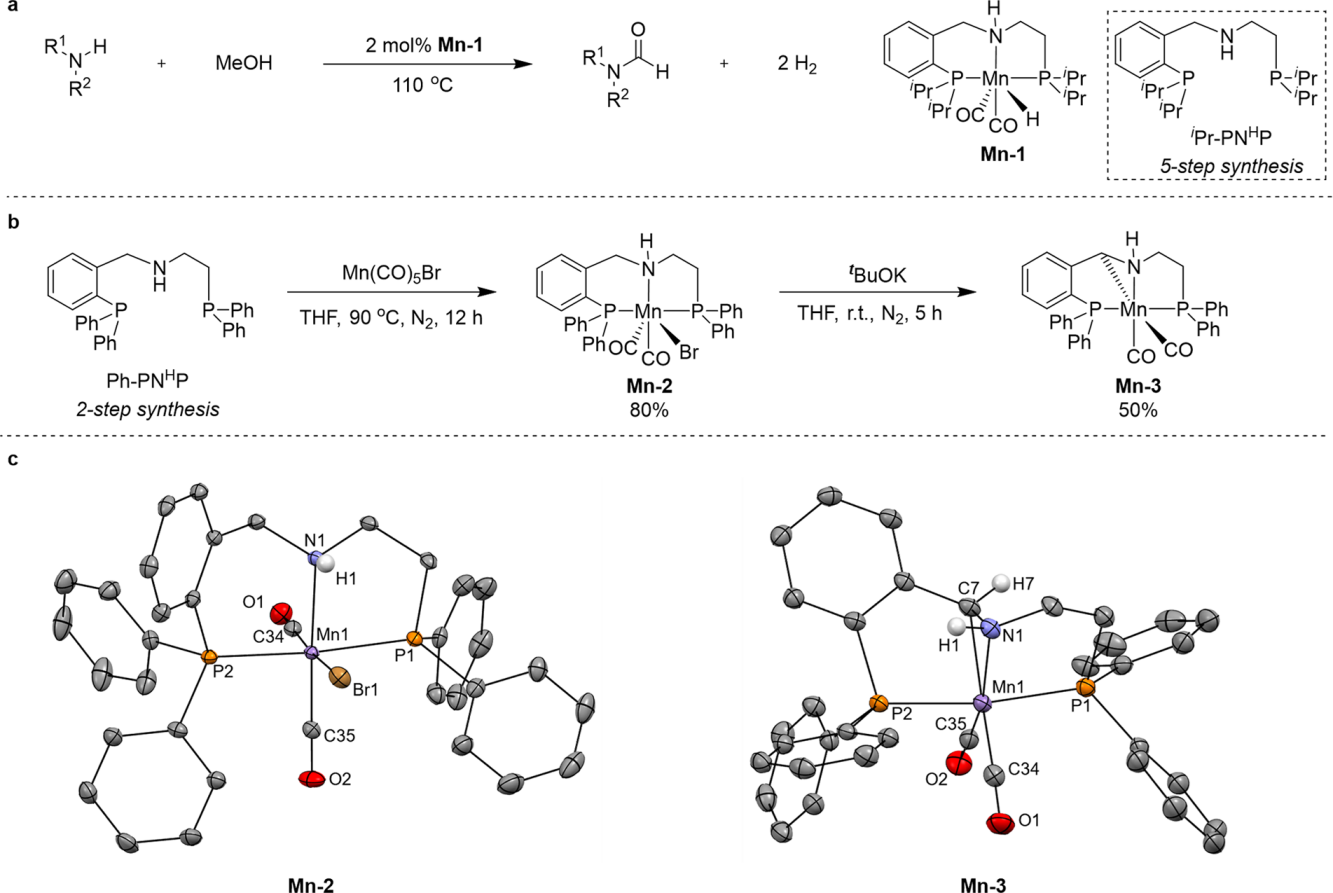
Catalytic Mn(I) complexes of the pincer ligands *^i^*Pr-PN^H^P and Ph-PN^H^P. (a) Previously reported *N*-formylation of amines using methanol as the formylating reagent and **Mn-1** as the catalyst.^39^ (b) Synthesis of **Mn-2** and **Mn-3**. (c) X-ray crystal structures of **Mn-2** and **Mn-3** (thermal ellipsoids are set at the 50% probability level, and most hydrogen atoms were omitted for clarity).

The building block of POM, CH_2_O, as well as its oligomeric variant paraformaldehyde, can be hydrogenated into MeOH using previously described heterogeneous^38^ or homogeneous^9^ catalysts, but no such catalysts have been shown to transform POM itself into MeOH. We have previously demonstrated that the Mn(I)-pincer complex **Mn-1** (Figure 2a) can efficiently catalyze the *N*-formylation of amines using MeOH as the formylating reagent.^39^ Because **Mn-1** can dehydrogenate MeOH into formaldehyde, we reasoned that this type of Mn(I) catalyst could potentially hydrogenate POM – i.e., polyformaldehyde – into MeOH. However, synthesis of the pincer ligand featured in **Mn-1**, namely, *^i^*Pr-PN^H^P, involves a cumbersome 5-step procedure, which could impede its use for practical applications. Therefore, we set to examine Mn-pincer complexes of a phenyl-substituted variant of *^i^*Pr-PN^H^P, the previously reported ligand Ph-PN^H^P (Figure 2b), which is accessible through a simpler two-step synthesis. The precursor to our new catalyst candidate, **Mn-2** (Figures 2b, S1 and S2), was obtained in 80% yield after heating an equimolar mixture of Ph-PN^H^P and Mn(CO)_5_Br in THF at 90 °C (this and subsequently cited temperatures are nominal; see Supporting Information). Treating this precursor with 1 equivalent of *^t^*BuOK resulted in cyclometalation, furnishing the catalyst candidate **Mn-3** (Figures S3–S5), which was isolated in 50% yield. The crystal structures of these pincer complexes are depicted in Figure 2c, and their crystallographic data are provided in Table S1.

### Hydrogenation of POM into MeOH

**Figure 3:**
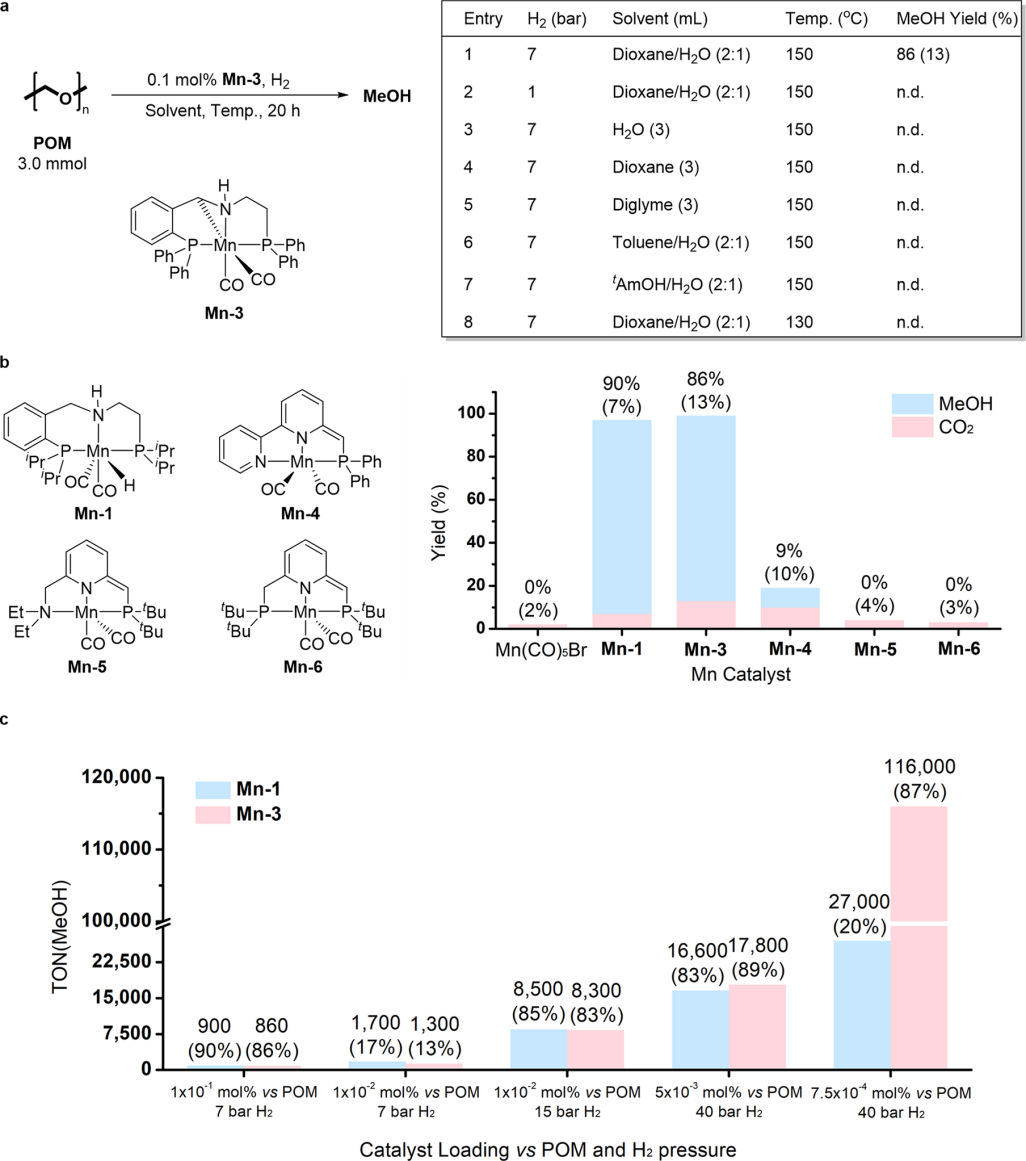
Hydrogenation of POM into MeOH by Mn-pincer complexes. (a) Screening of reaction conditions with **Mn-3** as the catalyst; each reaction was conducted using POM (3.0 mmol) and **Mn-3** (0.1 mol % vs POM), under the catalytic conditions detailed in the table, for 20 h, and the obtained MeOH yield is as noted in the table (CO_2_ yield in parentheses). *^t^*AmOH = *tert*-amyl alcohol. (b) Additional catalysts examined in this work, and a comparison of catalyst activities, based on MeOH yield (CO_2_ yields in parentheses); each reaction involved POM (3.0 mmol) and Mn catalyst (0.1 mol % vs POM), under the conditions noted in table entry 1, for 20 h. (c) Catalytic turnover number (TON) for MeOH generation by complexes **Mn-1** and **Mn-3** at different catalyst loadings (MeOH yields in parentheses); each reaction was conducted using POM (3.0–15.0 mmol) and Mn catalyst (loading as noted in the graph, relative to POM), with catalytic conditions as detailed in table entry 1, but with H_2_ pressure as noted in the graph, for a duration of 20–40 h. Yields of MeOH were determined by ^1^H NMR spectroscopy (dibromomethane was used as the internal standard). Yields of CO_2_ were determined by GC-TCD analysis.

Complex **Mn-3** was investigated as a catalyst for the conversion of POM into MeOH through hydrogenation. The catalytic conditions were explored using commercially available POM as the substrate, in its granular homopolymeric form (Figure 3a). When a sample of this polymer was combined with 0.1 mol % of **Mn-3** in a 2:1 volumetric mixture of dioxane and H_2_O, and the resulting slurry was stirred in a sealed reactor at 150 °C under pressurized H_2_, initially at 7 bar, significant POM-to-MeOH conversion ensued, with the yield of MeOH reaching 86% after 20 h (table entry 1 in Figure 3a). This reaction also generated CO_2_ in 13% yield, likely as a result of CH_2_O disproportionation, which will be addressed below. No MeOH formation was observed when only 1 bar of H_2_ was applied under otherwise identical conditions (entry 2), or when other individual or mixed solvents were used (entries 3–7), or as the temperature was reduced to 130 °C in dioxane/H_2_O (entry 8). Thus, H_2_ pressure, solvent composition, and reaction temperature are all critical factors in the hydrogenation of POM into MeOH.

The catalytic activity of **Mn-3** vis-à-vis POM hydrogenation was compared with that of several other Mn(I) complexes, most of which were developed by our group (Figure 3b). The simple precursor Mn(CO)_5_Br, as well as the complexes **Mn-4**, **Mn-5**, and **Mn-6**, exhibited low to negligible activity in this context. By contrast, **Mn-1** gave results similar to those of **Mn-3**, with MeOH being obtained in 90% yield and CO_2_ in 7% yield. In light of these findings, **Mn-1** and **Mn-3** were chosen as our model catalysts. In an attempt to improve the practicality of our catalytic system and reach higher turnover numbers (TONs), we explored the effect of systematically lowering the catalyst loading. As shown in Figure 3c, reducing catalyst loading from 0.1 to 0.01 mol % sharply decreased the yields of MeOH for both complexes, from ∼90% to under 20%, but the MeOH-based turnover number [TON(MeOH)] increased, from ∼900 to well over 1000 (see Table S2). Raising the H_2_ pressure to 15 bar, with catalyst loading maintained at 0.01 mol %, further increased the TON(MeOH) to above 8000, with MeOH yields rebounding to over 80%. As the loading was halved to 0.005 mol %, while H_2_ pressure was raised to 40 bar, **Mn-3** became somewhat more active than **Mn-1**, with a TON of 17 800 vs 16 600. Remarkably, when catalyst loading was reduced to 7.5 × 10^–4^ mol %, with H_2_ kept at 40 bar, **Mn-3** reached a TON of 116 000 after 40 h, with MeOH being produced in 87% yield and CO_2_ in only 0.2% yield. By contrast, **Mn-1** gave a TON of only ∼27 000, and a MeOH yield of merely 20%, under identical conditions. These results indicate that **Mn-3** is more stable than **Mn-1** under the current catalytic conditions.

### Disproportionation of POM into MeOH and CO_2_

Having achieved the hydrogenation of POM into MeOH with **Mn-3** as a stable and efficient catalyst, we sought to examine whether such upcycling is feasible without externally added H_2_. As mentioned above, POM can be depolymerized into CH_2_O by acidolysis,^29^ prompting us to explore MeOH generation through aldehyde disproportionation rather than hydrogenation. It is known that Cannizzaro self-disproportionation of aqueous formaldehyde, formally expressed as 2CH_2_O + H_2_O → CH_3_OH + HCOOH, can afford MeOH at room temperature under basic conditions.^40^ However, this transformation wastes half of the available CH_2_O, turning it into formic acid. It is possible to extract more MeOH from this system by incorporating the cross-disproportionation of CH_2_O and HCOOH (CH_2_O + HCOOH → CH_3_OH + CO_2_), thereby leading to the overall reaction 3CH_2_O + H_2_O → 2CH_3_OH + CO_2_, but this requires harsh hydrothermal conditions (typically >200 °C at high pressure).^41^ Nonetheless, this reaction can be conducted at much lower temperatures, i.e., 25–80 °C, by using previously reported Ru(II)- or Ir(III)-based catalysts.^42,43^ These were shown to convert aqueous paraformaldehyde into MeOH and CO_2_ in a 2:1 molar ratio, achieving a maximal TON of 493 for Ru and 18 200 for Ir, and an optimal MeOH yield of 93% for both. However, these catalytic systems rely on rare and expensive noble metals, as well as essential additives. To the best of our knowledge, there are no previous reports of catalytic CH_2_O disproportionation into MeOH and CO_2_, which involves non-noble metal catalysts and requires no additives.

**Figure 4:**
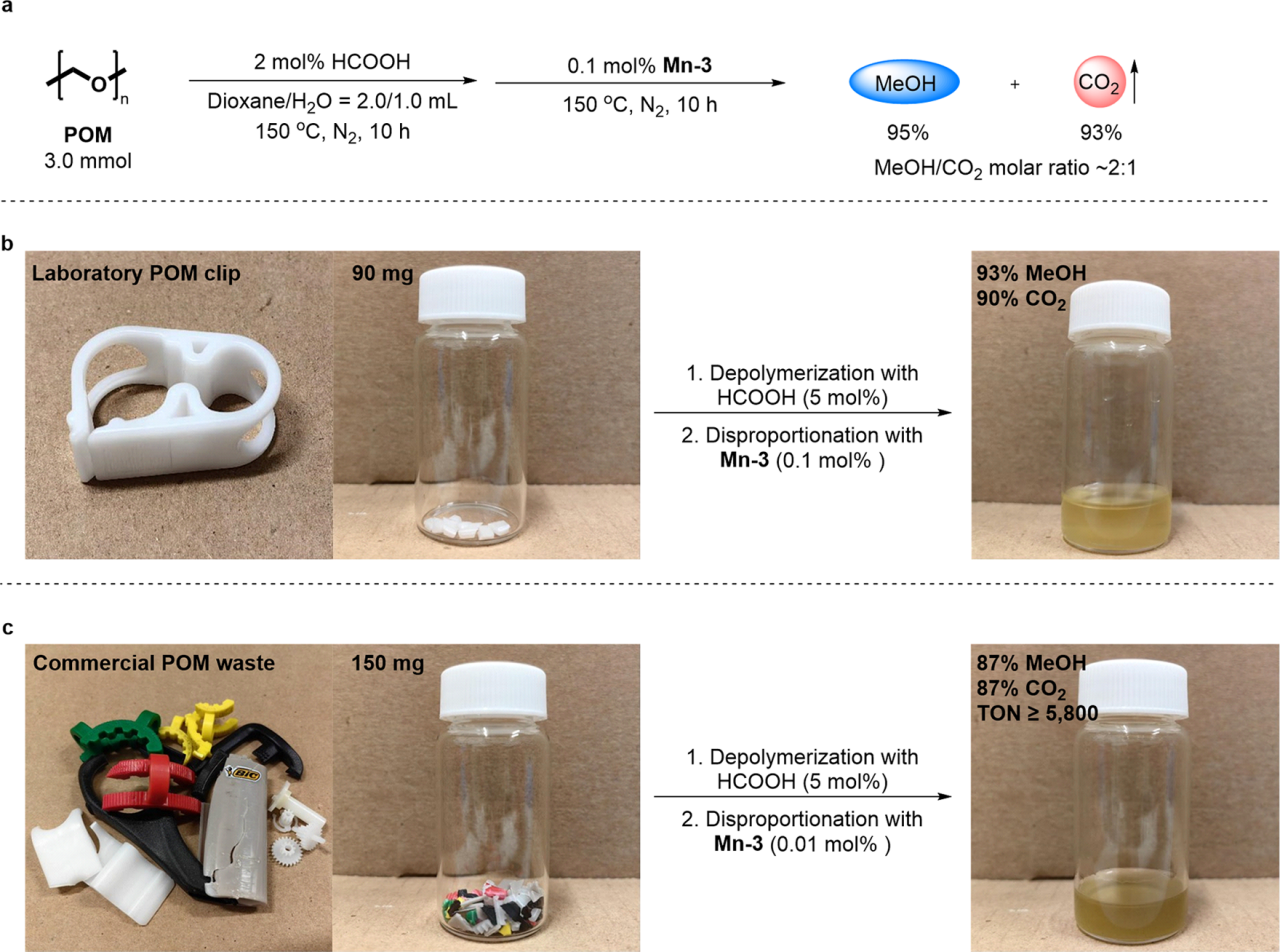
Upcycling of POM into MeOH through Mn-catalyzed disproportionation. (a) One-pot conversion of POM into MeOH and CO_2_ through HCOOH-induced depolymerization followed by disproportionation catalyzed by **Mn-3**. (b,c) Conversion of commercial POM items into MeOH. The reactions outlined in Figure 4a,b were conducted through a two-step procedure: (1) pure POM (3.0 mmol) or POM clip shavings (3.0 mmol), HCOOH (2.0 mol % vs pure POM; 5.0 mol % vs POM clip), dioxane (2.0 mL), and H_2_O (1.0 mL) were mixed and heated at 150 °C for 10 h; (2) **Mn-3** (0.1 mol % vs POM) was added to the reaction mixture, followed by heating at 150 °C for 10 h. The reaction depicted in Figure 4c also involved a two-step procedure: (1) POM waste shavings (5.0 mmol), HCOOH (5.0 mol % vs POM), dioxane (2.0 mL), and H_2_O (1.0 mL) were mixed and heated at 150 °C for 10 h; (2) **Mn-3** (0.01 mol % vs POM) was added to the reaction mixture, followed by heating at 150 °C for 40 h. Yields of MeOH were determined by ^1^H NMR spectroscopy (with dibromomethane as an internal standard). Yields of CO_2_ were determined by GC-TCD analysis, and are based on POM and externally added HCOOH.

After screening various reaction conditions (Tables S3 and S4), we established a simple one-pot two-step procedure for the catalytic disproportionation of homopolymeric POM by **Mn-3**, involving acidolysis followed by Mn-catalyzed disproportionation. In the initial digestion step, a sample of POM is combined with 2 mol % of formic acid in a 2:1 dioxane/H_2_O mixture, and the resulting slurry is stirred at 150 °C for 10 h. Despite being a weak acid, HCOOH has been shown to catalyze POM depolymerization.^44^ Furthermore, we found that **Mn-3** can efficiently dehydrogenate HCOOH into H_2_ and CO_2_, requiring no additives (Figure S6). This allowed us to fully depolymerize POM without having to subsequently neutralize the added acid, since it would be consumed by the Mn catalyst during the disproportionation step. The complete POM-to-MeOH transformation was demonstrated by subjecting a 90 mg sample of POM to HCOOH-induced digestion, and then adding 0.1 mol % of **Mn-3**, followed by 10 h of stirring at 150 °C in a closed vessel. This gave MeOH and CO_2_ in a 2:1 molar ratio and very high yields, namely, 95% and 93%, respectively (Figures 4a and S7).

The practicality of our new POM disproportionation method was demonstrated by applying it to POM consumer products. For such commercially available items, the depolymerization step had to be modified to ensure full conversion, by increasing the amount of HCOOH to 5 mol % (Table S3), probably because these items are not made of homopolymeric POM but consist of a copolymer that is more acid-resistant.^20^ Moreover, this copolymer is usually blended with various additives, such as stabilizers, fillers, and pigments, which may also impede decomposition. An attempt to develop a single-step catalytic process, wherein HCOOH and **Mn-3** are employed simultaneously, was unsuccessful because the rate of HCOOH dehydrogenation by **Mn-3** surpasses that of POM depolymerization by HCOOH (Figure S8). The first POM object to be converted into MeOH was a laboratory clip (Figure 4b). By successively applying the modified acidolysis and Mn-catalyzed disproportionation, we were able to turn 90 mg of clip shavings into MeOH in 93% yield, accompanied by CO_2_ in 90% yield. Subsequently, we applied this catalytic system to an assortment of POM waste objects, namely, various clips, a lighter, a schoolbag buckle, a scissors handle, and toy car gears (Figure 4c). Here, the Mn-catalyzed process was adjusted, using a lower **Mn-3** loading of only 0.01 mol %, but a longer reaction time of 40 h. This afforded both MeOH and CO_2_ in 87% yield, with a TON(MeOH) ≥ 5800. Decreasing the catalyst loading even further, to 0.001 mol %, allowed us to reach a TON(MeOH) ≥ 17 300, albeit with low POM conversion, and a correspondingly low MeOH yield of 26% (Table S5). All in all, we have demonstrated that our catalytic system can disproportionate POM into MeOH and CO_2_ under weakly acidic to neutral conditions using a non-noble metal catalyst, a process that has not been reported previously for either POM or CH_2_O.

### Mechanistic Study

The mechanisms by which **Mn-3** promotes the conversion of POM into MeOH through hydrogenation or disproportionation were explored both experimentally and computationally. To clarify the role of this complex, we conducted several control experiments in its absence. In one of these experiments, a slurry of homopolymeric POM granules in a 2:1 dioxane/H_2_O mixture was heated at 150 °C for 20 h under 7 bar of H_2_. In this case, POM was fully consumed, but the only products observed in solution were CH_2_O and CH_2_(OH)_2_, with no MeOH or HCOOH being detected (Figure S10). CH_2_(OH)_2_ is the product of reversible CH_2_O hydration, which occurs spontaneously in aqueous solutions.^45^ Interestingly, when this experiment was repeated under 7 bar of N_2_ instead of H_2_, no reaction was observed, indicating that dihydrogen is involved in POM depolymerization, although its exact role remains unclear (Figure S11). In two additional experiments, POM granules were combined with either a catalytic (2 mol %) or equivalent amount of HCOOH, and each mixture was heated in dioxane/H_2_O as described above (Figures S12 and S13). Here, too, the polymer fully converted into CH_2_O and CH_2_(OH)_2_, but no MeOH was observed. Taken together, the above results clearly show that self- and cross-disproportionation of CH_2_O do not occur to any significant extent as background reactions during our Mn-catalyzed POM-to-MeOH transformations and that **Mn-3** is directly responsible for MeOH generation. Subjecting POM to the same reaction conditions in the absence of H_2_ or HCOOH, but in the presence of **Mn-3**, resulted in no observable reaction, demonstrating that POM depolymerization is not catalyzed by **Mn-3** (Figure S14) but is essential for MeOH formation. Indeed, when POM was replaced by an aqueous solution of formaldehyde, the latter was efficiently disproportionated by **Mn-3** into MeOH and CO_2_ (Figure S15).

**Figure 5:**
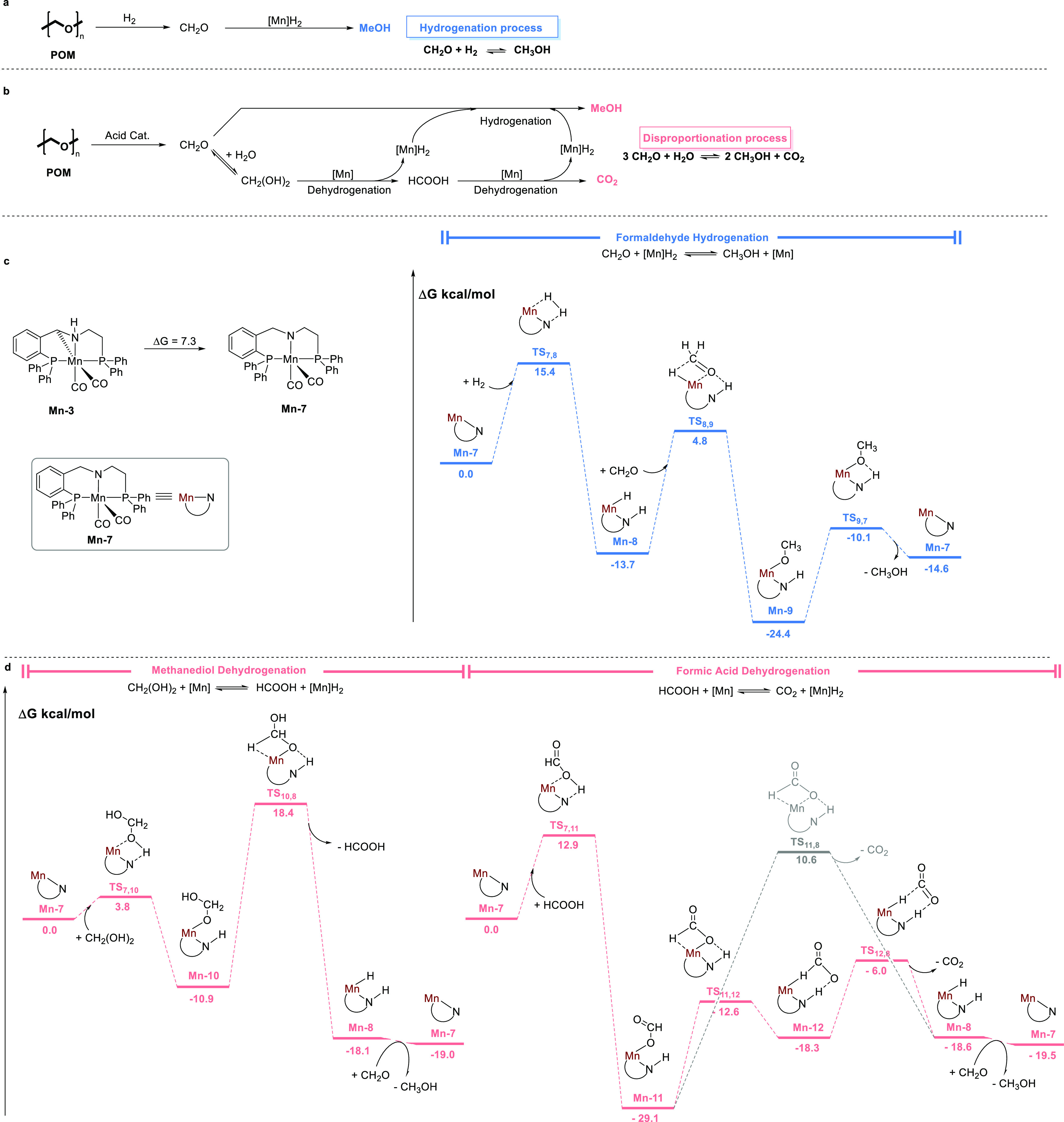
Mechanistic study of the conversion of POM into MeOH catalyzed by **Mn-3**. (a,b) Proposed reaction pathways for POM hydrogenation and disproportionation. (c,d) Computed reaction profiles for the Mn-catalyzed hydrogenation and disproportionation of CH_2_O.

The above observations indicate that **Mn-3** catalyzes hydrogenation and disproportionation reactions involving CH_2_O and CH_2_(OH)_2_, which are the products of POM depolymerization. Based on the known chemistry of Mn-pincer complexes like **Mn-3**,^39^ we propose catalytic mechanisms for these reactions, as outlined in Figure 5a,b. According to these suggested mechanisms, the hydrogenation of CH_2_O into MeOH under an H_2_ atmosphere is mediated by an H_2_ adduct of the catalyst, [Mn]H_2_ (Figure 5a; see below for more details). In the absence of H_2_, wherein CH_2_O disproportionation dominates, the catalyst dehydrogenates CH_2_(OH)_2_ into HCOOH, which is further dehydrogenated into CO_2_. Each hydrogen extrusion reaction generates an equivalent of [Mn]H_2_, which can hydrogenate CH_2_O into MeOH, without requiring an external source of H_2_. These mechanisms were probed by density functional theory (DFT) calculations, using water as an implicit solvent, since the 2:1 dioxane/H_2_O reaction medium is mostly comprised of water, in molar terms [χ(H_2_O) = 0.70]. The computed reaction profiles are shown in Figure 5c,d (also see Table S8).

Our calculations show that the catalytically active species is not **Mn-3**, but rather its isomer **Mn-7** (Figure 5c), which is reversibly generated from the former through 1,2-migration of a proton and is 7.3 kcal/mol less stable, but still energetically accessible. This coordinatively unsaturated species can split an H_2_ molecule across the Mn–N fragment to afford the thermodynamically more stable complex **Mn-8** (Figure 5c, blue pathway), which is the aforementioned H_2_ adduct, [Mn]H_2_. An incoming CH_2_O molecule can then insert into the Mn–H bond of **Mn-8**, thereby forming the Mn–alkoxide species **Mn-9**, which subsequently releases MeOH to regenerate **Mn-7** and close the catalytic cycle for the hydrogenation process. The entire process is thermodynamically downhill (exergonic), with Δ*G*_423.15_ = −14.6 kcal/mol, and its apparent activation energy is 25.2 kcal/mol, represented by the energetic span between **Mn-9** and the transition state **TS**_**7,8**_ from the subsequent catalytic cycle [ΔΔ*G*(**Mn-9**→**TS**_**7,8**_)].^46^

The disproportionation of depolymerized POM is proposed to involve the aforementioned dehydrogenation sequence, CH_2_(OH)_2_ → HCOOH → CO_2_, wherein **Mn-7** extracts an H_2_ equivalent from each hydroxyl-containing molecule (Figure 5d, red pathways). Our computational results indicate that CH_2_(OH)_2_ reacts with **Mn-7** through O–H bond activation across the Mn–N fragment to afford the Mn–alkoxide species **Mn-10**, which then undergoes β-hydride elimination to liberate HCOOH, with concomitant formation of **Mn-8**. Overall, this series of reactions is highly exergonic, with Δ*G*_423.15_ = −19.0 kcal/mol, and its rate-determining step is HCOOH elimination, with ΔΔ*G*^⧧^(**TS**_**10,8**_) = 29.3 kcal/mol. The subsequent dehydrogenation of HCOOH also begins with O–H cleavage by **Mn-7** to give the Mn–formate species **Mn-11**. However, β-hydride elimination from **Mn-11** to generate **Mn-8** and CO_2_ has a prohibitively high kinetic barrier of 39.7 kcal/mol (Figure 5d, gray pathway), which is practically insurmountable even at 150 °C. Nevertheless, as previously demonstrated for other pincer complexes of Mn and Fe,^47,48^**Mn-11** can follow an alternative, stepwise pathway that involves intramolecular rearrangement into **Mn-12** (Figure 5d, red pathway), and exhibits an apparent activation energy of ΔΔ*G*(**Mn-11**→**TS**_**12,8**_) = 23.1 kcal/mol. This represents the overall kinetic barrier for HCOOH dehydrogenation, which is also very exergonic, with Δ*G*_423.15_ = −19.5 kcal/mol. The common product of both dehydrogenation reactions, **Mn-8**, can subsequently hydrogenate CH_2_O into MeOH, or release H_2_ into the reaction medium, regenerating **Mn-7** in either case. Nevertheless, H_2_ liberation is both kinetically and thermodynamically less favorable than CH_2_O hydrogenation (Figure 5c). This was corroborated experimentally when POM disproportionation was conducted in an open system, designed to enhance H_2_ release, but notable amounts of MeOH were still observed (44% yield; Figure S16). All in all, our DFT calculations show that the overall rate-determining process for disproportionation of depolymerized POM is CH_2_(OH)_2_ dehydrogenation, which has a higher kinetic barrier (29.3 kcal/mol) than that of either CH_2_O hydrogenation (25.2 kcal/mol) or HCOOH dehydrogenation (23.1 kcal/mol). These energy barriers are easily overcome under the experimental reaction conditions (i.e., 150 °C).

### POM As a Methylating Reagent for Ketones and Amines

**Figure 6:**
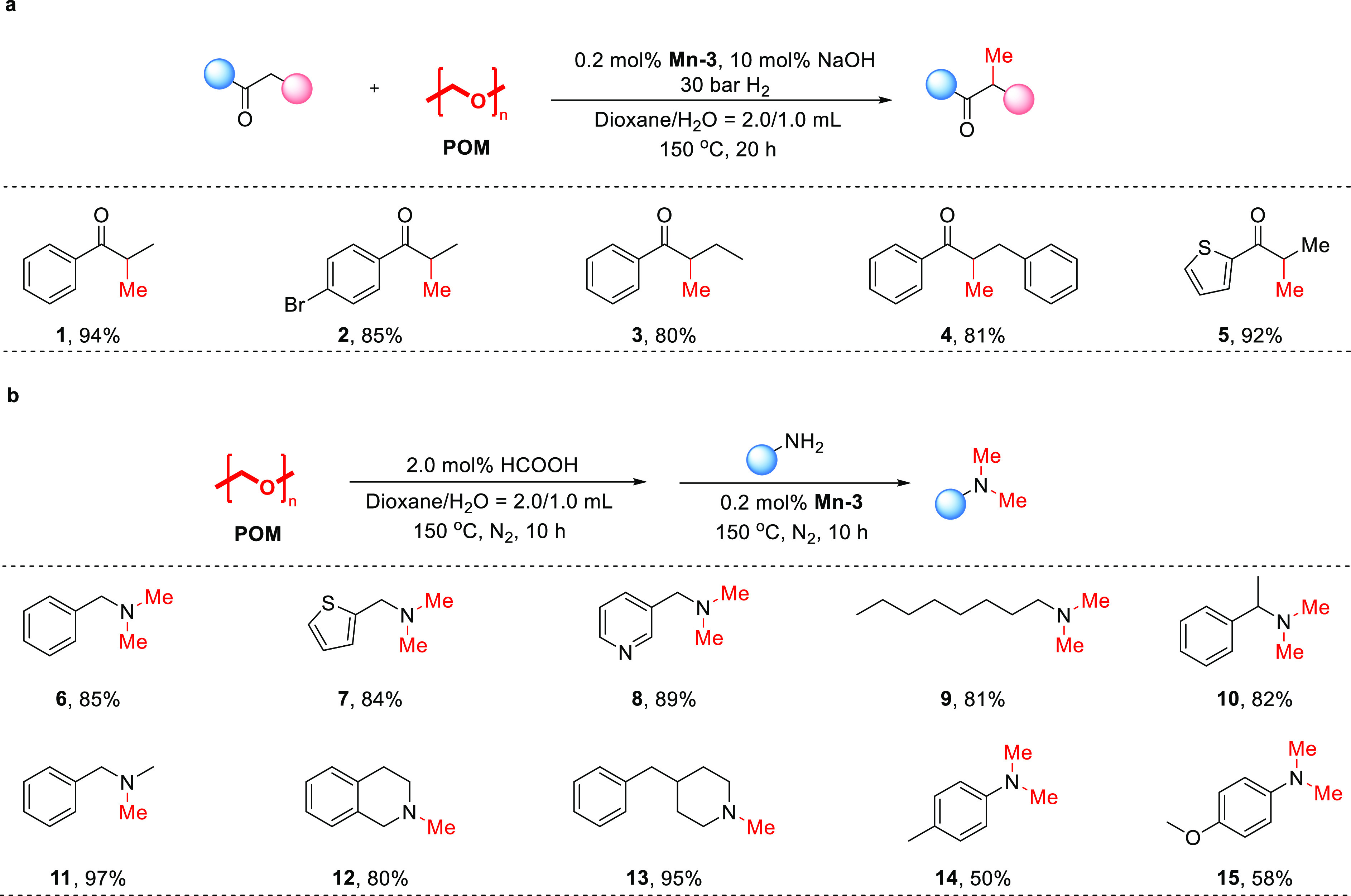
POM as a methylating reagent for ketones and amines. (a) Ketone methylation; each reaction was conducted using POM (1.5 mmol), ketone (0.5 mmol), **Mn-3** (0.2 mol % vs ketone), NaOH (10.0 mol % vs ketone), dioxane (2.0 mL), H_2_O (1.0 mL), and H_2_ (30 bar), and the mixture was heated at 150 °C for 20 h. (b) Amine methylation; each reaction was conducted through a two-step procedure: (1) POM (3.0 mmol for primary amines, 1.5 mmol for secondary amines), HCOOH (2.0 mol % vs POM), dioxane (2.0 mL), and H_2_O (1.0 mL) were mixed and heated at 150 °C for 10 h; (2) amine (0.5 mmol) and **Mn-3** (0.2 mol % vs amine) were added to the reaction mixture, followed by heating at 150 °C for 10 h. The noted yields are those of isolated products.

After demonstrating the ability of our Mn-based catalytic system to upcycle POM into MeOH, we set to examine whether it could facilitate the use of this polymer in the synthesis of other valuable chemicals. Considering the importance of the methyl group in medicinal chemistry,^49^ and that POM is a convenient source of formaldehyde, we explored the possibility of utilizing POM as a methylating reagent. We first applied this polymer for the α-methylation of ketones using **Mn-3** under an H_2_ atmosphere (Figure 6a). After screening various reaction conditions (Table S6), a methylation protocol was established whereby a ketone substrate is combined with 3 equivalent of granulated homopolymeric POM in a 2:1 dioxane/H_2_O mixture, together with 0.2 mol % of **Mn-3** and 10 mol % of NaOH (with loadings relative to the ketone), and the resulting slurry is stirred at 150 °C for 20 h under 30 bar of H_2_. In this manner, several aryl alkyl ketones were converted into the corresponding α-methylated products (**1**–**5**) in high yields (80–94%). An attempt to apply this method for the methylation of a representative amine, benzylamine, was ineffective due to significant side reactions involving the amine, as well as fast POM hydrogenation (Figure S9). This prompted us to attempt amine methylation with our POM disproportionation system (Figure 6b), rather than directly using H_2_ gas as the reductant. By adjusting the reaction conditions (Table S7), we were able to devise an appropriate procedure whereby a sample of granulated POM – 6 equivalent for primary amines and 3 equivalent for secondary ones – was first depolymerized with HCOOH, followed by the addition of the amine substrate, together with **Mn-3** at 0.2 mol % loading (relative to amine), and the mixture was stirred at 150 °C for 10 h in a sealed vessel. Under these conditions, a series of primary and secondary alkylamines were transformed into the respective dimethylated products (**6**−**10**) or monomethylated ones (**11**−**13**) in high yields (80–97%). Anilines were also tolerated under the same catalytic conditions, but the respective products (**14**,**15**) were obtained in only moderate yields (50–58%).

## Conclusions

In summary, we have successfully demonstrated a series of catalytic reactions intended for the upcycling of POM. Instead of simply depolymerizing POM into formaldehyde, we use a Mn-based pincer catalyst to convert this polymer into MeOH and other value-added chemicals. This strategy provides new avenues and opportunities for the upcycling of POM waste, as well as using it as a reagent for catalytic methylation, with the aim of reducing the pollution associated with this plastic material. We are currently exploring similar strategies for the upcycling of other plastic materials.

## Supporting Information Available

Catalyst synthesis, control experiments, experimental procedures, NMR spectra, and computational details (PDF)
