# Supplementary material for: Polyoxymethylene Upcycling into Methanol and Methyl Groups Catalyzed by a Manganese Pincer Complex
Source: J Am Chem Soc. 2024 Jul 24;146(31):22017–26. doi: 10.1021/jacs.4c07468 (PMC11311220; doi:10.1021/jacs.4c07468)
Supplement: Supplementary file 1 — ja4c07468_si_001.pdf [file ja4c07468_si_001.pdf]

Supporting Information for

Polyoxymethylene Upcycling into Methanol and Methyl Groups Catalyzed by a Manganese Pincer Complex

Lijun Lu,[1] Jie Luo,[1] Michael Montag,[1] Yael Diskin-Posner,[2] and David Milstein[1,*]

[1]Department of Molecular Chemistry and Materials Science, Weizmann Institute of Science, Rehovot 7610001, Israel.
[2]Department of Chemical Research Support, Weizmann Institute of Science, Rehovot 7610001, Israel.
*Correspondence to: david.milstein@weizmann.ac.il

Table of Contents:

2 General Considerations
3-6 Catalyst Synthesis
6-7 Single Crystal X-ray Diffraction Analysis of **Mn-2** and **Mn-3**
7-13 General Experimental Procedures
14-15 Control Experiments
15-18 Analytical Data of Products
19-33 NMR Spectra of Products
34-64 Computational Details
64-66 References

1. General Considerations

All reactions investigated in the current work were performed under an atmosphere of purified nitrogen in an MBraun glovebox, or by using standard Schlenk techniques. All commercially available reagents were used as received. All solvents were of HPLC or higher grade, and were degassed with nitrogen and kept in the glovebox over 3Å molecular sieves prior to use. Polyoxymethylene (POM) was obtained from Goodfellow Cambridge Ltd in granular homopolymeric form (3 mm nominal granule size, natural color, injection molding grade). Complexes **Mn-1**,[1] **Mn-4**,[2] **Mn-5**,[3] and **Mn-6**[4] were synthesized according to previously reported procedures. Heating of reaction mixtures was done in a silicone oil bath, and the temperatures cited throughout the manuscript and Supporting Information are all nominal temperatures, i.e., those of the oil bath (the nominal temperature often differs from the actual temperature of the reaction mixture, due to solvent evaporation and reflux). Gas chromatography-mass spectrometry (GC-MS) analysis was carried out on an Agilent Technologies 7820A chromatograph equipped with a 5975 Series Mass Selective Detector, using helium as the carrier gas.

Gas chromatography (GC) analysis was performed on an HP 6890 chromatograph, equipped with a thermal conductivity detector (TCD), using helium as the carrier gas. For each catalytic reaction examined in this work, the conversion and yield were determined through ^{1}H NMR spectroscopy, employing dibromomethane as an internal standard. NMR spectra were recorded using Bruker Avance NEO 300 MHz, Avance NEO 400 MHz, or Avance III HD 500 MHz spectrometers at 293 K. ^{1}H NMR chemical shifts are referenced to the residual hydrogen signal of the deuterated solvent, and the ^{13}C NMR chemical shifts are referenced to the ^{13}C signal of the deuterated solvent. Abbreviations used in the description of NMR data are as follows: br, broad; s, singlet; d, doublet; t, triplet; q, quartet; m, multiplet. Infrared (IR) spectra were recorded on a Thermo Nicolet 6700 FT-IR spectrometer. Analytical TLC was performed on Merck silica gel 60 F254 plates. Flash chromatography columns were packed with 200-300 mesh silica gel.

In the present work, room temperature typically ranges from 22 to 25 °C. Throughout the manuscript and supporting information, all of the cited temperatures are nominal, that is, each of them refers to the temperature of the oil bath used to heat the reaction vessel. The nominal temperature often differs from the actual temperature of the reaction mixture, due to solvent evaporation and reflux. Yields of reactions involving POM were calculated under the assumption that each sample consists of pure homopolymeric POM. Commercial POM items are often composed of a copolymer, and also contain additives, thereby affecting the calculated yield of MeOH, which may be lower than expected.

In aqueous media, CH_2O undergoes reversible hydration into $CH_2(OH)_2$. In the interest of brevity, any mention of CH_2O throughout this document should be understood to implicitly refer to $CH_2(OH)_2$ as well.

Cautionary notes:
(i) When conducting high-pressure experiments with a Fisher-Porter glass tube or steel autoclave, a proper blast shield should be used.
(ii) Hydrogen is a highly flammable gas. Reactions involving H_2 gas should be handled carefully inside a proper fume hood, avoiding open flames, sparks or sources of static electricity.

2. Catalyst Synthesis

Synthesis and characterization of the complex Mn(Ph-PN^{H}P)(CO)$_2$Br (Mn-2)

To a colorless solution of the ligand[5] Ph-PN^{H}P (150.9 mg, 0.3 mmol) in 2.0 mL of THF was added an orange solution of Mn(CO)$_5$Br (82.5 mg, 0.3 mmol) in 4.0 mL of THF, and the resulting mixture was stirred at 90 °C for 12 h, inside a glass pressure tube. The headspace was occasionally placed under reduced pressure to remove free CO. The product precipitated from the solution as a yellow solid. It was then separated by decantation, washed with THF (3×3.0 mL), and placed under reduced pressure to remove residual volatiles. This gave 162.9 mg (0.24 mmol) of the product as a yellow solid (80% yield). ^{1}H NMR (400 MHz, CD_2Cl_2) δ 8.12 (br, 1H, Ar***H***), 7.93-7.90 (m, 2H, Ar***H***), 7.60-7.30 (m, 18H, Ar***H***), 7.15 (t, *J* = 8.0 Hz, 2H, Ar***H***), 6.92 (t, *J* = 8.0 Hz, 1H, Ar***H***), 4.12 (t, *J* = 11.6 Hz, 1H, Ph$_2$PC***H***$_2$C), 4.00-3.97 (m, 1H, ArC***H***$_2$N), 3.76-3.65 (m, 1H, ArC***H***$_2$N), 3.17 (t, *J* = 14.0 Hz, 1H, Ph$_2$PC***H***$_2$C), 3.10-3.04 (m, 1H, NH-C***H***$_2$C), 2.91 (br, 1H, N***H***), 2.34-2.27 (m, 1H, NH-C***H***$_2$C). The ^{1}H NMR peaks associated with impurities in CD_2Cl_2, which can be seen in Figure S1, appear to be enhanced due to the relatively low concentration of the poorly soluble **Mn-2**. ^{31}P{^{1}H} NMR (162 MHz, CD_2Cl_2) δ 73.32 (d, $^2J_{PP}$ = 95.6 Hz), 54.57 (d, $^2J_{PP}$ = 96.6 Hz). ^{13}C NMR data were not measured due to the poor solubility of **Mn-2**. IR (thin film, cm^{-1}) = 1835 (s, ν_{CO}), 1917 (s, ν_{CO}).

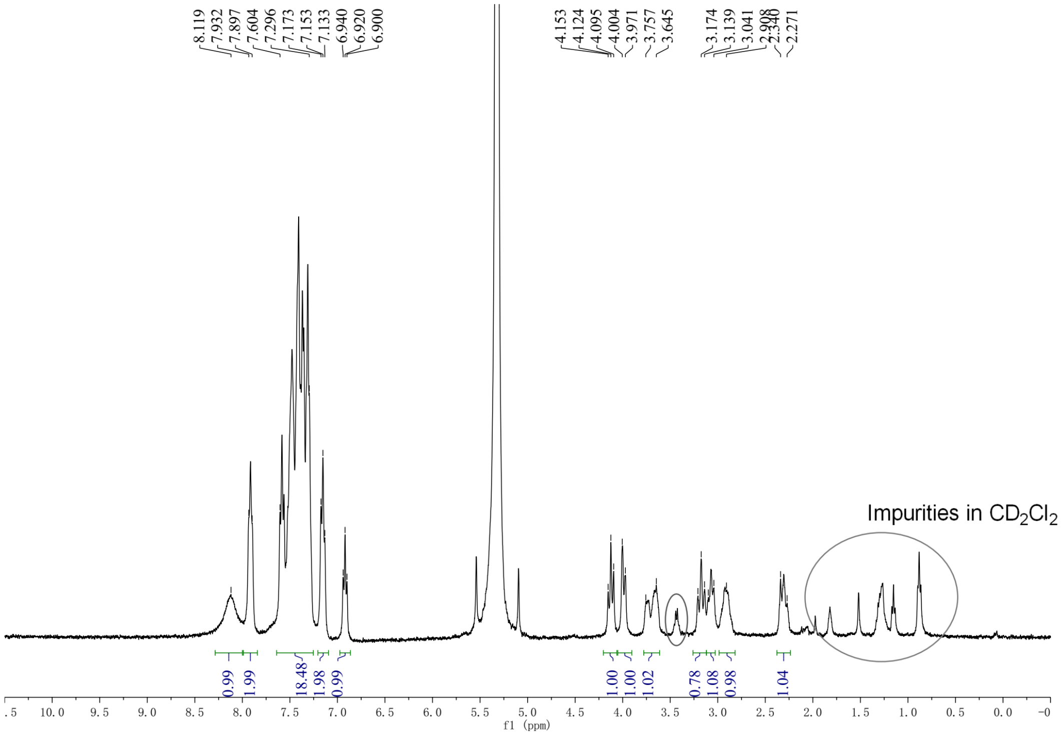

Figure S1. ^{1}H NMR spectrum (400 MHz) of **Mn-2** in CD_2Cl_2.

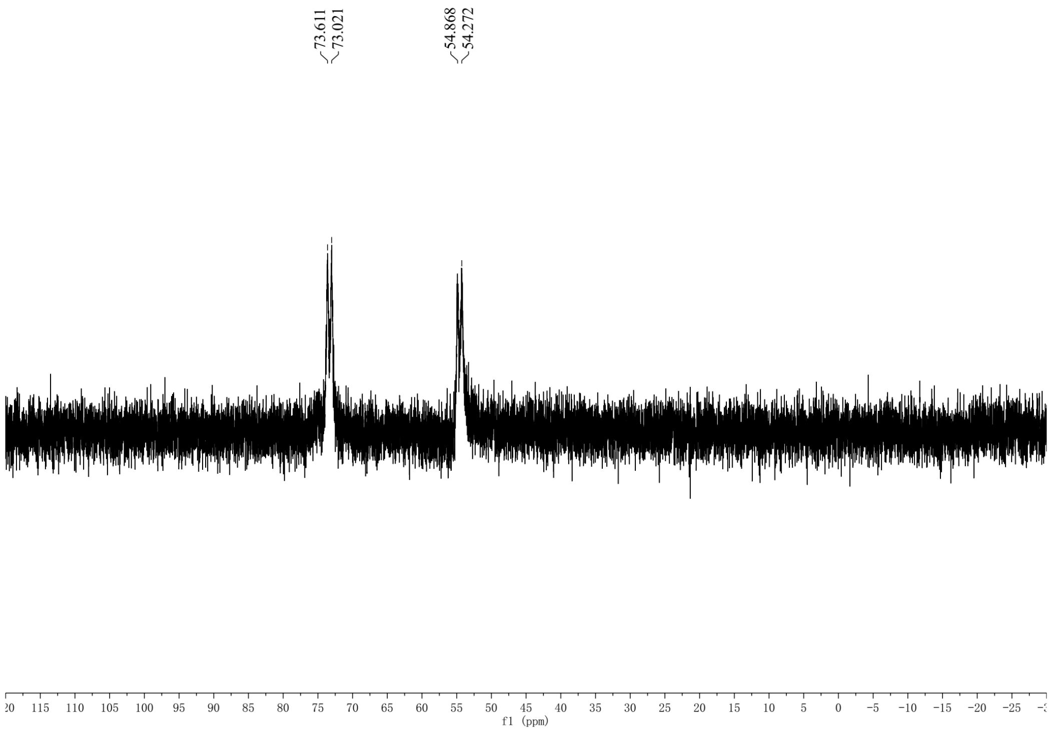

Figure S2. $^{31}P\{^1H\}$ NMR spectrum (162 MHz) of **Mn-2** in CD_2Cl_2.

Synthesis and characterization of the complex Mn(Ph-PN^{H}P)(CO)$_2$ (Mn-3)

Complex **Mn-2** (34.7 mg, 0.05 mmol), ^{t}BuOK (6.7 mg, 0.06 mmol) and 5.0 mL of THF were loaded into a glass vial, and the resulting solution was stirred at room temperature for 5 h, during which its color slowly changed to deep red. The resulting mixture was then passed through a 0.2 μm PTFE filter, and the filtrate was placed under reduced pressure to remove the solvent. The afforded yellowish-red residue was extracted with Et_2O (20.0 mL), and the extract was passed through a 0.2 μm PTFE filter and stored at -40 °C for several days to induce crystallization. The obtained crystals were then separated by decantation, giving 15.3 mg (0.025 mmol) of the product as yellow crystals (50% yield). ^1H NMR (500 MHz, C_6D_6) δ 7.99-7.92 (m, 3H, Ar***H***), 7.60-7.56 (m, 3H, Ar***H***), 7.47 (t, J = 6.5 Hz, 1H, Ar***H***), 7.21-6.93 (m, 17H, Ar***H***, overlaps with solvent signal), 2.54-2.40 (m, 2H, $Ph_2PC\boldsymbol{H}_2C$), 2.28-2.24 (m, 1H, ArC***H***N), 1.86-1.79 (m, 1H, NH-C$\boldsymbol{H}_2$C), 1.73 (br, 1H, N***H***), 1.52-1.45 (m, 1H, NH-C$\boldsymbol{H}_2$C). $^{31}P\{^1H\}$ NMR (203 MHz, C_6D_6) δ 97.0 (d, $^2J_{PP}$ = 79.2 Hz), 91.8 (d, $^2J_{PP}$ = 80.4 Hz). $^{13}C\{^1H\}$ NMR (126 MHz, C_6D_6) δ = 159.31 (J_{cp} = 39.4 Hz, Ar), 139.15 (J_{cp} = 41.2 Hz, Ar), 138.31 (J_{cp} = 45.2 Hz, Ar), 137.20 (J_{cp} = 43.0 Hz, Ar), 136.97 (J_{cp} = 47.1 Hz, Ar), 135.82 (J_{cp} = 27.2 Hz, Ar), 133.66 (J_{cp} = 11.2 Hz, Ar), 133.30 (J_{cp} = 10.6 Hz, Ar), 131.81 (Ar), 131.61 (J_{cp} = 10.7 Hz, Ar), 130.86 (J_{cp} = 10.3 Hz, Ar), 129.56 (Ar), 129.37 (Ar), 128.70 (J_{cp} = 8.8 Hz, Ar), 128.48 (Ar), 128.37 (Ar), 128.26 (J_{cp} = 8.9 Hz, Ar), 128.15 (Ar), 127.98-127.35 (overlapping peaks), 125.85 (J_{cp} = 3.3 Hz, Ar), 59.30 (m, Ar***C***HN), 50.10 (m, NH-***C***H_2C), 26.24 (d, J_{cp} = 12.0 Hz, $Ph_2P\boldsymbol{C}H_2C$). IR (thin film, cm^{-1}) = 1819 (s, ν_{CO}), 1894 (s, ν_{CO}).

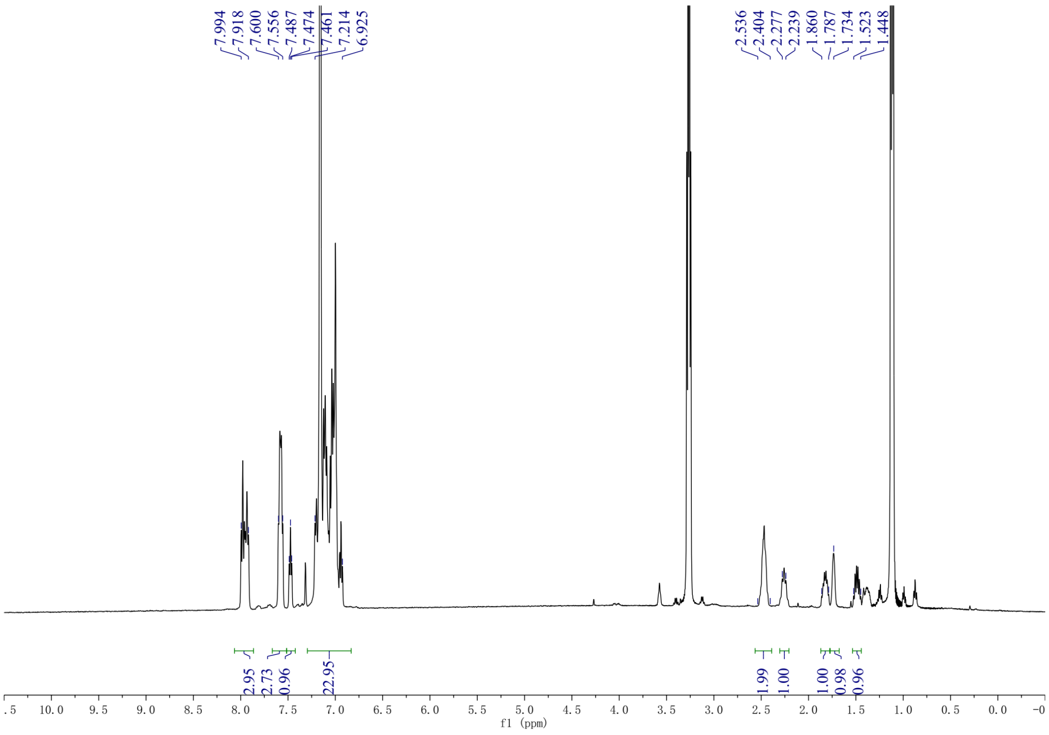

Figure S3. ^{1}H NMR spectrum (500 MHz) of **Mn-3** in C_6D_6.

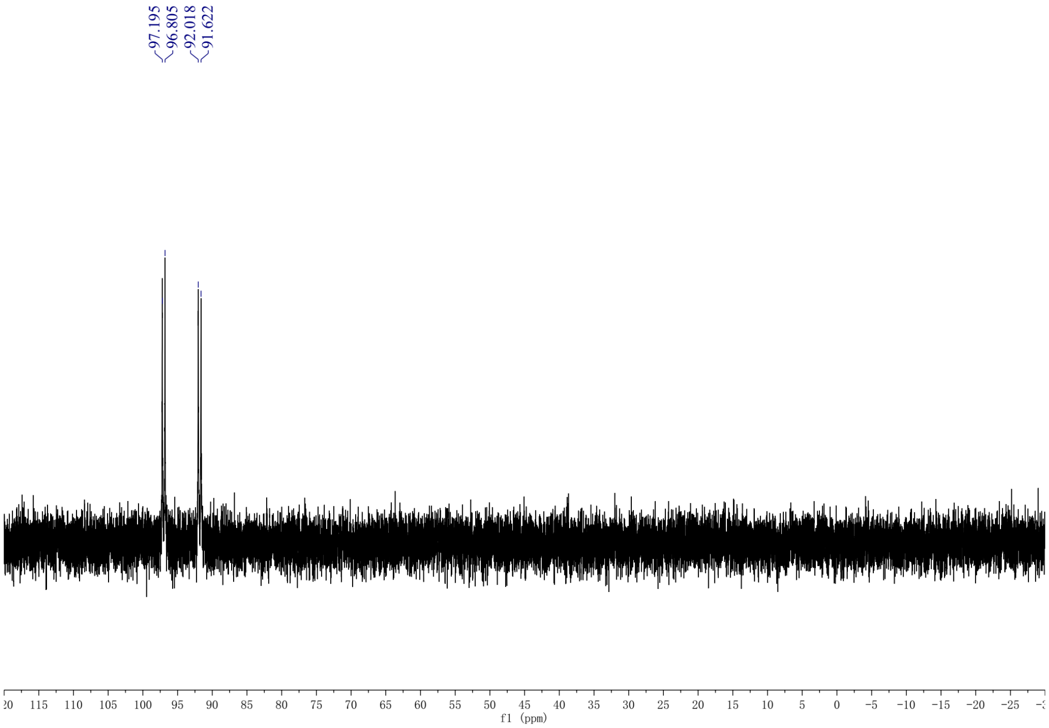

Figure S4. $^{31}P\{^{1}H\}$ NMR spectrum (203 MHz) of **Mn-3** in C_6D_6.

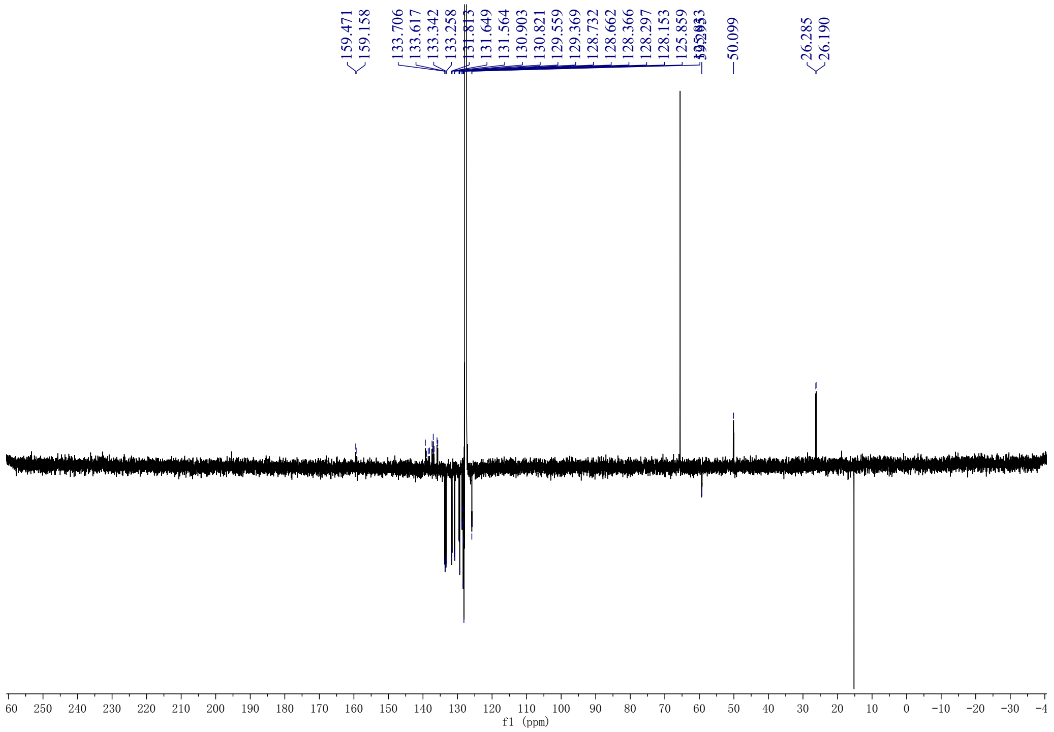

Figure S5. $^{13}C\{^{1}H\}$ DEPTQ NMR spectrum (126 MHz) of **Mn-3** in C_6D_6.

3. Single Crystal X-ray Diffraction Analysis of Mn-2 and Mn-3

Diffraction data from a single crystal of complex **Mn-2** were collected on a Rigaku Synergy-S dual source diffractometer equipped with a hybrid pixel CdTe Dectris 3R 300K detector, using MoKα radiation (λ=0.71073 Å). Diffraction data from a single crystal of complex **Mn-3** were collected on a Rigaku Synergy-R diffractometer equipped with a HyPix-Arc 150° detector, using CuKα radiation (λ=1.54184 Å). The data were processed with CrysAlis^{PRO}.[6] The structures were solved with SHELXT,[7] and refined using SHELXL,[8] with full matrix least-squares based on F^2. All structure solution and refinement programs were implemented in the Olex-2 GUI.[9] All non-hydrogen atoms were refined with anisotropic displacement coefficients, whereas hydrogen atoms were placed in calculated positions and refined in a riding mode.

Table S1. Crystal data and structure refinement parameters for **Mn-2** and **Mn-3**.

Complex	**Mn-2**	**Mn-3**
CCDC	2269139	2269140
Empirical formula	$C_{34.95}H_{31}Br_{1.06}MnNO_{1.95}P_2$	$C_{35}H_{30}MnNO_3P_2$•$C_4H_{10}O$
Crystal description	Orange prism	Yellow needle
Crystal size (mm^3)	0.327 × 0.147 × 0.085	0.250 × 0.050 × 0.030
Formula weight (g/mol)	697.25	687.60
T (K)	100.0(2)	100.0(2)
Wavelength (Å)	0.71073	1.54184
Crystal system	Monoclinic	Monoclinic

Space group	*P* $2_1/n$	*P* $2_1/c$
a (Å)	11.8006(4)	12.8862(2)
b (Å)	20.7537(7)	17.6499(2)
c (Å)	12.6665(4)	15.1444(2)
α (°)	90	90
β (°)	102.458(3)	104.8280(10)
γ (°)	90	90
Volume (Å^3)	3029.06(18)	3329.74(8)
Z	4	4
ρ_{cal} (mg/m^3)	1.529	1.372
μ (mm^{-1})	1.967	4.442
Reflections collected (unique)	49901 (9235)	47008 (6741)
R_{int}	0.0539	0.0422
Completeness to θ (%)	100.0	99.7
Limiting indices	$-16 \le h \le 16$,<br>$-29 \le k \le 29$,<br>$-18 \le l \le 18$	$-16 \le h \le 15$<br>$-21 \le k \le 22$,<br>$-18 \le l \le 14$
Data / restraints / parameters	9235 / 0 / 392	6741 / 0 / 417
Goodness-of-fit on F^2	1.068	1.059
Final R_1 and wR_2 indices [I>2σ(I)]	R_1 = 0.0438, wR_2 = 0.1179	R_1 = 0.0383, wR_2 = 0.0994
R_1 and wR_2 indices (all data)	R_1 = 0.0551, wR_2 = 0.1226	R_1 = 0.0472, wR_2 = 0.1047
Largest diff. peak and hole (e/Å^3)	1.510 and -1.471	0.989 and -0.494

4. General Experimental Procedures

Conversion of POM into MeOH using Mn-3 and H_2 (7 bar)

In the glovebox, a 90 mL Fisher-Porter glass pressure tube was charged with granulated POM (90.0 mg, 3.0 mmol), **Mn-3** (1.8 mg, 0.003 mmol, 0.1 mol% *vs* POM), 1,4-dioxane (2.0 mL), H_2O (1.0 mL) and a Teflon-coated magnetic stirring bar. The tube was then taken out of the glovebox and pressurized with 7 bar of H_2, after which the resulting mixture was stirred at 150 °C for 20 h. Subsequently, the mixture was allowed to cool to room temperature, and the headspace composition was determined by GC-TCD analysis. The excess gas was then vented off, and the liquid reaction mixture was analyzed by ^{1}H and ^{13}C NMR spectroscopy.

Conversion of POM into MeOH using Mn-3 and H_2 (40 bar)

In the glovebox, a 30 mL stainless steel autoclave equipped with a Teflon tube insert was charged with granulated POM (300.0 mg, 10.0 mmol), **Mn-3** (75 μL of 0.001 M solution in dioxane, 7.5×10^{-5} mmol, 7.5×10^{-4} mol% *vs* POM), 1,4-dioxane (3.0 mL), H_2O (1.5 mL) and a Teflon-coated magnetic stirring bar. The autoclave was then removed from the glovebox and pressurized with 40 bar of H_2, after which the resulting mixture was stirred at 150 °C for 40 h. Subsequently, the mixture was allowed to cool to room temperature, and the headspace

composition was determined by GC-TCD analysis. The excess gas was then vented off, and the liquid reaction mixture was analyzed by ^{1}H and ^{13}C NMR spectroscopy.

Table S2. Exploration of reaction conditions for the conversion of POM into MeOH under H_2 using **Mn-1** and **Mn-3** as catalysts.

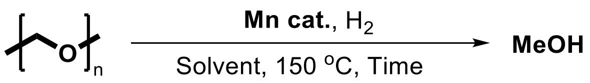

Entry	POM (mmol)	Mn cat. (mol%)	Dioxane/ H_2O (mL)	H_2 pressure (bar)	Reactor	Time (h)	MeOH yield (%) /TON	CO_2 yield (%)
1	3.0	**Mn-1** (0.1)	2.0/1.0	7	Fisher-Porter	20	90/900	7.3
2	3.0	**Mn-3** (0.1)	2.0/1.0	7	Fisher-Porter	20	86/860	13.1
3	10.0	**Mn-1** (0.01)	3.0/1.5	7	Fisher-Porter	20	17/1700	2.0
4	10.0	**Mn-3** (0.01)	3.0/1.5	7	Fisher-Porter	20	13/1300	4.7
5	5.0	**Mn-1** (0.01)	2.0/1.0	15	Autoclave	40	85/8500	2.0
6	5.0	**Mn-3** (0.01)	2.0/1.0	15	Autoclave	40	83/8300	2.1
7	15.0	**Mn-1** (5×10^{-3})	3.0/1.5	40	Autoclave	40	83/16600	0.05
8	15.0	**Mn-3** (5×10^{-3})	3.0/1.5	40	Autoclave	40	89/17800	0.4
9	10.0	**Mn-1** (7.5×10^{-4})	3.0/1.5	40	Autoclave	40	20/26667	0.1
10	10.0	**Mn-3** (7.5×10^{-4})	3.0/1.5	40	Autoclave	40	87/116000	0.2

MeOH yields were determined by ^{1}H NMR spectroscopy (dibromomethane was used as internal standard). CO_2 yields were determined by GC-TCD analysis.

Table S3. Exploration of reaction conditions for POM acidolysis.

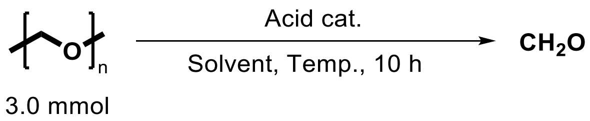

Entry	POM	Solvents (mL)	Acid cat. (mol%)	Temp. (°C)	CH_2O yield (%)
1	POM	Dioxane (3)	HCOOH (5)	150	n.d.
2	POM	Dioxane/H_2O (2/1)	HCOOH (5)	150	> 99
3	POM	Dioxane/H_2O (2/1)	HCOOH (2)	150	> 99

4	POM	Dioxane/H_2O (2/1)	HCOOH (1)	150	~ 95
5	POM	Dioxane/H_2O (2/1)	HCOOH (2)	120	n.d.
6	POM clip	Dioxane/H_2O (2/1)	HCOOH (5)	150	> 99
7	POM clip	Dioxane/H_2O (2/1)	HCOOH (2)	150	~ 70

The resulting solutions were analyzed by ^{1}H and ^{13}C NMR spectroscopy.

Conversion of HCOOH into H_2 and CO_2 using Mn-3

In the glovebox, a 50 mL glass pressure tube was charged with HCOOH (38 µL, 1.0 mmol), **Mn-3** (0.6 mg, 0.001 mmol, 0.1 mol% *vs* HCOOH), 1,4-dioxane (1.0 mL), H_2O (0.5 mL) and a Teflon-coated magnetic stirring bar. The tube was sealed and removed from the glovebox, and the reaction mixture was stirred at 150 °C for 8 h. Subsequently, the mixture was allowed to cool to room temperature, and the headspace composition was determined by GC-TCD analysis.

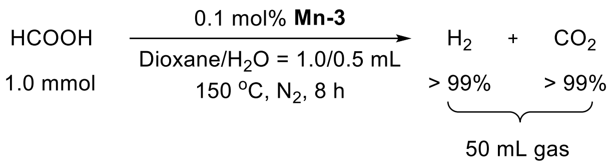

Figure S6. Dehydrogenation of HCOOH catalyzed by **Mn-3**.

Conversion of POM into MeOH using Mn-3 and HCOOH

In the glovebox, a 50 mL glass pressure tube, was charged with granulated POM (90.0 mg, 3.0 mmol), HCOOH (2.3 µL, 0.06 mmol, 2 mol% *vs* POM), 1,4-dioxane (2.0 mL), H_2O (1.0 mL) and a Teflon-coated magnetic stirring bar. The tube was sealed and removed from the glovebox, and the reaction mixture was stirred at 150 °C for 10 h, during which all solids were consumed. The tube was then allowed to cool to room temperature and was reintroduced into the glovebox, and **Mn-3** (1.8 mg, 0.003 mmol, 0.1 mol% *vs* POM) was added to the reaction mixture. The tube was resealed and taken out of the glovebox, and the reaction mixture was stirred at 150 °C for 10 h. The mixture was then allowed to cool to room temperature and the headspace composition was determined by GC-TCD analysis. The excess gas was then vented off, and the liquid reaction mixture was analyzed by ^{1}H and ^{13}C NMR spectroscopy.

Table S4. Exploration of reaction conditions for POM disproportionation by **Mn-3**.

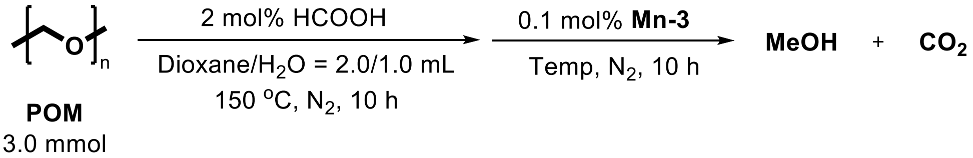

Entry	POM	Temp. (°C)	MeOH yield (%)
1	POM	150	95
2	POM	120	63
3	POM	100	37

Yields were determined by ^{1}H NMR spectroscopy (dibromomethane was used as internal standard).

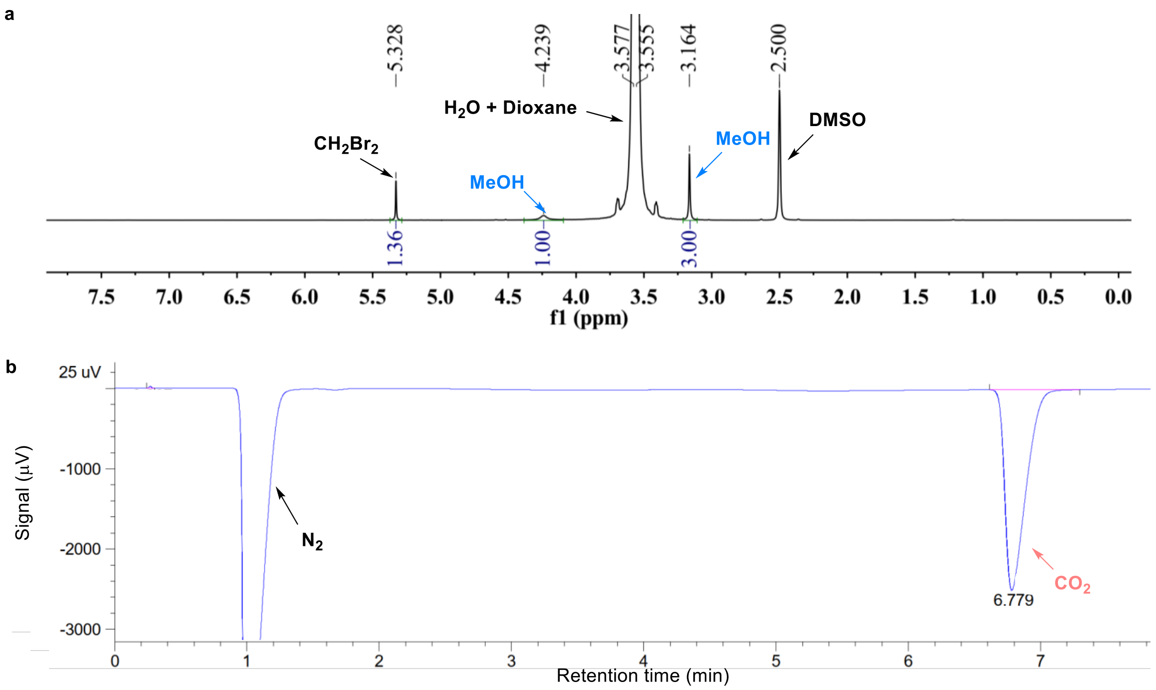

Figure S7. **a**, ¹H NMR spectrum of the generated liquid mixture after POM disproportionation (with DMSO-d_6 as solvent). **b**, Gas chromatogram of the gaseous mixture generated upon POM disproportionation.

Conversion of commercial POM waste into MeOH using Mn-3 and HCOOH

In the glovebox, a 50 mL glass pressure tube was charged with commercial POM waste shavings (90.0 mg, 3.0 mmol), HCOOH (5.7 µL, 0.15 mmol, 5 mol% *vs* POM), 1,4-dioxane (2.0 mL), H_2O (1.0 mL) and a Teflon-coated magnetic stirring bar. The tube was sealed and taken out of the glovebox, and the reaction mixture was stirred at 150 °C for 10 h, during which all solids were consumed. The tube was then allowed to cool to room temperature, was reintroduced into the glovebox, and **Mn-3** (1.8 mg, 0.003 mmol, 0.1 mol% *vs* POM) was added to the reaction mixture. The tube was resealed and removed from the glovebox, and the reaction mixture was stirred at 150 °C for 10 h. The mixture was then allowed to cool to room temperature, and the headspace composition was determined by GC-TCD analysis. The excess gas was then vented off, and the liquid reaction mixture was analyzed by ^1H and ^{13}C NMR spectroscopy.

Table S5. Exploration of reaction conditions for the disproportionation of commercial POM waste catalyzed by **Mn-3**.

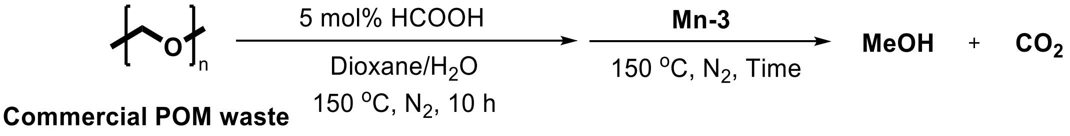

Entry	Commercial POM waste (mmol)	**Mn-3** (mol%)	Dioxane/ H_2O (mL)	Time (h)	CH_2O yield (%)	MeOH yield (%) /TON	CO_2 yield (%)

1	POM clip (3.0)	0.1	2.0/1.0	10	trace	93/620	90
2	Commercial POM waste (5.0)	0.02	2.0/1.0	40	2	89/2967	88
3	Commercial POM waste (5.0)	0.01	2.0/1.0	40	4	87/5800	87
4	Commercial POM waste (10.0)	1×10^{-3}	3.0/1.5	40	65	26/17333	9

Yields were determined by ^{1}H NMR spectroscopy (dibromomethane was used as internal standard).

Attempted conversion of laboratory POM clip into MeOH using Mn-3 and HCOOH in a single-step reaction

In the glovebox, a 50 mL glass pressure tube was charged with laboratory POM clip shavings (90.0 mg, 3.0 mmol), **Mn-3** (0.6 mg, 0.001 mmol, 0.03 mol% *vs* POM), HCOOH (5.7 μL, 0.15 mmol, 5 mol% *vs* POM), 1,4-dioxane (2.0 mL), H_2O (1.0 mL) and a Teflon-coated magnetic stirring bar. The tube was sealed and taken out of the glovebox, and the reaction mixture was stirred at 150 °C for 20 h. The mixture was then allowed to cool to room temperature, the excess gas was vented off, and the solution was analyzed by ^{1}H and ^{13}C NMR spectroscopy.

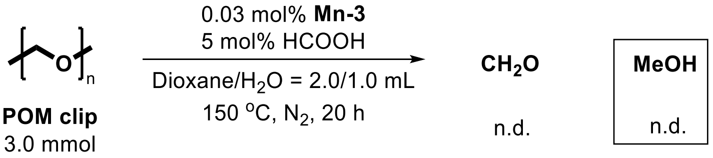

Figure S8. Attempted disproportionation of laboratory POM clip shavings using **Mn-3** as catalyst in the presence of HCOOH.

Methylation of ketones with POM using Mn-3 and H_2

In the glovebox, a 30 mL stainless steel autoclave equipped with a Teflon tube insert was charged with granulated POM (45.0 mg, 1.5 mmol), ketone (0.5 mmol), **Mn-3** (0.6 mg, 0.001 mmol, 0.2 mol% *vs* ketone), NaOH (2.0 mg, 0.05 mmol, 10 mol% *vs* ketone), 1,4-dioxane (2.0 mL), H_2O (1.0 mL) and a Teflon-coated magnetic stirring bar. The autoclave was then removed from the glovebox and pressurized with 30 bar of H_2, after which the resulting mixture was stirred at 150 °C for 20 h. Subsequently, the mixture was allowed to cool to room temperature, the excess gas was vented off, and the mixture was extracted with dichloromethane (3 × 2.0 mL). The obtained extract was subjected to flash column chromatography over silica gel, using a 15:1 petroleum ether/ethyl acetate mixture as the eluent, to eventually give the desired product.

Table S6. Optimization of catalytic conditions for ketone methylation with POM using **Mn-3** and H_2.

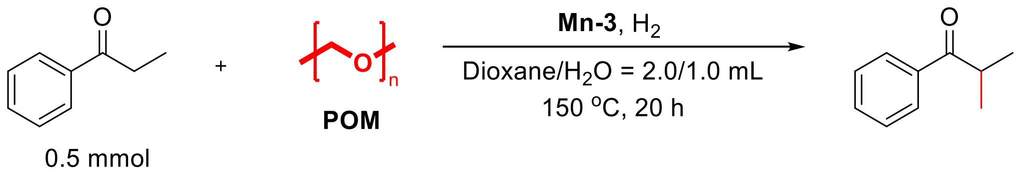

Entry	POM (mmol)	**Mn-3** (mol%)	Base	H_2 pressure (bar)	Reactor	yield (%)
1	1.0	0.3	-	7	Fischer-Porter	20
2	1.0	0.3	100 mol% K_2CO_3	7	Fischer-Porter	15
3	3.0	0.3	-	7	Fischer-Porter	n.d.
4	1.0	0.3	50 mol% NaOH	7	Fischer-Porter	55
5	1.0	0.3	100 mol% NaOH	7	Fischer-Porter	48
6	1.0	0.3	20 mol% ^{t}BuOK	7	Autoclave	4
7	1.0	0.3	20 mol% ^{t}BuOK	20	Autoclave	85
8	1.0	0.3	10 mol% NaOH	20	Autoclave	86
9[a]	1.0	0.3	10 mol% NaOH	20	Autoclave	64
10	1.5	0.2	10 mol% NaOH	20	Autoclave	86
11	1.5	0.2	10 mol% NaOH	30	Autoclave	94

Yields were determined by ^{1}H NMR spectroscopy (dibromomethane was used as internal standard). [a] **Mn-1** instead of **Mn-3**.

Methylation of amines with POM using Mn-3 and H_2

In the glovebox, a 90 mL Fisher-Porter glass pressure tube was charged with granulated POM (90.0 mg, 3.0 mmol), amine (0.5 mmol), **Mn-3** (0.6 mg, 0.001 mmol, 0.2 mol% *vs* amine), 1,4-dioxane (2.0 mL), H_2O (1.0 mL) and a Teflon-coated magnetic stirring bar. The Fisher-Porter tube was then taken out of the glovebox and pressurized with 7 bar of H_2, and the resulting mixture was stirred at 150 °C for 20 h. Subsequently, the mixture was allowed to cool to room temperature, the excess gas was vented off, and the resulting solution was analyzed by ^{1}H and ^{13}CNMR spectroscopy (dibromomethane was used as internal standard).

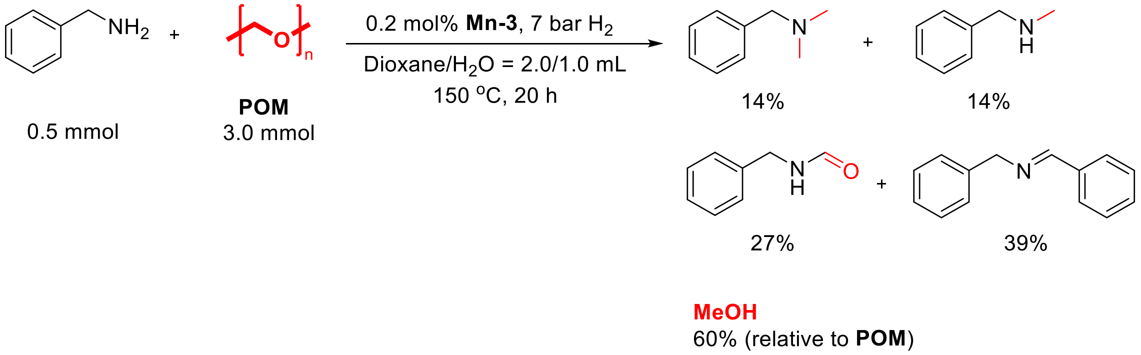

Figure S9. Attempted methylation of amines with POM catalyzed by **Mn-3** under H_2

Methylation of amines with POM using Mn-3 and HCOOH

In the glovebox, a 50 mL glass pressure tube was charged with granulated POM (90.0 mg, 3.0 mmol), HCOOH (2.3 µL, 0.06 mmol, 2 mol% *vs* POM), 1,4-dioxane (2.0 mL), H_2O (1.0 mL) and a Teflon-coated magnetic stirring bar. The tube was sealed and taken out of the glovebox, and the reaction mixture was stirred at 150 °C for 10 h, during which all solids were consumed. The tube was then allowed to cool to room temperature, and was reintroduced into the glovebox, after which the amine substrate (0.5 mmol) and **Mn-3** (0.6 mg, 0.001 mmol, 0.2 mol% *vs* amine) were added to the reaction mixture. The tube was resealed and taken out of the glovebox, and the reaction mixture was stirred at 150 °C for 10 h. The mixture was subsequently allowed to cool to room temperature, the excess gas was vented off, and the mixture was extracted with dichloromethane (3 × 2.0 mL). The obtained extract was subjected to flash column chromatography over silica gel, using a 90:15:4 petroleum ether/ethyl acetate/triethylamine mixture as the eluent, to eventually give the desired product.

Table S7. Optimization of catalytic conditions for amine methylation with POM using **Mn-3** and HCOOH.

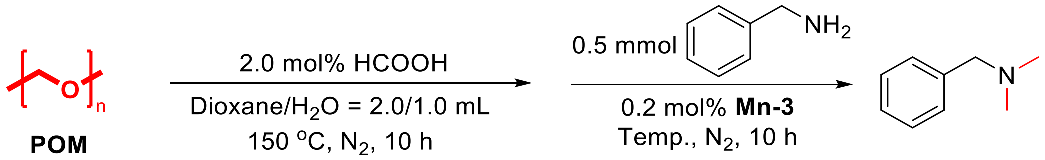

Entry	POM (mmol)	Temp. (°C)	yield (%)
1	1.5	150	35
2	3.0	150	86
3	3.0	110	40

Yields were determined by ^1H NMR spectroscopy (dibromomethane was used as internal standard).

5. Control Experiments

Depolymerization of POM into CH_2O in the absence of a metal-based catalyst

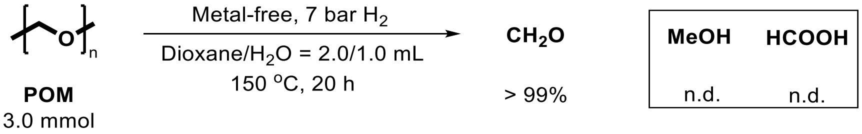

Figure S10. Depolymerization of POM under H_2 in the absence of a metal-based catalyst.

Depolymerization of POM into CH_2O in the absence of a metal-based catalyst under high pressure of N_2

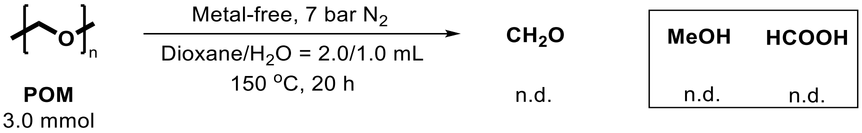

Figure S11. Depolymerization of POM under N_2 in the absence of a metal-based catalyst.

Depolymerization of POM into CH_2O using HCOOH

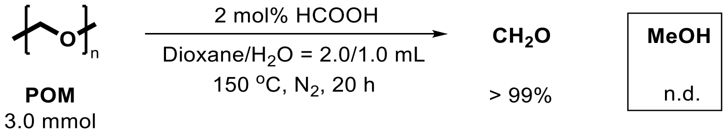

Figure S12. Depolymerization of POM using HCOOH.

Reaction of POM with HCOOH in the absence of a metal-based catalyst

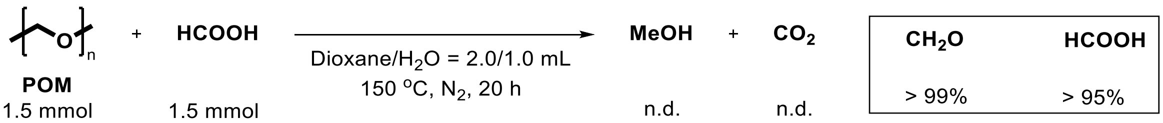

Figure S13. Reaction of POM with HCOOH in the absence of a metal-based catalyst.

Attempted conversion of POM into MeOH using Mn-3 in the absence of H_2 or HCOOH

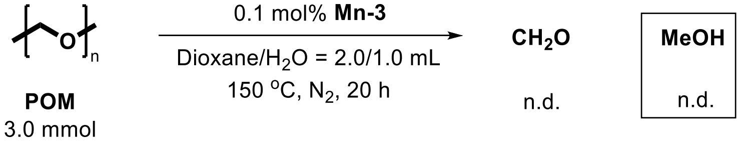

Figure S14. Reaction of POM in the presence of **Mn-3** and absence of H_2 or HCOOH, showing that the Mn-pincer catalyst is not responsible for POM depolymerization.

Conversion of commercial formaldehyde solution into MeOH and CO_2 using Mn-3

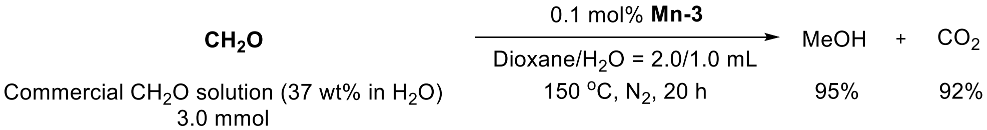

Figure S15. Reaction of commercial formaldehyde solution in the presence of **Mn-3**.

Conversion of POM into MeOH and CO_2 in an open system

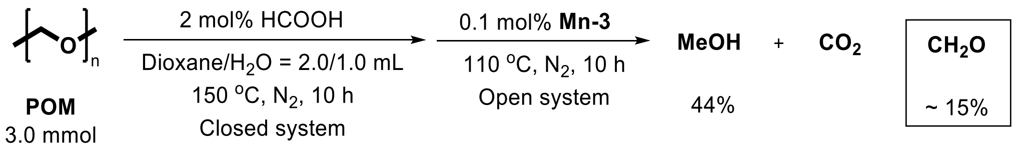

Figure S16. Disproportionation of POM in an open system under N_2 flow.

The open-system reaction was initially conducted at 150 °C, but the solvents and CH_2O were found to have significantly evaporated. The temperature was therefore decreased to 110 °C for this open-system reaction. Nevertheless, some CH_2O was still lost to evaporation.

6. Analytical Data for Catalysis Products

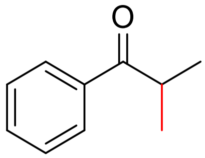

2-Methyl-1-phenylpropan-1-one (1)[10]: This colorless liquid was obtained in 94% isolated yield (69.6 mg). ^{1}H NMR (300 MHz, $CDCl_3$) δ 7.98-7.94 (m, 2H), 7.58-7.52 (m, 1H), 7.48-7.43 (m, 2H), 3.56 (hept, J = 6.9 Hz, 1H), 1.22 (d, J = 6.6 Hz, 6H). ^{13}C{^{1}H} NMR (75 MHz, $CDCl_3$) δ 204.62, 136.35, 132.90, 128.72, 128.43, 35.48, 19.27. GC-EI-MS m/z calcd. for $C_{10}H_{12}O$ $[M]^+$: 148.1, found: 148.1.

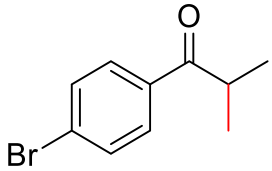

1-(4-Bromophenyl)-2-methylpropan-1-one (2)[11]: This colorless liquid was obtained in 94% isolated yield (96.5 mg). ^{1}H NMR (300 MHz, $CDCl_3$) δ 7.81 (d, J = 8.7 Hz, 2H), 7.59 (d, J = 8.7 Hz, 2H), 3.48 (hept, J = 6.9 Hz, 1H), 1.20 (d, J = 6.6 Hz, 6H). ^{13}C{^{1}H} NMR (75 MHz, $CDCl_3$) δ 203.51, 135.03, 132.04, 130.00, 128.01, 35.53, 19.18. GC-EI-MS m/z calcd. for $C_{10}H_{11}BrO$ $[M]^+$: 226.0, found: 226.0.

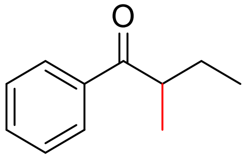

2-Methyl-1-phenylbutan-1-one (3)[10]: This colorless liquid was obtained in 75% isolated yield (63.2 mg). ^{1}H NMR (300 MHz, $CDCl_3$) δ 7.98-7.94 (m, 2H), 7.58-7.52 (m, 1H), 7.49-7.43 (m, 2H), 3.27 (sext, J = 6.7 Hz, 1H), 1.91-1.74 (m, 1H), 1.56-1.42 (m, 1H), 1.19 (d, J = 6.9 Hz, 3H),

0.92 (t, *J* = 7.4 Hz, 3H). $^{13}C\{^1H\}$ NMR (75 MHz, $CDCl_3$) δ 204.60, 136.98, 132.91, 128.73, 128.36, 42.26, 26.81, 16.89, 11.90. GC-EI-MS m/z calcd. for $C_{11}H_{14}O$ $[M]^+$: 162.1, found: 162.1.

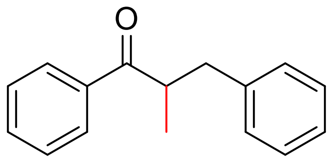

2-Methyl-1,3-diphenylpropan-1-one (4)[10]: This colorless liquid was obtained in 81% isolated yield (90.7 mg). ^1H NMR (300 MHz, $CDCl_3$) δ 7.97-7.93 (m, 2H), 7.59-7.53 (m, 1H), 7.49-7.43 (m, 2H), 7.32-7.17 (m, 5H), 3.83-3.72 (m, 1H), 3.20 (dd, *J* = 13.8, 6.3 Hz, 1H), 2.72 (dd, *J* = 13.8, 7.8 Hz, 1H), 1.23 (d, *J* = 6.9 Hz, 3H). $^{13}C\{^1H\}$ NMR (75 MHz, $CDCl_3$) δ 203.84, 140.08, 136.59, 133.03, 129.21, 128.75, 128.49, 128.40, 126.32, 42.88, 39.50, 17.53. GC-EI-MS m/z calcd. for $C_{16}H_{16}O$ $[M]^+$: 224.1, found: 224.1.

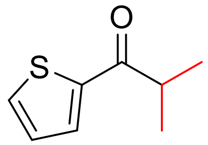

2-Methyl-1-(thiophen-2-yl)propan-1-one (5)[10]: This colorless liquid was obtained in 92% isolated yield (70.8 mg). ^1H NMR (300 MHz, $CDCl_3$) δ 7.72 (dd, *J* = 3.9, 1.2 Hz, 1H), 7.62 (dd, *J* = 5.1, 1.2 Hz, 1H), 7.72 (dd, *J* = 5.1, 3.9 Hz, 1H), 3.39 (hept, *J* = 6.9 Hz, 1H), 1.24 (d, J = 6.9 Hz, 6H). $^{13}C\{^1H\}$ NMR (75 MHz, $CDCl_3$) δ 197.62, 143.82, 133.53, 131.68, 128.19, 37.36, 19.57. GC-EI-MS m/z calcd. for $C_8H_{10}OS$ $[M]^+$: 154.1, found: 154.1.

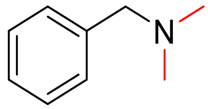

***N,N*-Dimethyl-benzylamine (6)[12]**: This colorless liquid was obtained in 85% isolated yield (57.4 mg). ^1H NMR (300 MHz, $CDCl_3$) δ 7.37-7.24 (m, 5H), 3.44 (s, 2H), 2.26 (s, 6H). $^{13}C\{^1H\}$ NMR (75 MHz, $CDCl_3$) δ 138.98, 129.22, 128.34, 127.14, 64.53, 45.48. GC-EI-MS m/z calcd. for $C_9H_{13}N$ $[M]^+$: 135.1, found: 135.1.

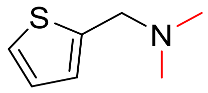

***N,N*-Dimethyl-1-(thiophen-2-yl)methanamine (7)[13]**: This colorless liquid was obtained in 84% isolated yield (59.2 mg). ^1H NMR (300 MHz, $CDCl_3$) δ 7.27-7.21 (m, 1H), 6.96-6.90 (m, 2H), 3.65 (s, 2H), 2.28 (s, 6H). $^{13}C\{^1H\}$ NMR (75 MHz, $CDCl_3$) δ 128.43, 126.48, 125.97, 125.07, 58.51, 45.23. GC-EI-MS m/z calcd. for $C_7H_{11}NS$ $[M]^+$: 141.1, found: 141.1.

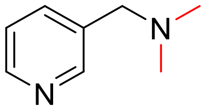

***N,N*-Dimethyl-1-(pyridin-3-yl)methanamine (8)**[12]: This colorless liquid was obtained in 89% isolated yield (60.6 mg). ^{1}H NMR (300 MHz, $CDCl_3$) δ 8.53-8.51 (m, 2H), 7.67 (dt, *J* = 7.8, 2.0 Hz, 1H), 7.28-7.24 (m, 1H), 3.44 (s, 2H), 2.25 (s, 6H). $^{13}C\{^1H\}$ NMR (75 MHz, $CDCl_3$) δ 150.54, 148.79, 136.77, 134.32, 123.47, 61.60, 45.43. GC-EI-MS m/z calcd. for $C_8H_{12}N_2$ $[M]^+$: 136.1, found: 136.1.

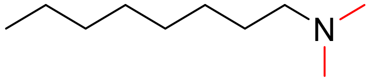

***N,N*-Dimethyloctan-1-amine (9)[14]:** This yellow liquid was obtained in 81% isolated yield (63.6 mg). ^{1}H NMR (300 MHz, $CDCl_3$) δ 2.26-2.21 (m, 8H), 1.50-1.40 (m, 2H), 1.32-1.21 (m, 10H), 0.87 (t, *J* = 6.9 Hz, 3H). $^{13}C\{^1H\}$ NMR (75 MHz, $CDCl_3$) δ 60.14, 45.66, 32.00, 29.74, 29.42, 27.92, 27.68, 22.81, 14.24. GC-EI-MS m/z calcd. for $C_{10}H_{23}N$ $[M]^+$: 157.2, found: 157.2.

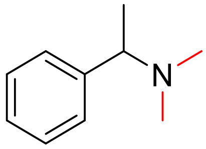

***N,N*-Dimethyl-1-phenylethan-1-amine (10)**[14]: This colorless liquid was obtained in 82% isolated yield (61.1 mg). ^{1}H NMR (300 MHz, $CDCl_3$) δ 7.35-7.21 (m, 5H), 3.25 (q, *J* = 6.7 Hz, 1H), 2.21 (s, 6H), 1.38 (d, *J* = 6.9 Hz, 3H). $^{13}C\{^1H\}$ NMR (75 MHz, $CDCl_3$) δ 144.20, 128.35, 127.68, 127.04, 66.14, 43.38, 20.37. GC-EI-MS m/z calcd. for $C_{10}H_{15}N$ $[M]^+$: 149.1, found: 149.1.

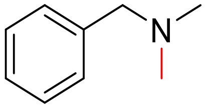

***N,N*-Dimethyl-benzylamine (11)**[12]: This colorless liquid was obtained in 97% isolated yield (65.5 mg). ^{1}H NMR (300 MHz, $CDCl_3$) δ 7.35-7.22 (m, 5H), 3.42 (s, 2H), 2.24 (s, 6H). $^{13}C\{^1H\}$ NMR (75 MHz, $CDCl_3$) δ 138.99, 129.24, 128.37, 127.17, 64.55, 45.50. GC-EI-MS m/z calcd. for $C_9H_{13}N$ $[M]^+$: 135.1, found: 135.1.

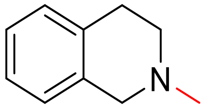

2-Methyl-1,2,3,4-tetrahydroisoquinoline (12)[14]: This yellow liquid was obtained in 80% isolated yield (58.8 mg). ^{1}H NMR (300 MHz, $CDCl_3$) δ 7.14-7.08 (m, 3H), 7.03-6.99 (m, 1H), 3.58 (s, 2H), 2.93 (t, *J* = 6.0 Hz, 2H), 2.69 (t, *J* = 6.0 Hz, 2H), 2.46 (s, 3H). $^{13}C\{^1H\}$ NMR (75 MHz, $CDCl_3$) δ 134.89, 133.95, 128.76, 126.54, 126.22, 125.69, 58.15, 53.07, 46.28, 29.38. GC-EI-MS m/z calcd. for $C_{10}H_{13}N$ $[M]^+$: 147.1, found: 147.1.

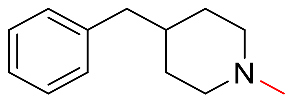

4-Benzyl-1-methylpiperidine (13)[15]: This colorless liquid was obtained in 95% isolated yield (89.8 mg). ^{1}H NMR (300 MHz, $CDCl_3$) δ 7.32-7.27 (m, 2H), 7.23-7.15 (m, 3H), 2.88-2.81 (m, 2H), 2.56 (d, *J* = 6.9 Hz, 2H), 2.27 (s, 2H), 1.88 (td, *J* = 11.7, 2.4 Hz, 2H), 1.68-1.63 (m, 2H), 1.57-1.45 (m, 1H), 1.41-1.31 (m, 2H). $^{13}C\{^1H\}$ NMR (75 MHz, $CDCl_3$) δ 140.77, 129.20, 128.22, 125.85, 56.03, 46.52, 43.28, 37.42, 32.35. GC-EI-MS m/z calcd. for $C_{13}H_{18}N$ $[M]^+$: 189.2, found: 189.2.

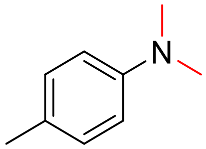

***N*,*N*,4-Trimethylaniline (14)**[16]: This colorless liquid was obtained in 50% isolated yield (33.8 mg). ^{1}H NMR (300 MHz, $CDCl_3$) δ 7.07 (d, *J* = 8.4 Hz, 2H), 6.71 (d, *J* = 8.7 Hz, 2H), 2.91 (s, 6H), 2.27 (s, 3H). $^{13}C\{^1H\}$ NMR (75 MHz, $CDCl_3$) δ 148.97, 129.73, 126.28, 113.38, 41.23, 20.38. GC-EI-MS m/z calcd. for $C_9H_{13}N$ $[M]^+$: 135.1, found: 135.1.

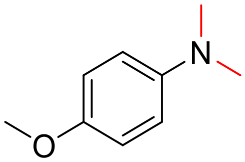

4-Methoxy-*N*,*N*-dimethylaniline (15)[16]: This colorless liquid was obtained in 58% isolated yield (43.8 mg). ^{1}H NMR (300 MHz, $CDCl_3$) δ 6.85 (d, *J* = 9.3 Hz, 2H), 6.76 (d, *J* = 9.3 Hz, 2H), 3.77 (s, 6H), 2.87 (s, 3H). $^{13}C\{^1H\}$ NMR (75 MHz, $CDCl_3$) δ 152.16, 145.92, 115.07, 114.79, 55.91, 41.98. GC-EI-MS m/z calcd. for $C_9H_{13}NO$ $[M]^+$: 151.1, found: 151.1.

7. NMR Spectra of Catalysis Products

Product 1

^{1}H NMR (300 MHz, $CDCl_3$)

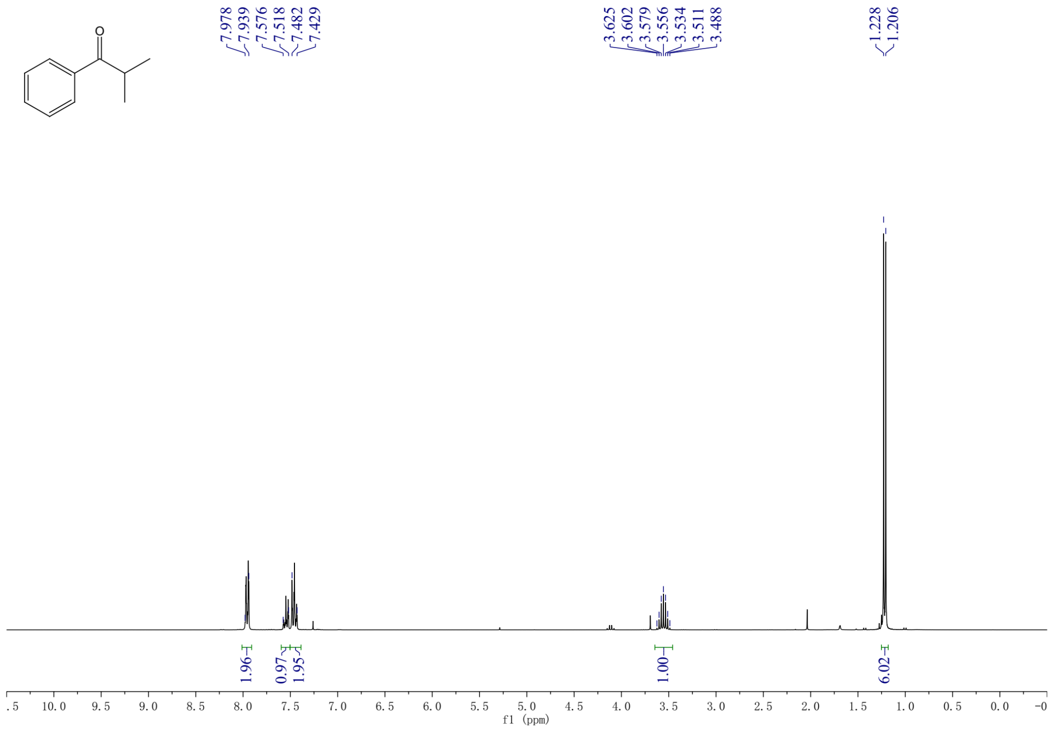

^{13}C{^{1}H} NMR (75 MHz, $CDCl_3$)

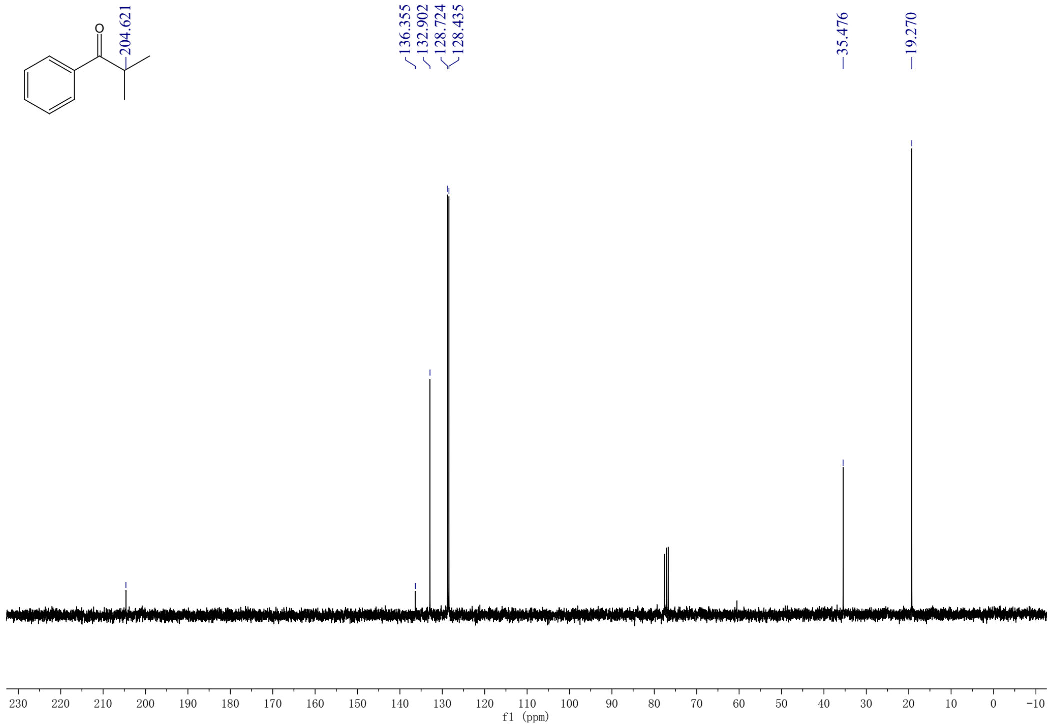

Product 2

^{1}H NMR (300 MHz, $CDCl_3$)

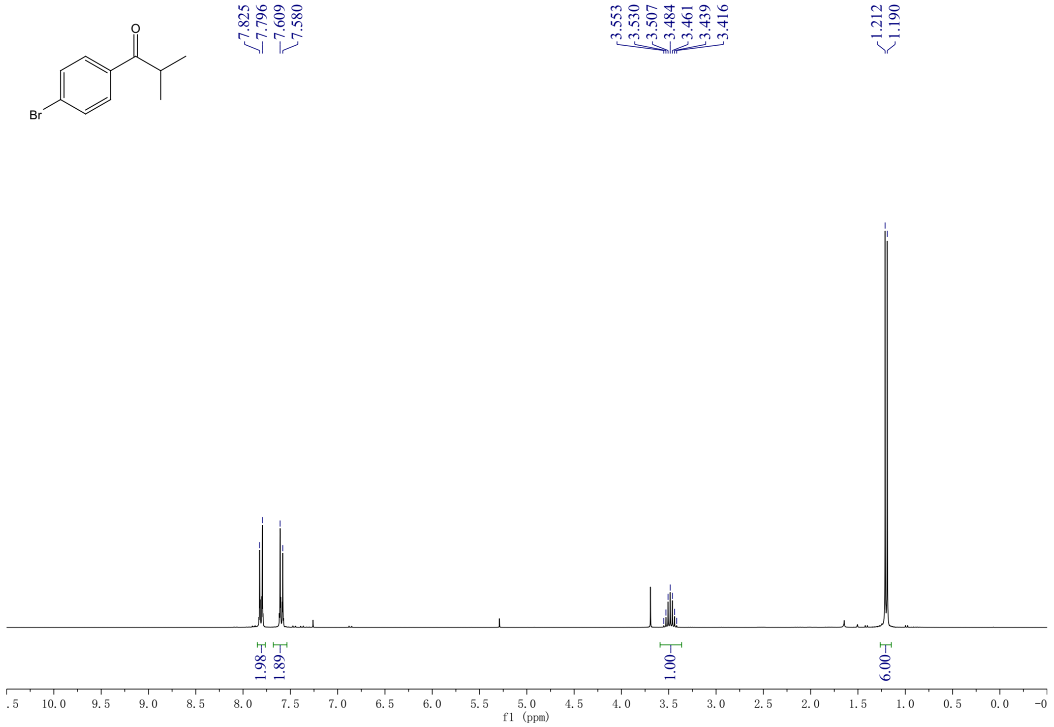

^{13}C{^{1}H} NMR (75 MHz, $CDCl_3$)

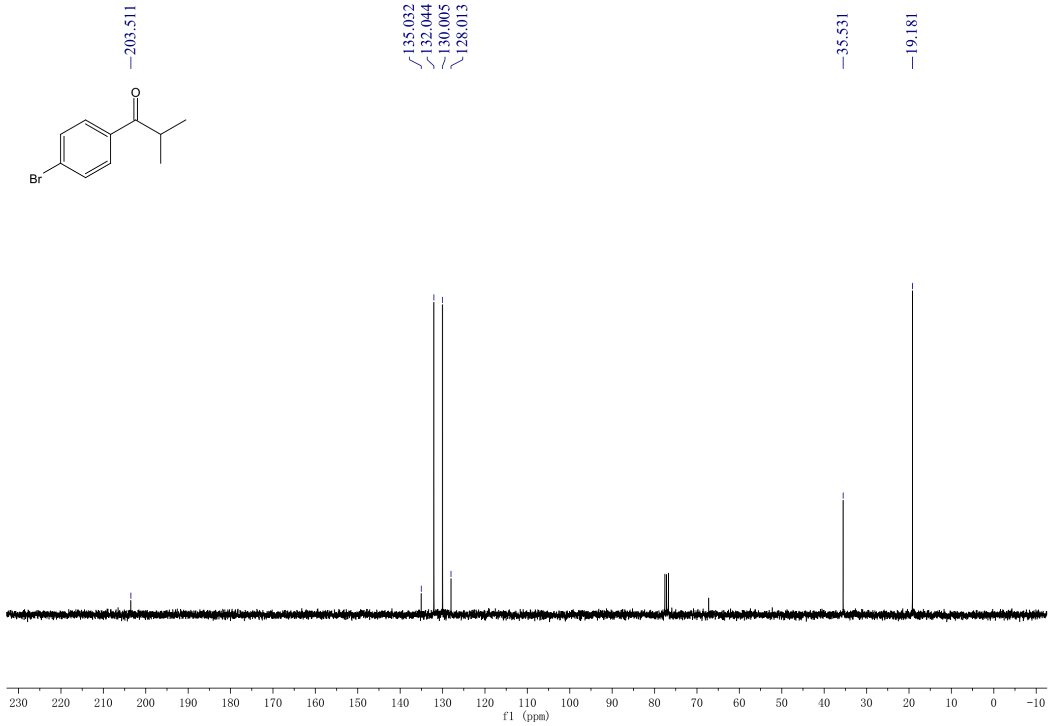

Product 3

^{1}H NMR (300 MHz, $CDCl_3$)

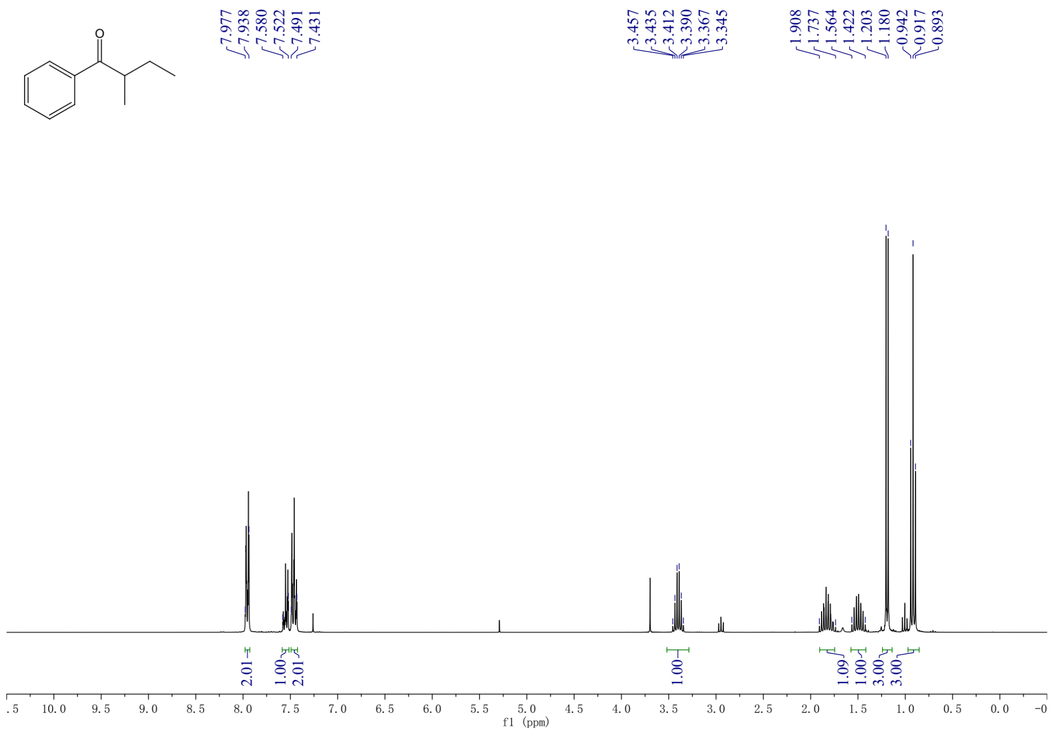

$^{13}C\{^{1}H\}$ NMR (75 MHz, $CDCl_3$)

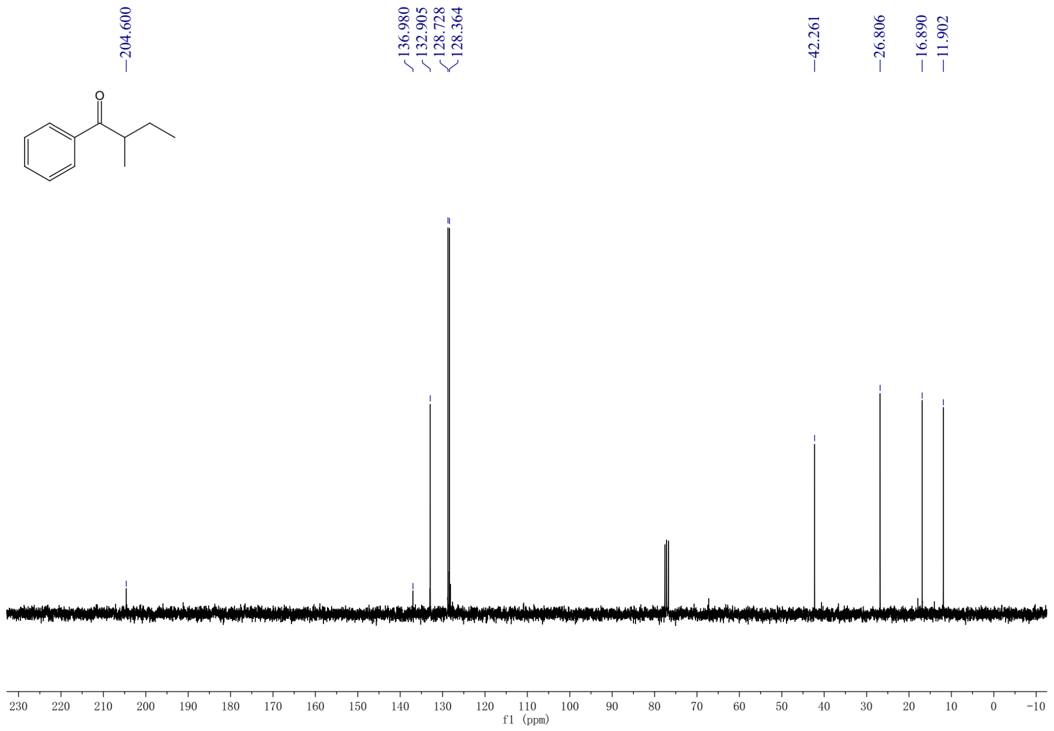

Product 4

^{1}H NMR (300 MHz, $CDCl_3$)

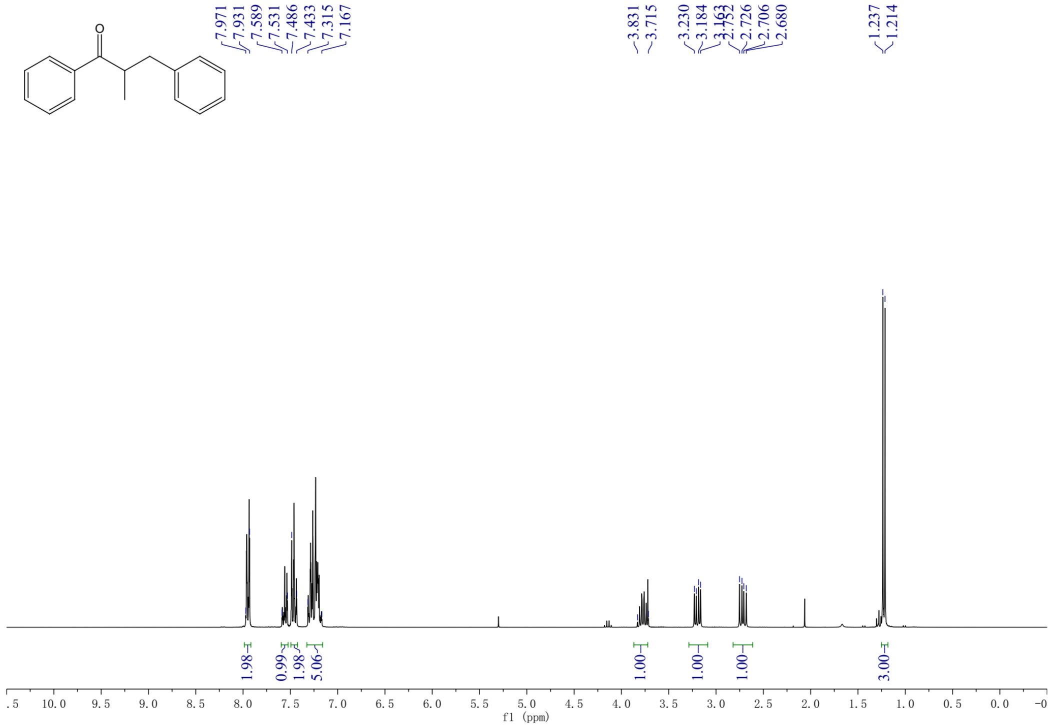

^{13}C{^{1}H} NMR (75 MHz, $CDCl_3$)

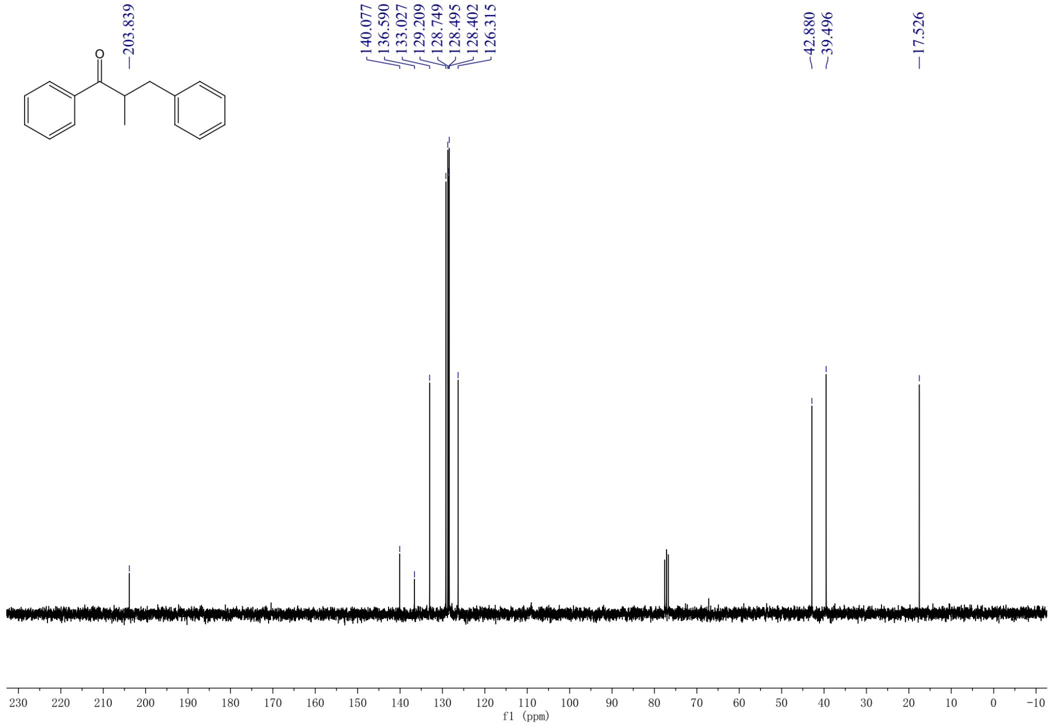

Product 5

^{1}H NMR (300 MHz, $CDCl_3$)

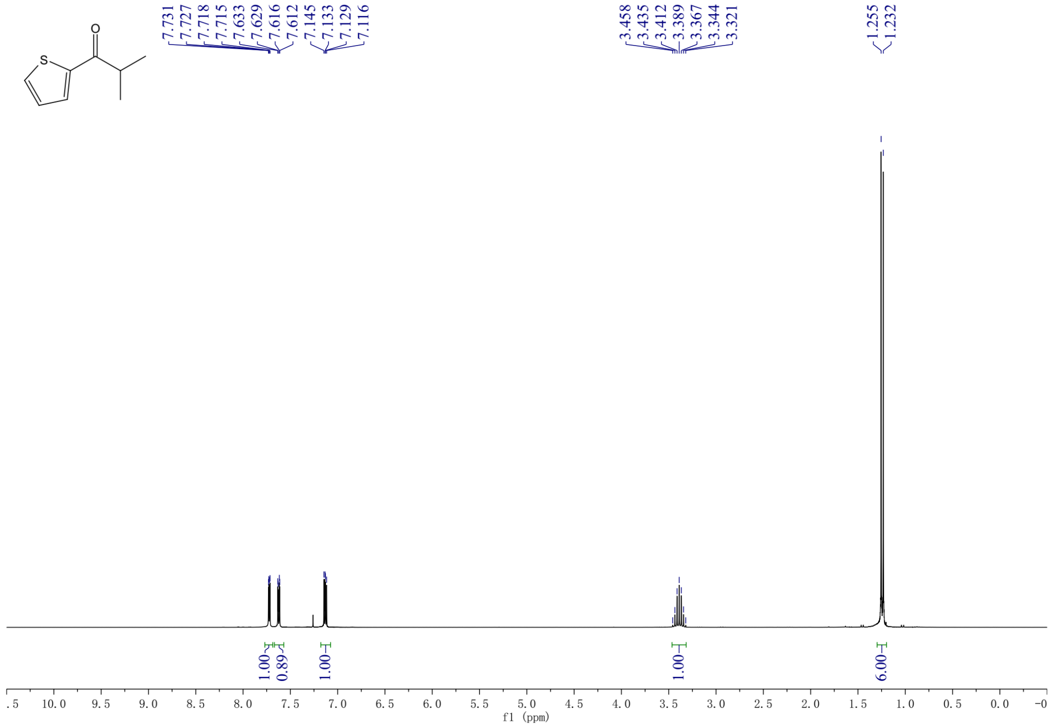

$^{13}C\{^{1}H\}$ NMR (75 MHz, $CDCl_3$)

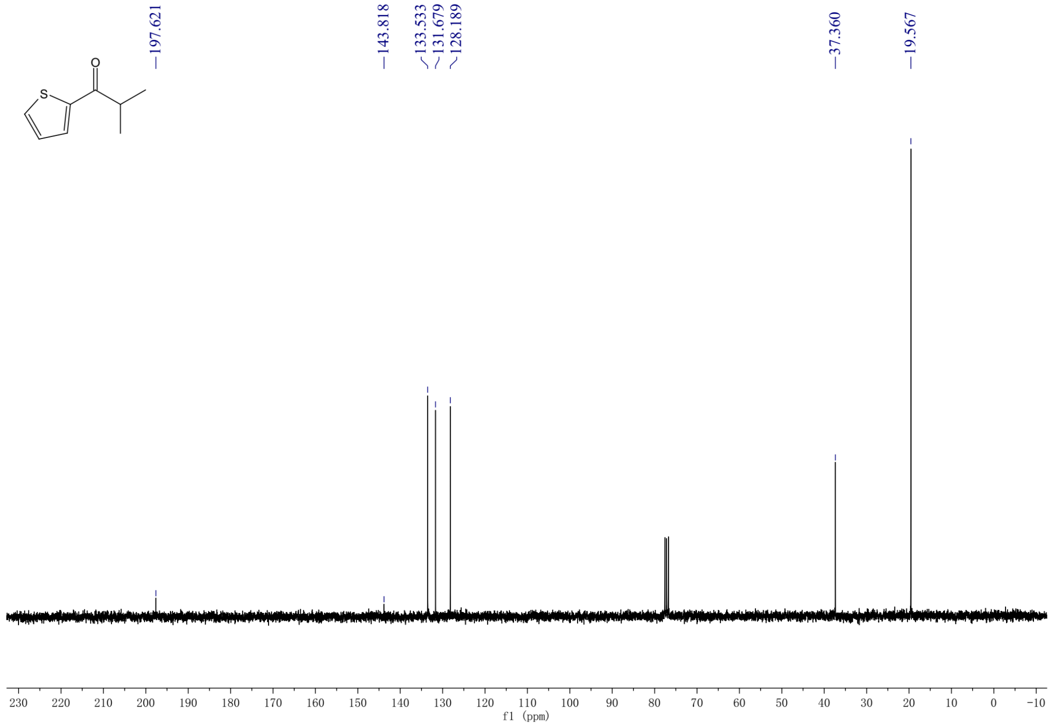

Product 6

^1H NMR (300 MHz, $CDCl_3$)

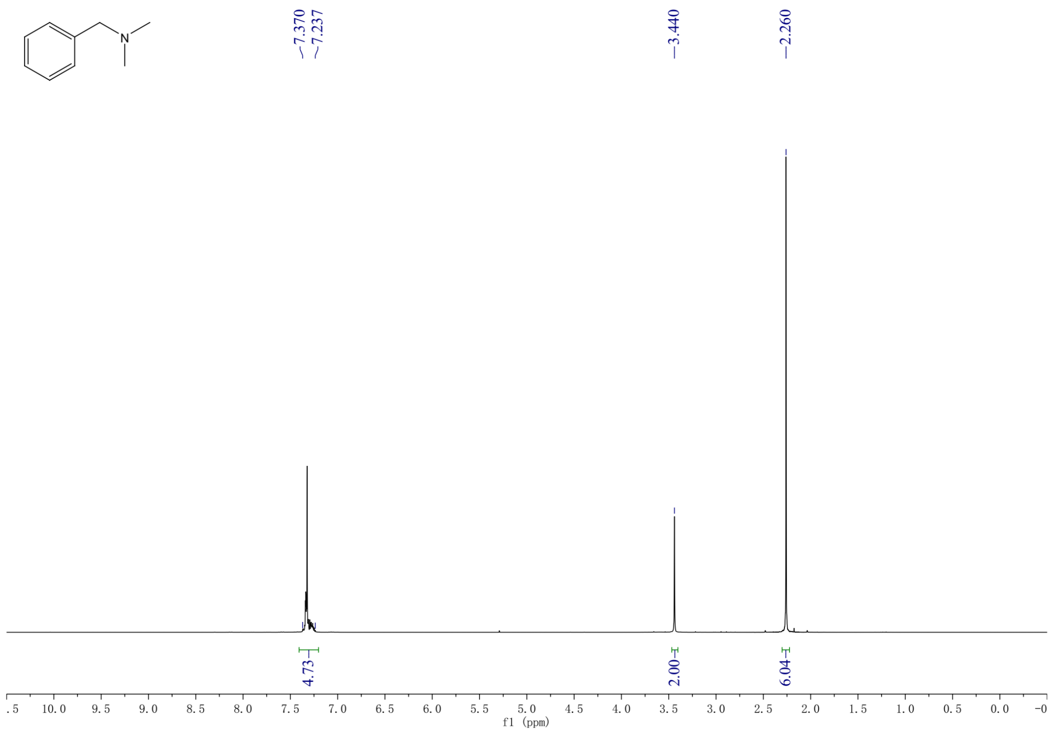

$^{13}C\{^1H\}$ NMR (75 MHz, $CDCl_3$)

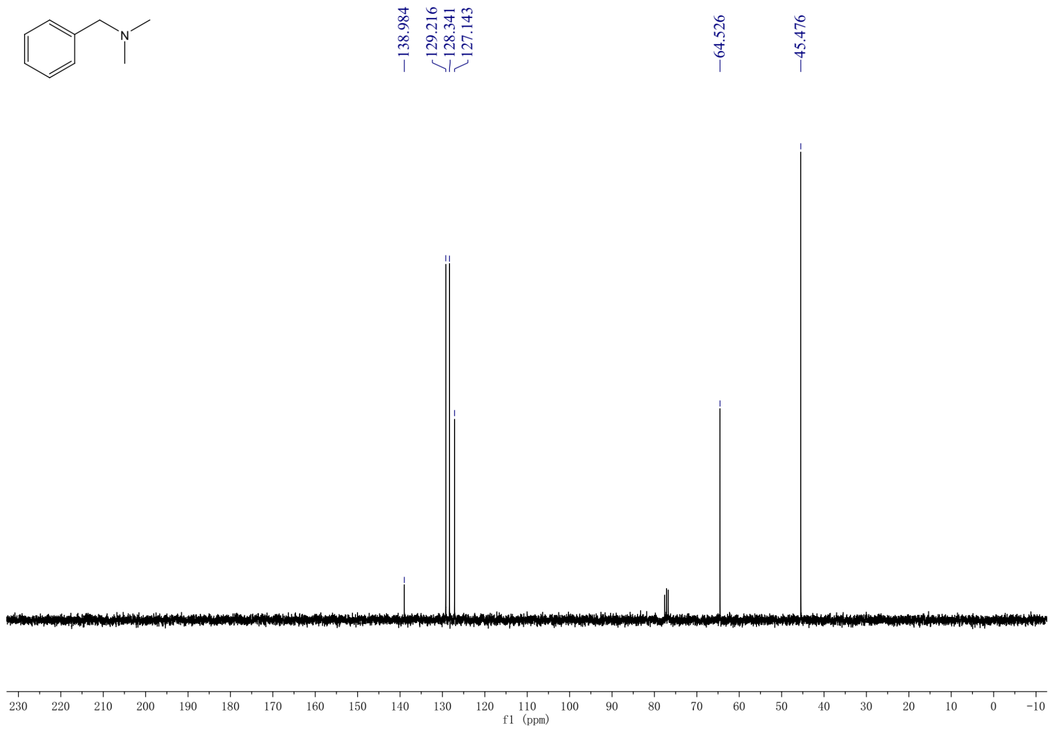

Product 7

^1H NMR (300 MHz, $CDCl_3$)

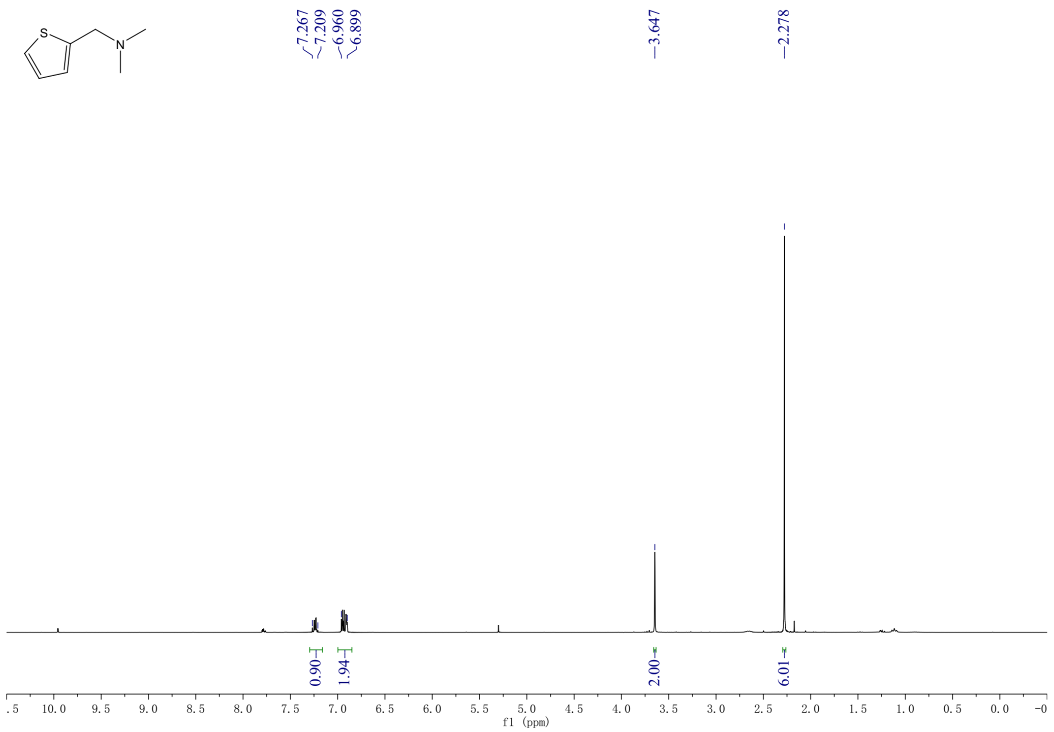

$^{13}C\{^1H\}$ NMR (75 MHz, $CDCl_3$)

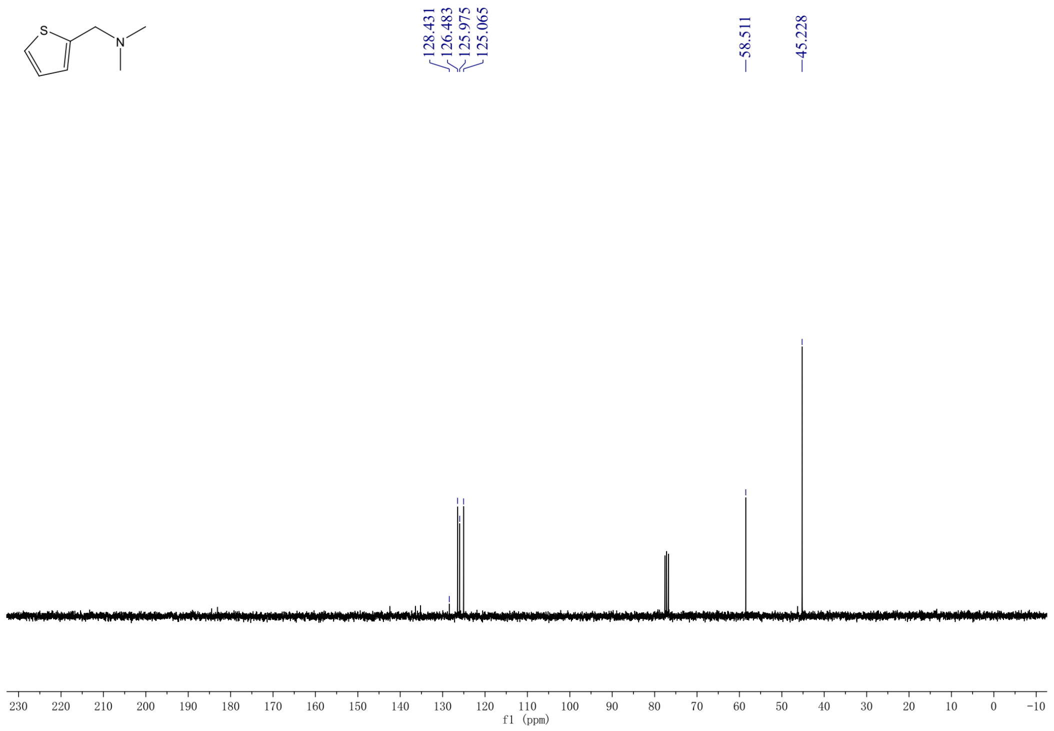

Product 8

^{1}H NMR (300 MHz, $CDCl_3$)

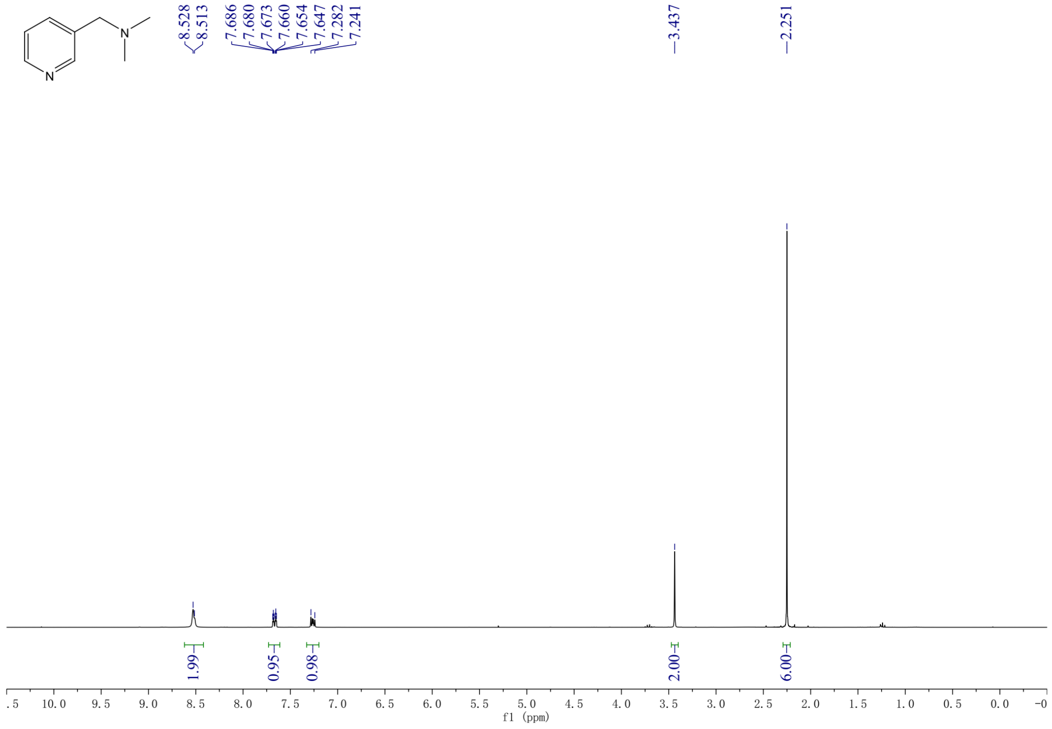

$^{13}C\{^{1}H\}$ NMR (75 MHz, $CDCl_3$)

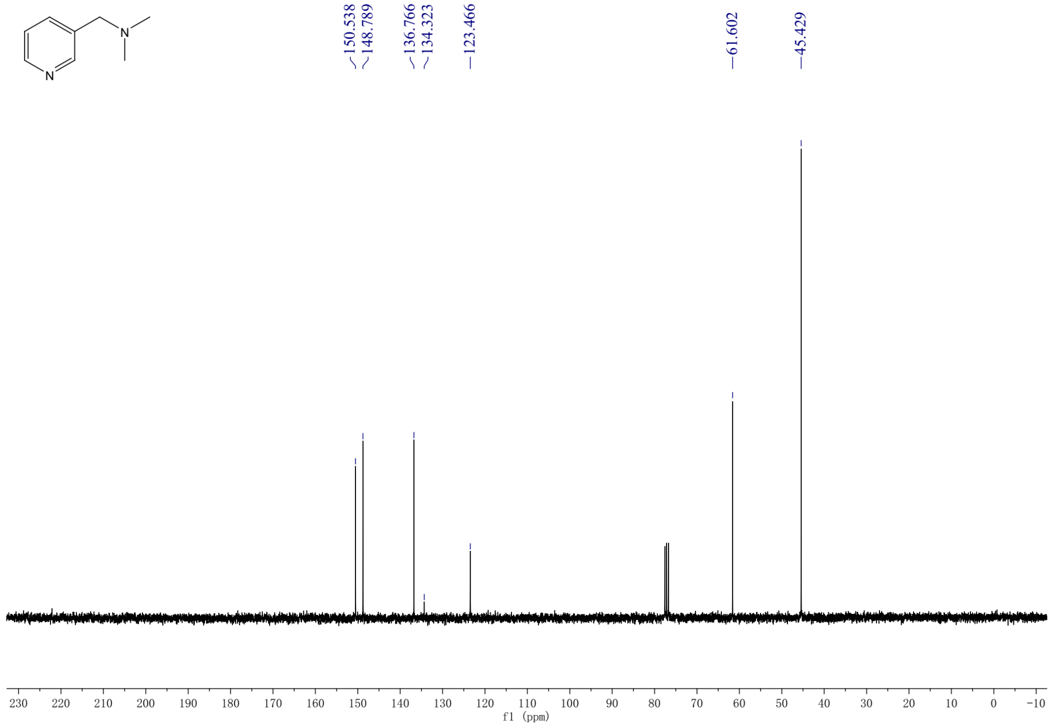

Product 9

^{1}H NMR (300 MHz, $CDCl_3$)

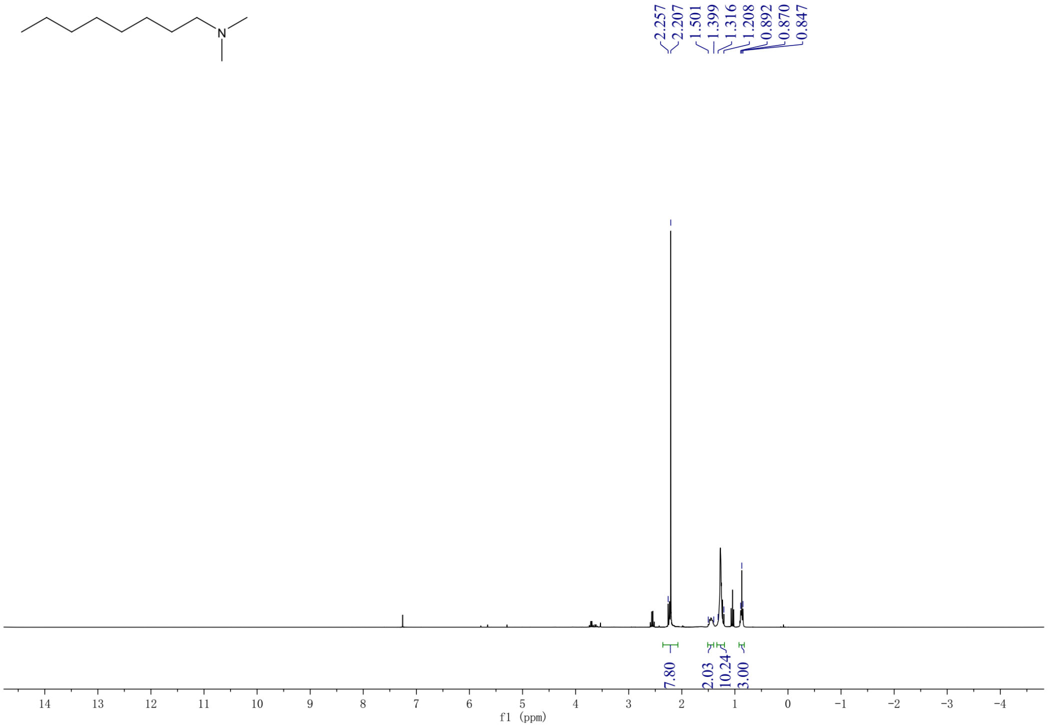

^{13}C{^{1}H} NMR (75 MHz, $CDCl_3$)

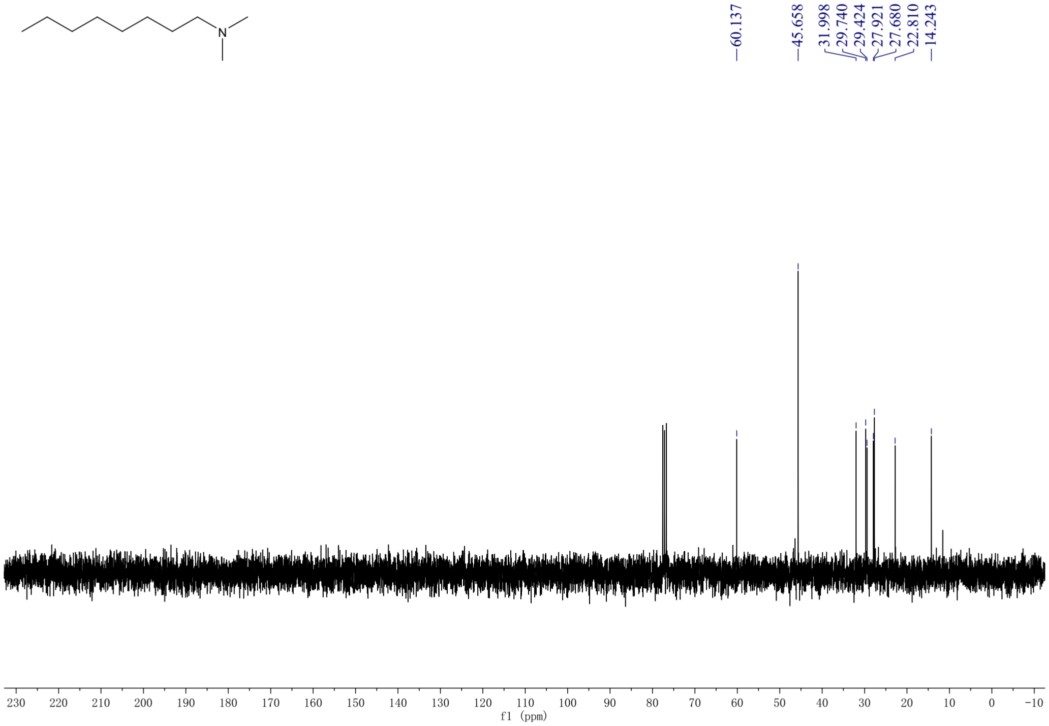

Product 10

^{1}H NMR (300 MHz, $CDCl_3$)

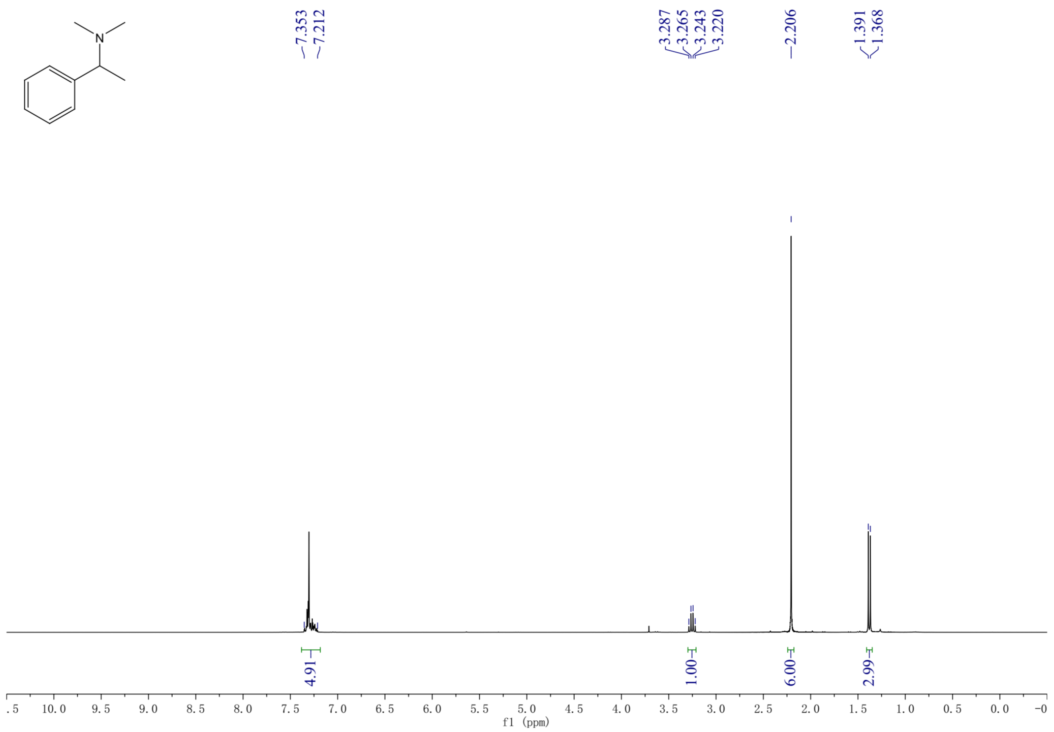

$^{13}C\{^{1}H\}$ NMR (75 MHz, $CDCl_3$)

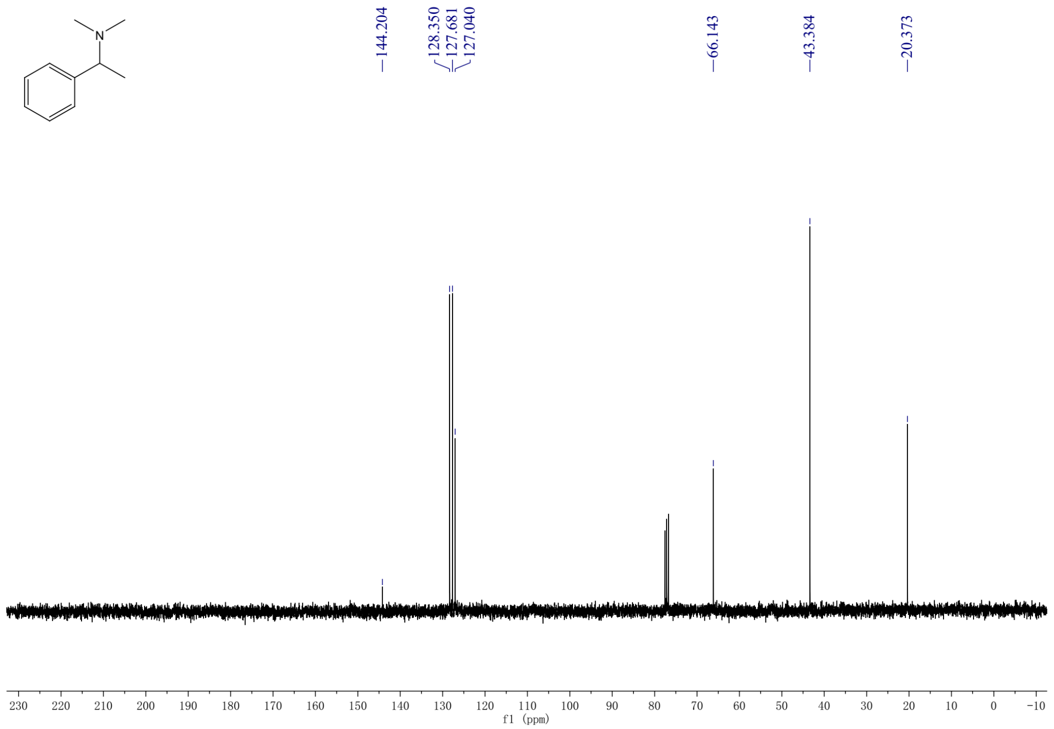

Product 11

^{1}H NMR (300 MHz, $CDCl_3$)

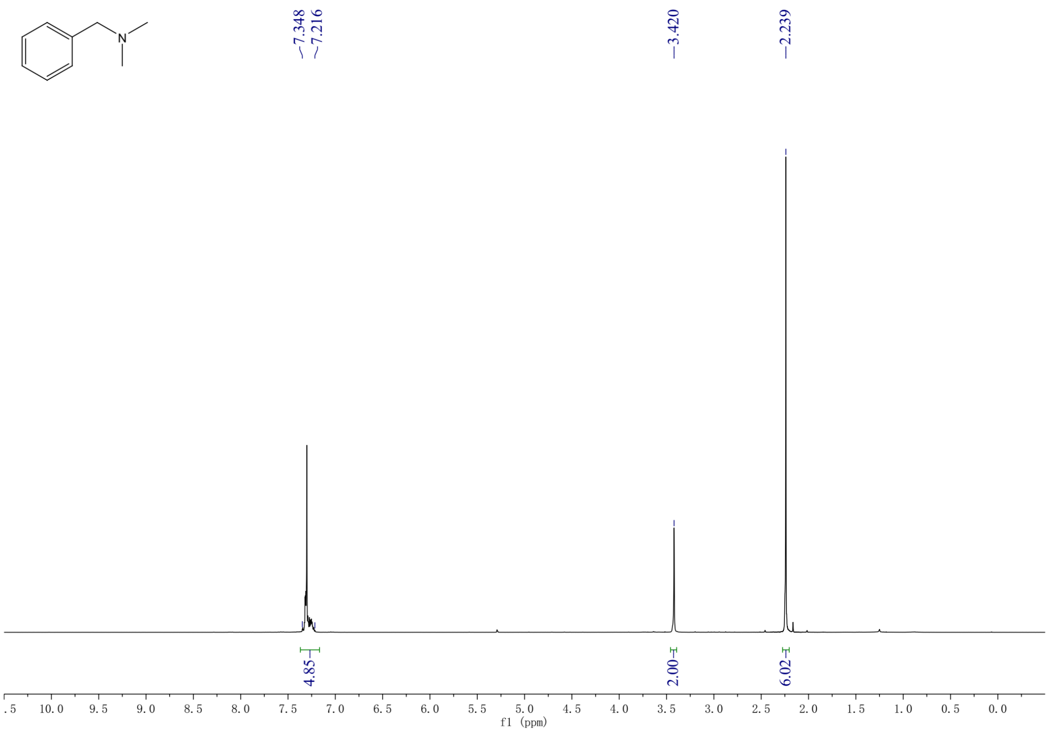

^{13}C{^{1}H} NMR (75 MHz, $CDCl_3$)

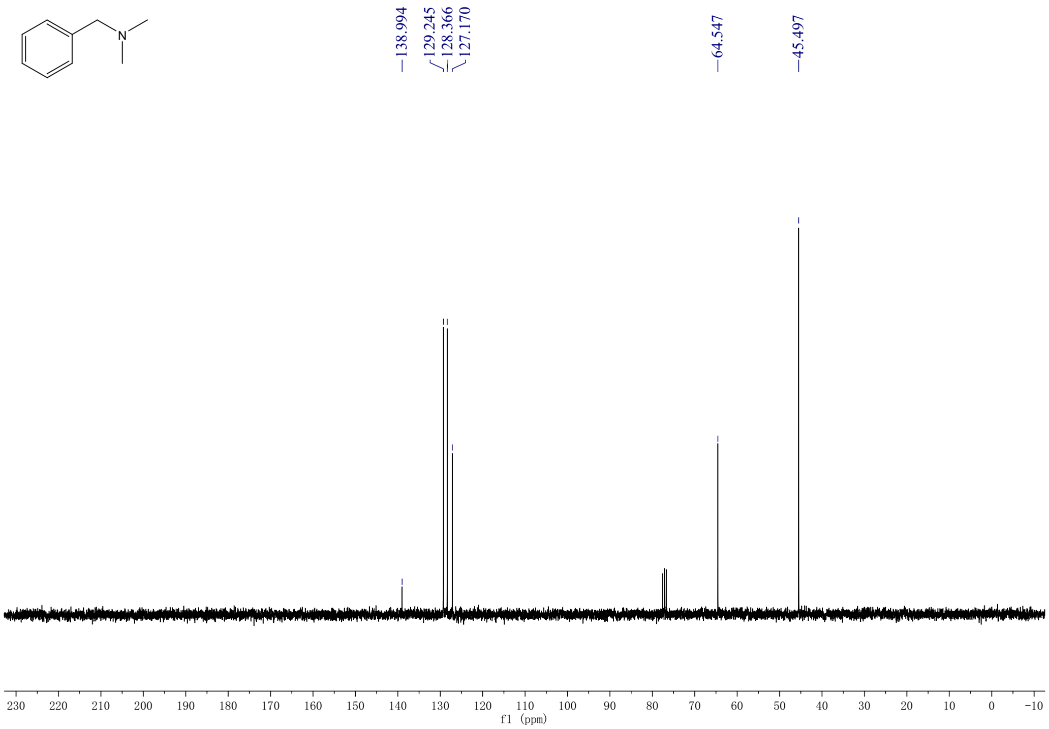

Product 12

^{1}H NMR (300 MHz, $CDCl_3$)

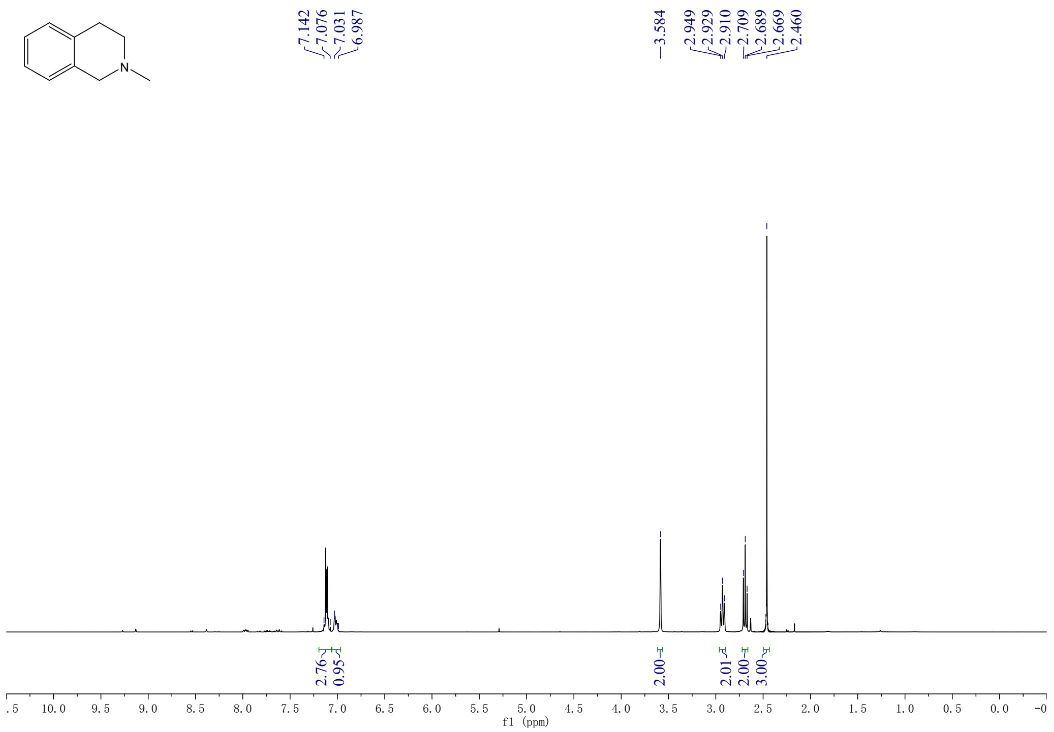

$^{13}C\{^{1}H\}$ NMR (75 MHz, $CDCl_3$)

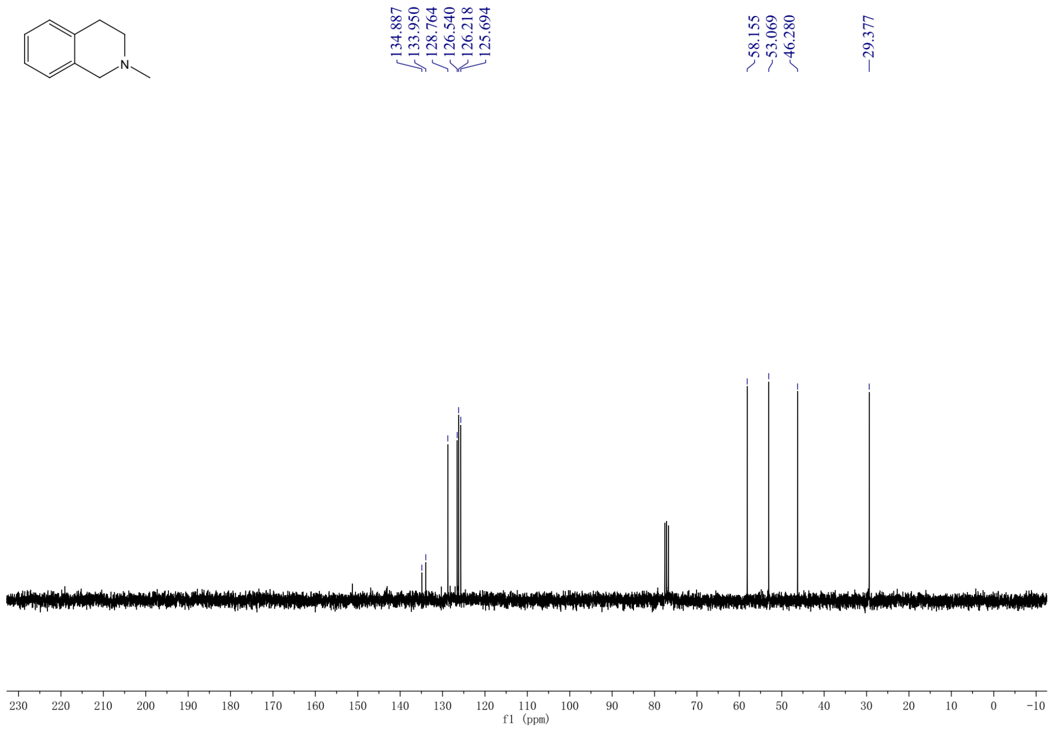

Product 13

^{1}H NMR (300 MHz, $CDCl_3$)

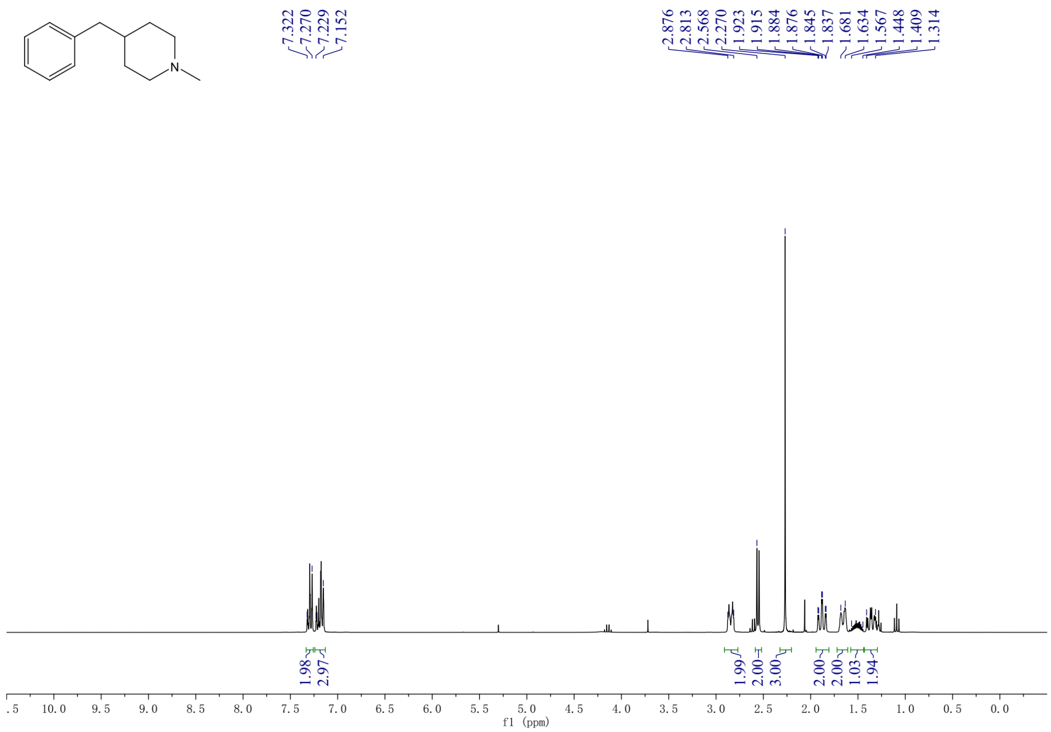

^{13}C{^{1}H} NMR (75 MHz, $CDCl_3$)

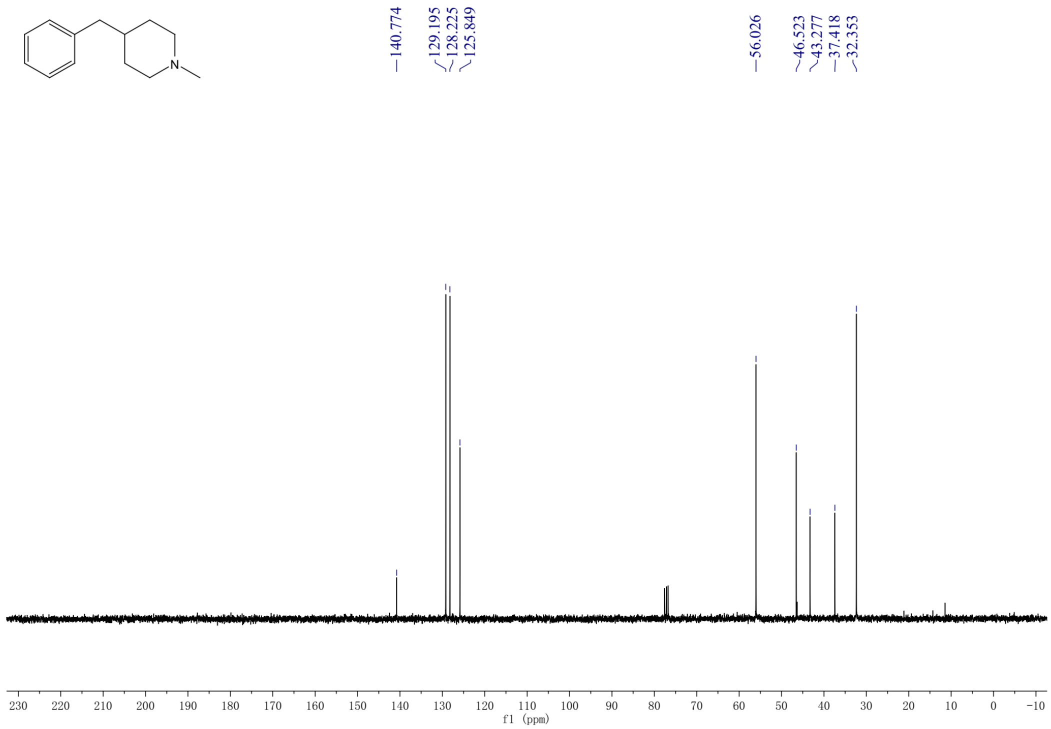

Product 14

^{1}H NMR (300 MHz, $CDCl_3$)

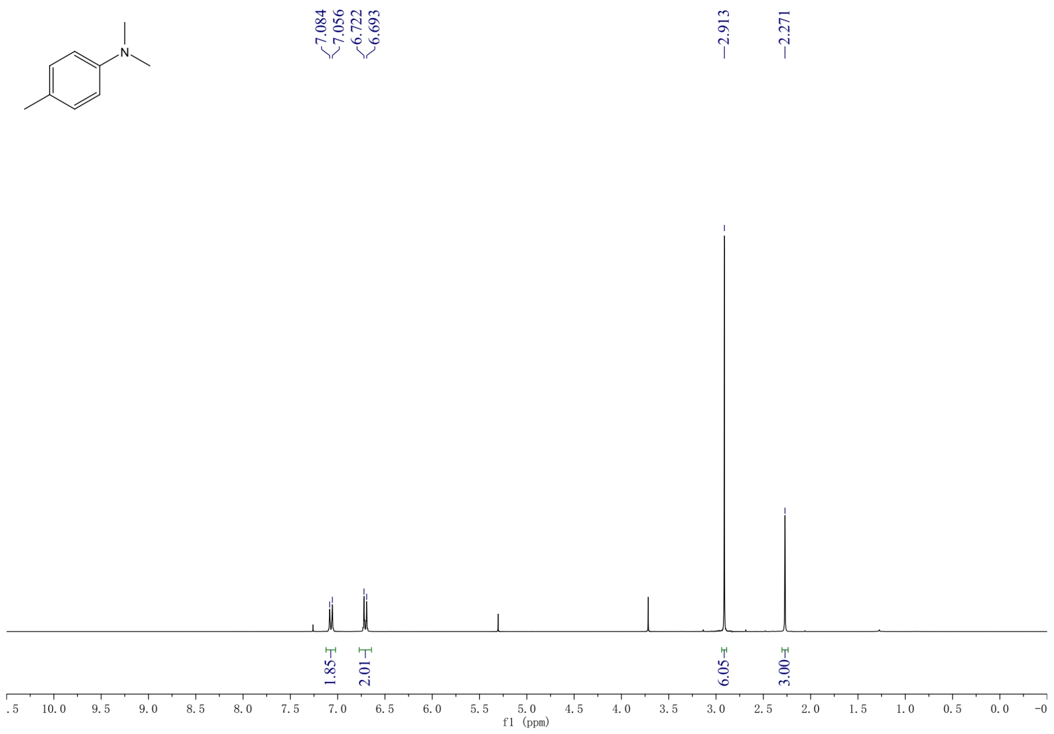

$^{13}C\{^{1}H\}$ NMR (75 MHz, $CDCl_3$)

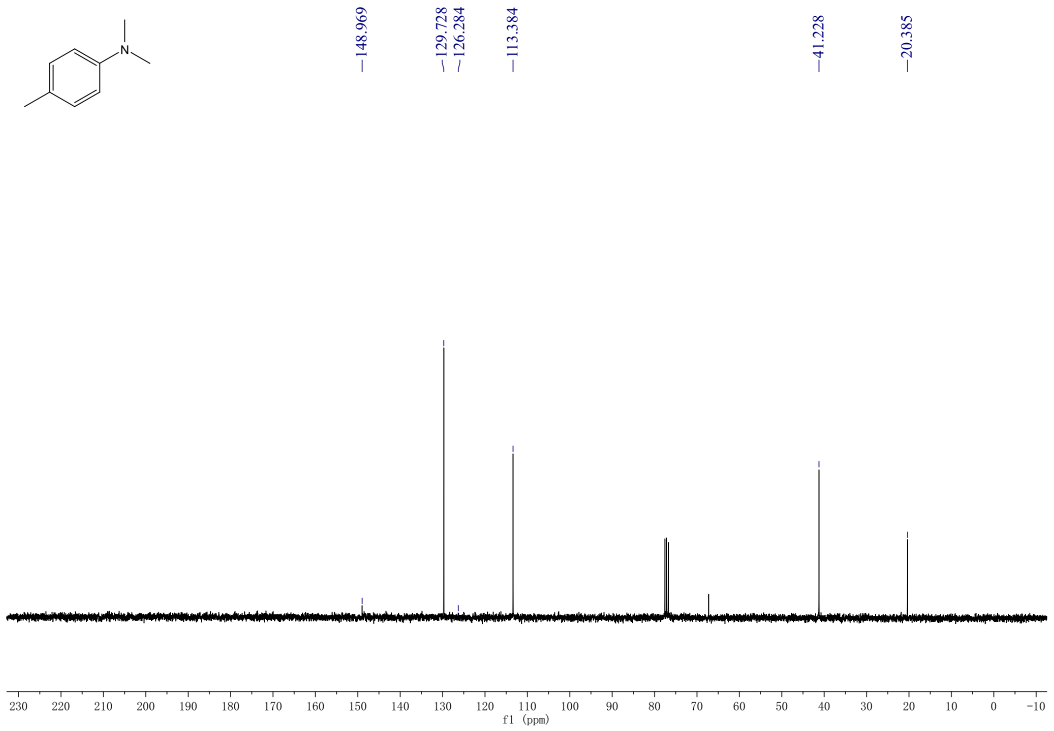

Product 15

^{1}H NMR (300 MHz, $CDCl_3$)

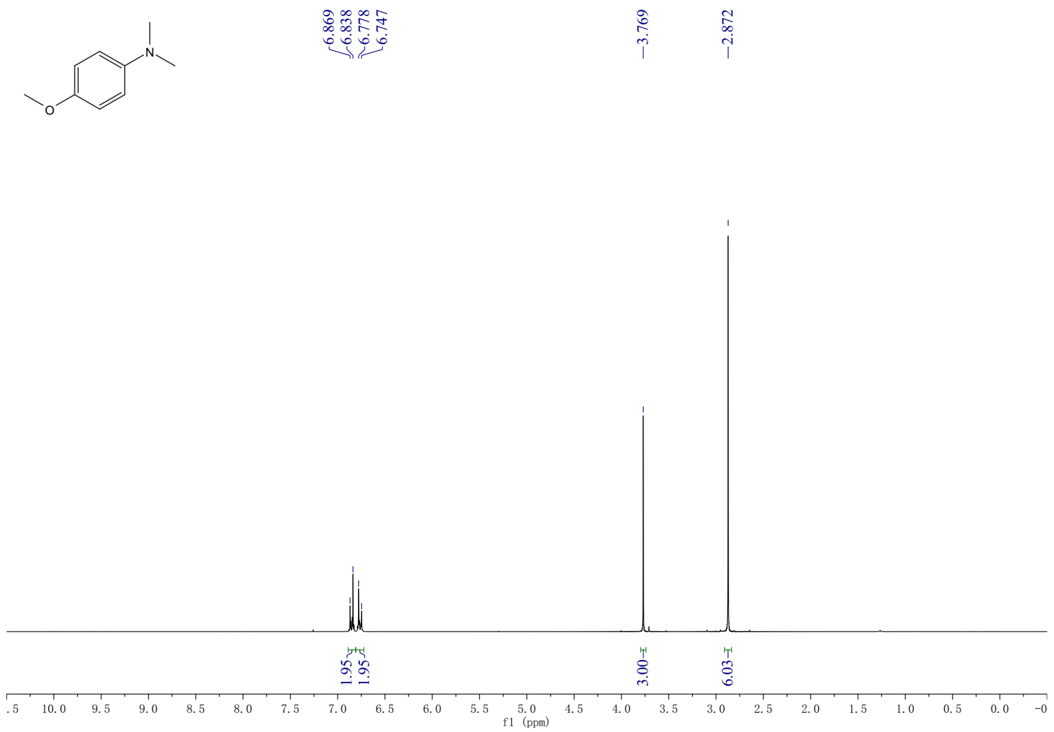

$^{13}C\{^{1}H\}$ NMR (75 MHz, $CDCl_3$)

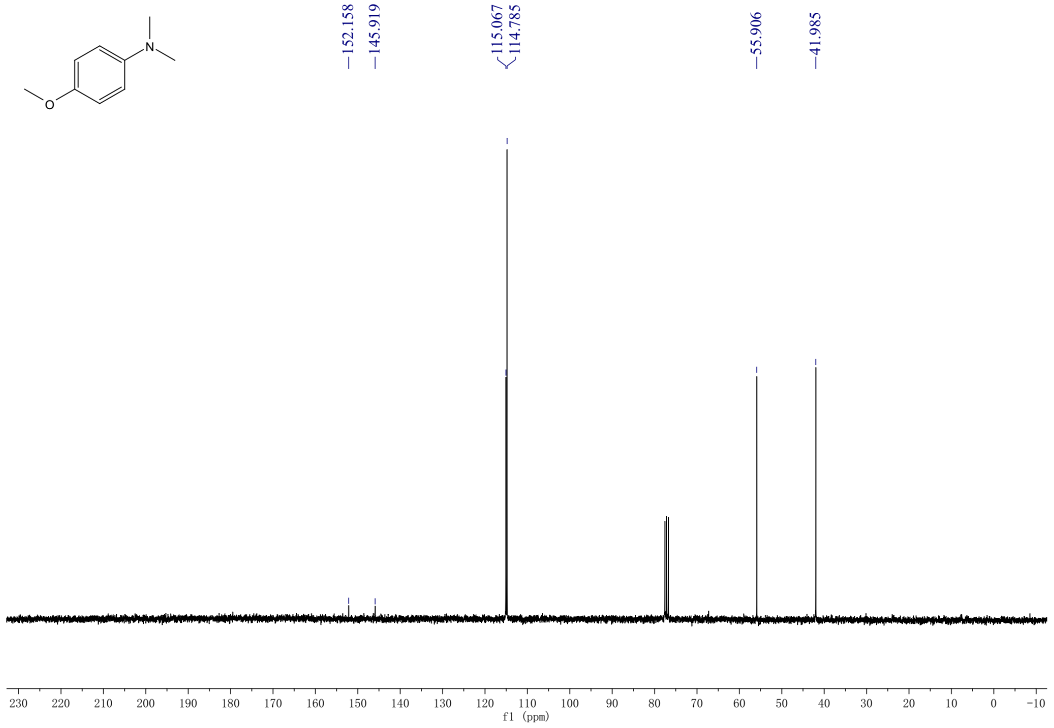

8. Computational Details

Density functional theory (DFT) calculations were performed with Gaussian 16 (C.01 revision),[17] using Truhlar's M06-L functional,[18] the triple-ξ def2-TZVP basis set,[19] W06 density fitting,[20] and Grimme's D3(0) empirical dispersion correction.[21] Frequency calculations at this level of theory were run at 423.15K to confirm stationary points and transition states, and to obtain thermodynamic corrections. Single point energies of the M06-L optimized structures were computed with ORCA (5.0.3),[22] using the range-separated meta-GGA hybrid functional ωB97M-V of the Head-Gordon group,[23] including dispersion correction,[24,25] together with the triple-ξ def2-TZVPP basis set,[19] and the corresponding auxiliary basis sets, def2/J[20] and def2-TZVPP/C,[26] for RIJCOSX density fitting. The functional and basis set selections are based on recent benchmark studies.[27] The SMD solvation model of Truhlar and coworkers was used in all calculations (optimization and single point),[28] using water as an implicit solvent.

Gibbs free energies at 423.15 K were computed by adding the free energy correction term from the frequency calculation to the single point energy in water, according to the following equation:

$$\mathbf{G^{o\omega B97M\text{-}V}_{(water,\ 423.15K)} = E^{\omega B97M\text{-}V}_{water} + corr^{\mathit{M06\text{-}L}}_{\mathit{freq}(water,\ 1\ atm,\ 423.15K)}}$$

where $\mathbf{E^{\omega B97M\text{-}V}_{water}}$ is the single point energy, and $\mathbf{corr^{\mathit{M06\text{-}L}}_{\mathit{freq}}}$ is the thermal correction to the Gibbs free energy from the frequency calculation (at T = 423.15 K and P = 1 atm).

Free energy values (G^o) were corrected to account for changes in standard states ($G^o \rightarrow G$).

Standard state corrections[29] were employed, such that all species are treated as 1 M (using an ideal gas approximation), with the exception of H_2O (10 M) and H_2 (maintained as 1 atm).[30-32]

Table S8. Energy data for calculated complexes, transition states, substrates and products.

**Structure**	$\mathbf{E^{\omega B97M\text{-}V}_{water}}$	$\mathbf{G^{o\omega B97M\text{-}V}_{(water,\ 423.15K)}}$	**Imaginary Frequency**	**G T = 423.15K**
Energy Unit	Hartree	Hartree	cm^{-1}	kcal
**Mn-3**	-3390.738816	-3390.30264	-	-2127412.217
**Mn-7**	-3390.723428	-3390.291033	-	-2127404.934
**Mn-8**	-3391.93374	-3391.479814	-	-2128150.894
**Mn-9**	-3506.494541	-3506.010248	-	-2200018.741
**Mn-10**	-3581.755724	-3581.266013	-	-2247241.733
**Mn-11**	-3580.599375	-3580.13508	-	-2246532.073
**Mn-12**	-3580.578572	-3580.117947	-	-2246521.322
$\mathbf{TS_{7,8}}$	-3391.881631	-3391.433467	-1087.7352	-2128121.811
$\mathbf{TS_{8,9}}$	-3506.447501	-3505.963655	-171.3795	-2199989.504
$\mathbf{TS_{9,7}}$	-3506.467045	-3505.987433	-868.9191	-2200004.424
$\mathbf{TS_{7,10}}$	-3581.724267	-3581.242572	-460.2160	-2247227.024
$\mathbf{TS_{10,8}}$	-3581.70668	-3581.219303	-188.5387	-2247212.423
$\mathbf{TS_{7,11}}$	-3580.529628	-3580.068082	-46.9010	-2246490.031
$\mathbf{TS_{11,8}}$	-3580.535997	-3580.071837	-107.6886	-2246492.388
$\mathbf{TS_{11,12}}$	-3580.572019	-3580.108886	-122.4763	-2246515.636

**$TS_{12,8}$**	-3580.555718	-3580.098277	-568.0776	-2246508.979
**H_2**	-1.15888463	-1.16695563	-	-732.2646579
**CO_2**	-188.6189942	-188.6384702	-	-118370.6401
**H_2O**	-76.4456879	-76.4523859	-	-47969.24624
**CH_2O**	-114.5114782	-114.5176042	-	-71857.10695
**$CH_2(OH)_2$**	-190.9815598	-190.9618968	-	-119825.9005
**HCOOH**	-189.7989267	-189.8018857	-	-119097.9936
**MeOH**	-115.7246938	-115.7078068	-	-72603.95903

Cartesian coordinates for calculated structures

Mn-3

Mn 5.4614520000 4.8056280000 10.8625270000
P 7.3078890000 5.9673770000 11.3910890000
P 3.4070200000 3.9841030000 10.4246950000
O 6.4378050000 2.2793080000 12.0228050000
O 6.4688200000 4.1251030000 8.1887340000
N 4.6127140000 6.0087390000 12.2745610000
H 3.7413130000 5.7198450000 12.7042220000
C 2.3393820000 5.4444480000 10.2658070000
C 0.9985880000 5.4457800000 9.8957570000
H 0.4805120000 4.5068730000 9.7348190000
C 0.3380960000 6.6464310000 9.6844610000
H -0.6981990000 6.6489570000 9.3727260000
C 1.0219540000 7.8448650000 9.8528180000
H 0.5130990000 8.7841710000 9.6754970000
C 2.3463710000 7.8481510000 10.2611910000
H 2.8656070000 8.7868850000 10.4155140000
C 3.0249370000 6.6482190000 10.4869900000
C 4.4282850000 6.6105930000 10.9550480000
H 4.9089430000 7.5849590000 10.8890780000
C 5.4462280000 6.7106050000 13.2711600000
H 5.6270290000 6.0004300000 14.0791440000
H 4.8929930000 7.5564550000 13.6890530000
C 6.7502900000 7.1778580000 12.6638390000
H 7.5078410000 7.3446570000 13.4281330000
H 6.6180660000 8.1253690000 12.1419340000
C 3.2561540000 3.0803880000 8.8548160000
C 2.8893850000 3.7111280000 7.6685300000
H 2.5694690000 4.7462620000 7.6818970000
C 2.9365210000 3.0226740000 6.4641270000
H 2.6450220000 3.5235660000 5.5497760000
C 3.3549910000 1.7011110000 6.4308170000
H 3.3904280000 1.1655410000 5.4910080000

C	3.7317730000	1.0673950000	7.6081910000
H	4.0636700000	0.0372310000	7.5887760000
C	3.6875150000	1.7527490000	8.8108240000
H	3.9950190000	1.2569510000	9.7258470000
C	2.5365330000	2.8955530000	11.6083670000
C	1.4117460000	2.1543620000	11.2402200000
H	1.0573670000	2.1835020000	10.2163910000
C	0.7480040000	1.3732220000	12.1727830000
H	-0.1214220000	0.8019470000	11.8734200000
C	1.1984270000	1.3200800000	13.4849940000
H	0.6808900000	0.7066160000	14.2112440000
C	2.3170340000	2.0486440000	13.8608720000
H	2.6770860000	2.0054310000	14.8807210000
C	2.9834850000	2.8286420000	12.9273210000
H	3.8654110000	3.3875900000	13.2211630000
C	8.6470760000	4.9955270000	12.1387160000
C	8.7411450000	4.8116430000	13.5163790000
H	8.0765870000	5.3463650000	14.1847100000
C	9.6799170000	3.9373550000	14.0465680000
H	9.7444720000	3.8070830000	15.1193180000
C	10.5309900000	3.2337390000	13.2077920000
H	11.2625750000	2.5526160000	13.6227320000
C	10.4383380000	3.4037550000	11.8322070000
H	11.0970270000	2.8548290000	11.1713790000
C	9.5003450000	4.2733530000	11.3012080000
H	9.4237530000	4.3917990000	10.2251090000
C	8.1766510000	7.0285340000	10.1891140000
C	9.4463710000	7.5421230000	10.4583920000
H	9.9533970000	7.2721560000	11.3777770000
C	10.0642980000	8.3960000000	9.5588770000
H	11.0498000000	8.7864600000	9.7784810000
C	9.4211360000	8.7500480000	8.3802240000
H	9.9062870000	9.4146230000	7.6769140000
C	8.1571950000	8.2496950000	8.1060240000
H	7.6512410000	8.5228540000	7.1888820000
C	7.5386820000	7.3926980000	9.0049990000
H	6.5501790000	7.0021550000	8.7911620000
C	6.0459820000	3.2916680000	11.5770240000
C	6.0513370000	4.4085890000	9.2494980000

Mn-7

Mn	1.9766980000	8.3665260000	4.6183020000
P	0.9775550000	8.4347250000	6.6460840000
P	2.7748600000	8.5340310000	2.4631920000

O	4.4148540000	7.2451210000	5.7421500000
O	0.9238370000	5.6457840000	4.1851410000
N	2.0507110000	10.2908380000	4.8262570000
C	0.7660530000	10.2247870000	6.9174750000
H	-0.2133710000	10.4651300000	6.4991670000
H	0.7519370000	10.5019050000	7.9716100000
C	1.8483640000	10.9266450000	6.1308340000
H	1.5770940000	11.9809790000	5.9954740000
H	2.7852970000	10.9373560000	6.7090860000
C	2.9293810000	11.1530420000	4.0234230000
H	3.9700600000	10.7995740000	4.0868900000
H	2.9437510000	12.1626270000	4.4438450000
C	2.5487600000	11.2832820000	2.5767150000
C	2.2996950000	12.5444870000	2.0435190000
H	2.3669220000	13.4086510000	2.6941570000
C	1.9663580000	12.7082770000	0.7076840000
H	1.7702420000	13.6991090000	0.3182400000
C	1.8803850000	11.6021520000	-0.1236910000
H	1.6236690000	11.7200500000	-1.1684850000
C	2.1269830000	10.3385130000	0.3893910000
H	2.0629490000	9.4760370000	-0.2630940000
C	2.4634740000	10.1684650000	1.7315380000
C	1.9595680000	7.7695030000	8.0149450000
C	2.0764910000	6.3819890000	8.1370290000
H	1.5077310000	5.7348160000	7.4771740000
C	2.9151290000	5.8275970000	9.0882640000
H	2.9905980000	4.7514110000	9.1764910000
C	3.6623760000	6.6501980000	9.9221850000
H	4.3221840000	6.2167800000	10.6625340000
C	3.5617230000	8.0273500000	9.8008030000
H	4.1423510000	8.6736090000	10.4465060000
C	2.7148540000	8.5864700000	8.8531350000
H	2.6489830000	9.6641170000	8.7697770000
C	-0.6786720000	7.7174890000	6.8717220000
C	-1.5203330000	7.6470730000	5.7607710000
H	-1.1541170000	7.9770580000	4.7931640000
C	-2.8111520000	7.1586620000	5.8849230000
H	-3.4542750000	7.1090880000	5.0157240000
C	-3.2731050000	6.7263400000	7.1203090000
H	-4.2785350000	6.3373480000	7.2171330000
C	-2.4432990000	6.7906140000	8.2306270000
H	-2.8008000000	6.4553210000	9.1957810000
C	-1.1535890000	7.2855690000	8.1092990000
H	-0.5131870000	7.3355730000	8.9818250000
C	2.0205600000	7.4015160000	1.2490010000
C	0.6322370000	7.2536150000	1.2874090000

H	0.0603780000	7.7765060000	2.0479550000
C	-0.0173520000	6.4471480000	0.3687110000
H	-1.0940970000	6.3444290000	0.4094380000
C	0.7131340000	5.7668290000	-0.5971580000
H	0.2073710000	5.1294020000	-1.3108650000
C	2.0914890000	5.9062840000	-0.6435440000
H	2.6659080000	5.3816780000	-1.3962300000
C	2.7428260000	6.7244410000	0.2697040000
H	3.8184140000	6.8359860000	0.2130100000
C	4.5669220000	8.2830620000	2.2669510000
C	5.0947670000	7.0344250000	2.6082120000
H	4.4287570000	6.2434740000	2.9383700000
C	6.4558310000	6.7992560000	2.5247710000
H	6.8495280000	5.8244860000	2.7825880000
C	7.3136410000	7.8136600000	2.1173810000
H	8.3789630000	7.6317950000	2.0591290000
C	6.8011020000	9.0583390000	1.7874710000
H	7.4641540000	9.8526430000	1.4694460000
C	5.4340810000	9.2927050000	1.8557610000
H	5.0435800000	10.2661320000	1.5845820000
C	3.4312800000	7.6679580000	5.2655060000
C	1.3270950000	6.7377130000	4.3184440000

Mn-8

H	0.4399070000	8.5768240000	3.9144680000
Mn	1.9315290000	8.3402450000	4.5661080000
P	0.9423400000	8.4309720000	6.5702620000
P	2.6732660000	8.5537940000	2.4415010000
O	4.6063590000	8.0311450000	5.7725210000
O	1.6594310000	5.4352900000	4.3272410000
N	1.9392230000	10.5232200000	4.7836410000
H	1.0342670000	10.8278510000	4.4345030000
C	0.8780940000	10.2232610000	6.9604420000
H	-0.0858220000	10.5874560000	6.5985040000
H	0.9220410000	10.4289320000	8.0293440000
C	1.9989900000	10.9048110000	6.2090480000
H	1.9327430000	11.9926470000	6.3117840000
H	2.9764570000	10.6003390000	6.5907410000
C	2.9738000000	11.2285720000	3.9931430000
H	3.9175490000	10.6907190000	4.1269150000
H	3.1200310000	12.2361960000	4.3924970000
C	2.5875450000	11.3409940000	2.5483820000
C	2.3869720000	12.6080610000	2.0100720000
H	2.5315410000	13.4721570000	2.6476090000

C	2.0058420000	12.7784140000	0.6883610000
H	1.8545860000	13.7746280000	0.2935300000
C	1.8117700000	11.6683920000	-0.1163540000
H	1.5082530000	11.7856330000	-1.1486210000
C	2.0109490000	10.3980570000	0.4026940000
H	1.8593860000	9.5380820000	-0.2369400000
C	2.4054420000	10.2147660000	1.7266610000
C	1.8689530000	7.6588520000	7.9364210000
C	1.8209080000	6.2683970000	8.0637880000
H	1.1690640000	5.6919000000	7.4153250000
C	2.5968450000	5.6179210000	9.0086020000
H	2.5444230000	4.5402920000	9.0975790000
C	3.4396140000	6.3460630000	9.8387170000
H	4.0472580000	5.8386680000	10.5767220000
C	3.4982530000	7.7258530000	9.7172800000
H	4.1521590000	8.3004890000	10.3609230000
C	2.7202810000	8.3797830000	8.7712820000
H	2.7869530000	9.4576510000	8.6878140000
C	-0.7439800000	7.8151040000	6.8858420000
C	-1.4141490000	7.0566850000	5.9300510000
H	-0.9342650000	6.8500320000	4.9802190000
C	-2.6896410000	6.5711340000	6.1852900000
H	-3.1996200000	5.9796220000	5.4358040000
C	-3.3076920000	6.8448320000	7.3954730000
H	-4.3034190000	6.4692790000	7.5934850000
C	-2.6467150000	7.6002010000	8.3561390000
H	-3.1263030000	7.8149020000	9.3025940000
C	-1.3705750000	8.0765370000	8.1062680000
H	-0.8542270000	8.6547600000	8.8646400000
C	1.9974530000	7.4133060000	1.1801370000
C	0.7150780000	6.8880890000	1.3334780000
H	0.1374010000	7.1457990000	2.2131900000
C	0.1801930000	6.0381220000	0.3769620000
H	-0.8163810000	5.6378490000	0.5126910000
C	0.9216110000	5.6958050000	-0.7440610000
H	0.5066000000	5.0270900000	-1.4872080000
C	2.1998520000	6.2107890000	-0.9075660000
H	2.7843260000	5.9494090000	-1.7803940000
C	2.7336140000	7.0633920000	0.0459030000
H	3.7302130000	7.4645810000	-0.0954210000
C	4.4743030000	8.3024590000	2.2373030000
C	4.9921310000	7.0479540000	2.5720990000
H	4.3200780000	6.2595040000	2.8960610000
C	6.3518630000	6.8025870000	2.4996320000
H	6.7356830000	5.8233420000	2.7559800000
C	7.2222880000	7.8124580000	2.1063530000

H	8.2867150000	7.6230330000	2.0562690000
C	6.7206040000	9.0621490000	1.7808010000
H	7.3913440000	9.8541540000	1.4728440000
C	5.3536700000	9.3056180000	1.8399100000
H	4.9761020000	10.2851130000	1.5720340000
C	3.5513750000	8.1844240000	5.2890720000
C	1.7669480000	6.5995570000	4.4175080000

Mn-9

Mn	1.9096690000	8.1780180000	4.5085030000
P	0.9880490000	8.3692030000	6.6055300000
P	2.8439260000	8.3274040000	2.3968330000
O	4.5116220000	7.6397900000	5.7465050000
O	1.5048760000	5.2846700000	4.2373190000
N	2.1747950000	10.3257750000	4.7399160000
H	1.2829660000	10.5958240000	4.3222590000
C	0.9713830000	10.1771400000	6.8555750000
H	0.0556520000	10.5415250000	6.3819970000
H	0.9350470000	10.4766900000	7.9021730000
C	2.1736470000	10.7611600000	6.1475320000
H	2.1612860000	11.8539370000	6.2052940000
H	3.1083210000	10.4257150000	6.6044510000
C	3.2700100000	10.9605230000	3.9723910000
H	4.1754320000	10.3629750000	4.1185220000
H	3.4787570000	11.9527630000	4.3810380000
C	2.9154960000	11.1075750000	2.5222690000
C	2.7894610000	12.3870660000	1.9917180000
H	2.9739360000	13.2376830000	2.6369650000
C	2.4304010000	12.5863790000	0.6676740000
H	2.3374160000	13.5920740000	0.2789280000
C	2.1815670000	11.4946880000	-0.1469630000
H	1.8925690000	11.6360760000	-1.1803060000
C	2.3059390000	10.2115490000	0.3638330000
H	2.1109730000	9.3648380000	-0.2817300000
C	2.6812290000	9.9998010000	1.6888960000
C	2.0345270000	7.6802690000	7.9313130000
C	2.2476360000	6.2994670000	7.9420540000
H	1.7659350000	5.6742500000	7.1978770000
C	3.0795610000	5.7203640000	8.8846560000
H	3.2311080000	4.6485880000	8.8811220000
C	3.7259000000	6.5137770000	9.8239560000
H	4.3832520000	6.0630930000	10.5561430000
C	3.5283500000	7.8852850000	9.8160280000
H	4.0301440000	8.5110620000	10.5429890000

C	2.6855140000	8.4671680000	8.8777810000
H	2.5462050000	9.5404520000	8.8916710000
C	-0.6667030000	7.7888040000	7.0964840000
C	-1.5349960000	8.5840720000	7.8433020000
H	-1.2366240000	9.5782990000	8.1510700000
C	-2.7898770000	8.1107860000	8.1971410000
H	-3.4565970000	8.7399060000	8.7729270000
C	-3.1881520000	6.8372610000	7.8180810000
H	-4.1684730000	6.4706270000	8.0937210000
C	-2.3262090000	6.0348040000	7.0831320000
H	-2.6316970000	5.0407210000	6.7829370000
C	-1.0757320000	6.5082680000	6.7203090000
H	-0.4164180000	5.8834130000	6.1289490000
C	2.1901920000	7.2186100000	1.1030730000
C	0.8885160000	6.7294520000	1.2034710000
H	0.2823520000	7.0038160000	2.0573180000
C	0.3719290000	5.8978580000	0.2211380000
H	-0.6395790000	5.5235190000	0.3132780000
C	1.1495930000	5.5422640000	-0.8711290000
H	0.7472030000	4.8893220000	-1.6349370000
C	2.4470250000	6.0226600000	-0.9800730000
H	3.0591640000	5.7490670000	-1.8298510000
C	2.9645300000	6.8556510000	-0.0007720000
H	3.9770620000	7.2298780000	-0.0962400000
C	4.6340760000	7.9867640000	2.3160280000
C	5.0594800000	6.7092800000	2.6901920000
H	4.3267720000	5.9664070000	2.9892850000
C	6.4048560000	6.3862080000	2.6880790000
H	6.7192000000	5.3911130000	2.9756320000
C	7.3488750000	7.3390420000	2.3244790000
H	8.4017840000	7.0885960000	2.3282300000
C	6.9374660000	8.6106610000	1.9586490000
H	7.6672630000	9.3572970000	1.6729430000
C	5.5863860000	8.9333990000	1.9491900000
H	5.2772740000	9.9283150000	1.6518780000
C	3.4727430000	7.8475130000	5.2487460000
C	1.6297960000	6.4434810000	4.3382160000
O	0.2142540000	8.9158760000	3.5786900000
C	-1.0404730000	8.3649720000	3.7717120000
H	-1.7210280000	8.6222630000	2.9433470000
H	-1.0389180000	7.2619970000	3.8354370000
H	-1.5384700000	8.7152310000	4.6939740000

Mn-10

Mn	1.7168480000	8.2890550000	4.5356980000
P	0.8502090000	8.3674980000	6.6559950000
P	2.5706880000	8.5204330000	2.4032900000
O	4.3881640000	7.9346830000	5.6742850000
O	1.6637700000	5.3630750000	4.3363890000
N	1.8465130000	10.4603940000	4.8201090000
H	0.9443200000	10.8078450000	4.4990110000
C	0.8196300000	10.1606510000	7.0141530000
H	-0.1392100000	10.5313210000	6.6427360000
H	0.8663720000	10.3887930000	8.0783350000
C	1.9490380000	10.8154800000	6.2509930000
H	1.9189290000	11.9024880000	6.3709270000
H	2.9240750000	10.4783010000	6.6104570000
C	2.8935540000	11.1447820000	4.0216250000
H	3.8208310000	10.5750760000	4.1325760000
H	3.0808010000	12.1386090000	4.4366430000
C	2.4814470000	11.2965330000	2.5871520000
C	2.2209420000	12.5736000000	2.1032800000
H	2.3656120000	13.4189810000	2.7652420000
C	1.7705090000	12.7765880000	0.8075990000
H	1.5686330000	13.7803160000	0.4571890000
C	1.5699420000	11.6900320000	-0.0263060000
H	1.2100990000	11.8328660000	-1.0368570000
C	1.8349840000	10.4090270000	0.4348460000
H	1.6731290000	9.5667650000	-0.2257330000
C	2.2985590000	10.1938100000	1.7314770000
C	1.8787690000	7.6038060000	7.9466880000
C	1.9252690000	6.2085540000	8.0014380000
H	1.2982000000	5.6224610000	7.3378210000
C	2.7645120000	5.5658870000	8.8953920000
H	2.7858890000	4.4842120000	8.9296990000
C	3.5782580000	6.3073650000	9.7425400000
H	4.2379050000	5.8057000000	10.4385920000
C	3.5422280000	7.6919820000	9.6923770000
H	4.1734270000	8.2762790000	10.3496320000
C	2.6980540000	8.3392510000	8.7999920000
H	2.6892650000	9.4215930000	8.7737310000
C	-0.8155440000	7.7538270000	7.0511510000
C	-1.3582390000	6.7024650000	6.3139730000
H	-0.8014110000	6.2872320000	5.4820390000
C	-2.6069880000	6.1912230000	6.6340070000
H	-3.0184970000	5.3754710000	6.0538320000
C	-3.3275720000	6.7288970000	7.6900630000
H	-4.3041700000	6.3331120000	7.9374570000
C	-2.7958200000	7.7768740000	8.4291670000
H	-3.3549290000	8.1983810000	9.2546580000

C	-1.5452260000	8.2853690000	8.1152040000
H	-1.1342560000	9.0972440000	8.7037420000
C	1.9783900000	7.3782530000	1.1088580000
C	0.7904020000	6.6715390000	1.2889000000
H	0.2167660000	6.8126760000	2.1971770000
C	0.3363500000	5.7972710000	0.3114170000
H	-0.5871750000	5.2543180000	0.4655040000
C	1.0668120000	5.6154180000	-0.8526960000
H	0.7149310000	4.9294390000	-1.6124230000
C	2.2539780000	6.3110810000	-1.0406640000
H	2.8285560000	6.1712820000	-1.9471900000
C	2.7080090000	7.1848470000	-0.0671270000
H	3.6361530000	7.7237050000	-0.2199540000
C	4.3776340000	8.2823620000	2.2909970000
C	4.8792810000	7.0125200000	2.5882900000
H	4.1954320000	6.2109800000	2.8481440000
C	6.2411150000	6.7696000000	2.5587660000
H	6.6149680000	5.7796790000	2.7866390000
C	7.1249410000	7.7944160000	2.2431000000
H	8.1904340000	7.6054940000	2.2247510000
C	6.6368310000	9.0580430000	1.9518760000
H	7.3187000000	9.8607610000	1.7021890000
C	5.2693210000	9.3014930000	1.9705930000
H	4.9005900000	10.2915910000	1.7307890000
C	3.3253280000	8.0842190000	5.2101020000
C	1.6372370000	6.5295350000	4.3955010000
O	-0.2784170000	8.6630570000	3.9194670000
C	-0.7961650000	9.6418060000	3.1574530000
H	-1.7296190000	10.0460310000	3.5870000000
H	-0.1211660000	10.5033780000	2.9877880000
O	-1.2295130000	9.2035990000	1.8402780000
H	-0.4328330000	9.0379340000	1.3184010000

Mn-11

Mn	1.8305570000	8.2841690000	4.5121940000
P	0.9466210000	8.3944880000	6.6076230000
P	2.6824100000	8.4920700000	2.3598090000
O	4.4890140000	7.9452370000	5.6817940000
O	1.7419010000	5.3534100000	4.4187670000
N	1.9286790000	10.4614900000	4.7742840000
H	0.9980670000	10.8053720000	4.5405090000
C	1.0973350000	10.1678770000	7.0594280000
H	0.1290010000	10.6275410000	6.8485280000
H	1.3012080000	10.3154380000	8.1192370000

C	2.1623940000	10.8060020000	6.1948660000
H	2.1576240000	11.8924600000	6.3213760000
H	3.1607700000	10.4546470000	6.4624370000
C	2.8811310000	11.1727550000	3.8867600000
H	3.8416600000	10.6536370000	3.9501230000
H	3.0422350000	12.1872270000	4.2605600000
C	2.3627200000	11.2513080000	2.4819390000
C	1.9670350000	12.4873230000	1.9834220000
H	2.0754930000	13.3612790000	2.6146030000
C	1.4368820000	12.6128940000	0.7084120000
H	1.1326240000	13.5850640000	0.3432910000
C	1.2911450000	11.4891400000	-0.0872540000
H	0.8730340000	11.5725240000	-1.0819090000
C	1.6851770000	10.2484990000	0.3914040000
H	1.5661180000	9.3759150000	-0.2379100000
C	2.2299660000	10.1139280000	1.6663330000
C	1.8471380000	7.4630330000	7.8821520000
C	1.5652590000	6.1077020000	8.0616260000
H	0.7646230000	5.6434390000	7.4962570000
C	2.2992900000	5.3506450000	8.9601670000
H	2.0657450000	4.3022690000	9.0941960000
C	3.3282480000	5.9346760000	9.6864460000
H	3.9004750000	5.3431510000	10.3892300000
C	3.6206550000	7.2784010000	9.5075390000
H	4.4229680000	7.7398640000	10.0687530000
C	2.8876660000	8.0395560000	8.6086790000
H	3.1360360000	9.0851720000	8.4729350000
C	-0.7880120000	7.9453400000	6.9137230000
C	-1.4169370000	7.0230440000	6.0782280000
H	-0.8712230000	6.5928970000	5.2459110000
C	-2.7384140000	6.6645510000	6.2983620000
H	-3.2177190000	5.9494780000	5.6424080000
C	-3.4439970000	7.2275390000	7.3513940000
H	-4.4775870000	6.9535620000	7.5190010000
C	-2.8245230000	8.1445860000	8.1900150000
H	-3.3722550000	8.5837590000	9.0138280000
C	-1.5023800000	8.5002200000	7.9760650000
H	-1.0243000000	9.2122130000	8.6386100000
C	2.1481700000	7.2803580000	1.1057820000
C	0.9644160000	6.5644690000	1.2769650000
H	0.3620510000	6.7287420000	2.1612920000
C	0.5537120000	5.6483590000	0.3197190000
H	-0.3662280000	5.0972840000	0.4663350000
C	1.3198220000	5.4358840000	-0.8165080000
H	1.0000940000	4.7172580000	-1.5600800000
C	2.4993230000	6.1450300000	-0.9969800000

H	3.1012750000	5.9842220000	-1.8820190000
C	2.9115390000	7.0611350000	-0.0431190000
H	3.8331390000	7.6118180000	-0.1916320000
C	4.4962700000	8.4021460000	2.2188270000
C	5.1088700000	7.2097160000	2.6124120000
H	4.5003690000	6.3879890000	2.9775180000
C	6.4836790000	7.0700240000	2.5414140000
H	6.9457030000	6.1394610000	2.8449080000
C	7.2675930000	8.1236220000	2.0873480000
H	8.3432900000	8.0159230000	2.0365720000
C	6.6685730000	9.3120000000	1.7006470000
H	7.2738430000	10.1354820000	1.3441850000
C	5.2881870000	9.4510590000	1.7607770000
H	4.8293550000	10.3806890000	1.4457560000
C	3.4301830000	8.0911500000	5.2137670000
C	1.7362980000	6.5194970000	4.4262350000
O	-2.0318710000	9.5458170000	3.2314000000
C	-0.9934330000	9.3759860000	3.8842450000
H	-0.8984140000	9.9103910000	4.8578850000
O	-0.0172130000	8.6442780000	3.5365360000

Mn-12

Mn	1.9969010000	8.3040630000	4.6008170000
P	1.0469110000	8.4102380000	6.6650750000
P	2.7886430000	8.4990420000	2.4372430000
O	4.6448350000	7.9407700000	5.7622240000
O	1.8123660000	5.3744350000	4.4854740000
N	2.0431740000	10.4427050000	4.8335510000
C	1.1317940000	10.1925040000	7.0892960000
H	0.1605550000	10.6190520000	6.8309470000
H	1.2909630000	10.3623410000	8.1532750000
C	2.2142850000	10.8343690000	6.2504510000
H	2.1718400000	11.9236960000	6.3400070000
H	3.2102760000	10.5223730000	6.5715280000
C	2.9881790000	11.1781040000	3.9558550000
H	3.9618910000	10.6836180000	4.0156500000
H	3.1230790000	12.1928560000	4.3382600000
C	2.4673290000	11.2570660000	2.5525750000
C	2.0547180000	12.4885480000	2.0572310000
H	2.1634140000	13.3646860000	2.6852500000
C	1.5004650000	12.6050910000	0.7919430000
H	1.1806150000	13.5733460000	0.4297420000
C	1.3479960000	11.4775690000	0.0019380000
H	0.9104730000	11.5551140000	-0.9847420000

C	1.7606910000	10.2418030000	0.4761980000
H	1.6376910000	9.3659910000	-0.1481150000
C	2.3292210000	10.1170930000	1.7423790000
C	1.9173840000	7.5136910000	7.9800270000
C	1.6351260000	6.1621000000	8.1851210000
H	0.8505310000	5.6818830000	7.6105810000
C	2.3485200000	5.4317360000	9.1216330000
H	2.1155390000	4.3862050000	9.2768680000
C	3.3559930000	6.0390640000	9.8587870000
H	3.9113090000	5.4683180000	10.5916590000
C	3.6493220000	7.3788770000	9.6528550000
H	4.4352620000	7.8573550000	10.2228130000
C	2.9376720000	8.1137180000	8.7160590000
H	3.1843550000	9.1568020000	8.5587810000
C	-0.6873900000	7.9205240000	6.8828430000
C	-1.3314940000	7.1787570000	5.8954910000
H	-0.7937530000	6.8991400000	4.9962440000
C	-2.6578860000	6.8028690000	6.0540260000
H	-3.1499990000	6.2295630000	5.2790010000
C	-3.3482030000	7.1649190000	7.2002090000
H	-4.3837640000	6.8750600000	7.3231500000
C	-2.7113270000	7.8996810000	8.1926980000
H	-3.2488010000	8.1812800000	9.0889840000
C	-1.3870900000	8.2737000000	8.0383350000
H	-0.8926370000	8.8425510000	8.8174760000
C	2.1586000000	7.2881150000	1.2354490000
C	0.9014460000	6.7211350000	1.4402870000
H	0.3329000000	6.9776400000	2.3269820000
C	0.3725750000	5.8329980000	0.5167420000
H	-0.6050170000	5.4006980000	0.6867070000
C	1.0983390000	5.4954170000	-0.6163020000
H	0.6886700000	4.7968890000	-1.3344430000
C	2.3527240000	6.0511850000	-0.8264220000
H	2.9221970000	5.7901770000	-1.7090330000
C	2.8805260000	6.9452660000	0.0916720000
H	3.8566000000	7.3825010000	-0.0827810000
C	4.5932030000	8.3728800000	2.2506470000
C	5.1968600000	7.1752720000	2.6422380000
H	4.5852010000	6.3660820000	3.0291960000
C	6.5676400000	7.0163410000	2.5403520000
H	7.0240800000	6.0822790000	2.8413350000
C	7.3547530000	8.0555540000	2.0595150000
H	8.4274680000	7.9327470000	1.9855790000
C	6.7637850000	9.2491210000	1.6762290000
H	7.3722860000	10.0612680000	1.2999650000
C	5.3873440000	9.4080850000	1.7661570000

H	4.9333690000	10.3410740000	1.4544940000
C	3.5881650000	8.0877770000	5.2965340000
C	1.8526040000	6.5376150000	4.5035030000
H	0.2390760000	8.7472380000	3.7607990000
C	-0.6901550000	9.3815860000	3.4491030000
O	-1.5003840000	8.7480170000	2.7690520000
O	-0.6853190000	10.5521340000	3.8556580000
H	1.0910750000	10.7196740000	4.5569030000

$TS_{7,8}$

Mn	1.9271710000	8.3718450000	4.5737610000
P	0.9659620000	8.4372540000	6.6123350000
P	2.7309940000	8.5737750000	2.4416310000
O	4.5742200000	7.9608610000	5.7777620000
O	1.4843020000	5.4832510000	4.2652060000
N	1.9146350000	10.5168720000	4.7923330000
C	0.8513940000	10.2263880000	6.9657420000
H	-0.1214060000	10.5506940000	6.5908140000
H	0.8855430000	10.4508830000	8.0315330000
C	1.9625570000	10.9017780000	6.1882960000
H	1.8658660000	11.9931300000	6.2826470000
H	2.9359070000	10.6457290000	6.6455370000
C	2.9214460000	11.2171530000	4.0125840000
H	3.9206030000	10.7527250000	4.1172770000
H	3.0307090000	12.2348690000	4.4075110000
C	2.5631350000	11.3409600000	2.5582750000
C	2.3290390000	12.6018640000	2.0178140000
H	2.4131180000	13.4677230000	2.6641690000
C	1.9935780000	12.7664680000	0.6826470000
H	1.8162070000	13.7590850000	0.2888800000
C	1.8829360000	11.6579550000	-0.1413210000
H	1.6209940000	11.7735220000	-1.1850740000
C	2.1111760000	10.3930400000	0.3775930000
H	2.0228350000	9.5297670000	-0.2704320000
C	2.4556440000	10.2208990000	1.7174750000
C	1.9257460000	7.6887310000	7.9600500000
C	1.9481300000	6.2953720000	8.0595320000
H	1.3322970000	5.6988200000	7.3945000000
C	2.7490470000	5.6694460000	8.9995130000
H	2.7522200000	4.5892250000	9.0691060000
C	3.5469690000	6.4256390000	9.8489350000
H	4.1750450000	5.9366600000	10.5822430000
C	3.5358600000	7.8084800000	9.7531450000
H	4.1552530000	8.4037510000	10.4117980000

C	2.7311750000	8.4389050000	8.8135460000
H	2.7407200000	9.5199090000	8.7473190000
C	-0.7063010000	7.7755500000	6.8858870000
C	-1.4361200000	7.2341240000	5.8305130000
H	-0.9961630000	7.1938220000	4.8403460000
C	-2.7204830000	6.7508700000	6.0407180000
H	-3.2780900000	6.3295840000	5.2141950000
C	-3.2847580000	6.8051270000	7.3056510000
H	-4.2854200000	6.4263730000	7.4695360000
C	-2.5642370000	7.3438170000	8.3644340000
H	-3.0024560000	7.3878310000	9.3532290000
C	-1.2820650000	7.8238150000	8.1573280000
H	-0.7209740000	8.2388100000	8.9874260000
C	2.0056840000	7.4344110000	1.2144790000
C	0.6561740000	7.0982460000	1.3293440000
H	0.0768880000	7.4911380000	2.1580980000
C	0.0558160000	6.2638760000	0.4000910000
H	-0.9919000000	6.0119850000	0.5022740000
C	0.7985160000	5.7459600000	-0.6519030000
H	0.3321580000	5.0871520000	-1.3730170000
C	2.1409860000	6.0722480000	-0.7745130000
H	2.7250980000	5.6729290000	-1.5936800000
C	2.7412150000	6.9145610000	0.1497940000
H	3.7878410000	7.1707530000	0.0384710000
C	4.5228290000	8.2935840000	2.2674300000
C	5.0250010000	7.0416150000	2.6327140000
H	4.3430220000	6.2685250000	2.9728580000
C	6.3826530000	6.7826170000	2.5691940000
H	6.7573500000	5.8063300000	2.8487290000
C	7.2622660000	7.7759690000	2.1555140000
H	8.3249970000	7.5756040000	2.1126790000
C	6.7744790000	9.0235560000	1.8010370000
H	7.4542530000	9.8018430000	1.4787100000
C	5.4104130000	9.2817190000	1.8511750000
H	5.0394190000	10.2581360000	1.5634680000
C	3.5262430000	8.1063310000	5.2884700000
C	1.6476470000	6.6355660000	4.3739690000
H	0.4038240000	8.9409410000	3.9334500000
H	0.8458210000	9.7225770000	4.2241750000

$TS_{8,9}$

Mn	-0.4274500000	-0.5008000000	-0.3251400000
P	1.8936470000	-0.8768870000	-0.0616000000
P	-2.3854880000	0.6763440000	-0.3402970000

O	-1.4667060000	-3.2113650000	-0.0428150000
O	-0.7018480000	-0.5450020000	2.5690240000
N	0.3687480000	2.7498120000	0.4225690000
H	0.2759480000	2.2398400000	-0.4494130000
C	2.8714410000	0.6257380000	-0.4548850000
C	3.8613640000	0.5576120000	-1.4378910000
H	4.0548500000	-0.3803420000	-1.9405870000
C	4.6073360000	1.6695970000	-1.7946370000
H	5.3671320000	1.5820320000	-2.5603080000
C	4.3796730000	2.8795100000	-1.1617220000
H	4.9645330000	3.7531880000	-1.4187480000
C	3.3873870000	2.9663730000	-0.1999220000
H	3.1943060000	3.9133180000	0.2907930000
C	2.6087660000	1.8686000000	0.1590210000
C	1.4837730000	2.1139310000	1.1300350000
H	1.1810860000	1.1825170000	1.6237900000
H	1.8341850000	2.7702700000	1.9322860000
C	-0.9255190000	2.6901860000	1.1061660000
H	-1.1218830000	3.6393670000	1.6136360000
H	-0.9257700000	1.9345260000	1.8992370000
C	-2.0618950000	2.4351550000	0.1178310000
H	-1.8479730000	2.9574320000	-0.8171590000
H	-2.9958710000	2.8568120000	0.4920390000
C	-1.0461970000	-2.1252700000	-0.1355550000
C	-0.5654810000	-0.4803610000	1.4077440000
H	-0.2089280000	-0.9829510000	-2.4726710000
C	-3.6411770000	0.1080780000	0.8509470000
H	-3.2901500000	1.6228850000	2.3431190000
C	-4.7324620000	0.1912020000	3.0029660000
H	-4.8748260000	0.6818130000	3.9573350000
C	-5.4402020000	-0.9615710000	2.7003180000
H	-6.1383400000	-1.3745710000	3.4168300000
C	-5.2485440000	-1.5855680000	1.4744140000
H	-5.7974430000	-2.4861230000	1.2306610000
C	-4.3523840000	-1.0585050000	0.5599750000
H	-4.2029290000	-1.5567580000	-0.3920860000
C	-3.8375450000	0.7246220000	2.0854060000
C	-3.3121730000	0.7790560000	-1.9018620000
H	-4.2636600000	2.6660510000	-1.4853250000
C	-4.8694990000	1.8781090000	-3.3793750000
H	-5.5203310000	2.7154050000	-3.5963550000
C	-4.7515340000	0.8345090000	-4.2875420000
H	-5.3093160000	0.8585180000	-5.2148180000
C	-3.9190060000	-0.2384000000	-4.0055000000
H	-3.8250490000	-1.0546700000	-4.7100430000
C	-3.2006690000	-0.2647160000	-2.8192920000

H	-2.5462270000	-1.1008460000	-2.5954610000
C	-4.1575920000	1.8509300000	-2.1907900000
C	2.3687390000	-1.3116940000	1.6493900000
H	3.6506950000	0.3685110000	2.0509420000
C	3.5113590000	-0.9372500000	3.7402840000
H	4.1692190000	-0.3276640000	4.3463060000
C	2.9737750000	-2.1060960000	4.2550170000
H	3.2066070000	-2.4120250000	5.2666970000
C	2.1358290000	-2.8851590000	3.4668560000
H	1.7134510000	-3.8004160000	3.8610740000
C	1.8333060000	-2.4896260000	2.1757620000
H	1.1688880000	-3.0959390000	1.5682010000
C	3.2143210000	-0.5420650000	2.4423550000
C	2.6970840000	-2.2528020000	-0.9553150000
H	0.9381990000	-2.7403300000	-2.0839660000
C	2.5490010000	-4.0881400000	-2.5160980000
H	1.9706250000	-4.6635200000	-3.2272260000
C	3.8616280000	-4.4359500000	-2.2406580000
H	4.3143850000	-5.2834270000	-2.7389580000
C	4.5946130000	-3.7008690000	-1.3166880000
H	5.6181200000	-3.9736560000	-1.0941180000
C	4.0156080000	-2.6209430000	-0.6737760000
H	4.5887200000	-2.0556110000	0.0527480000
C	1.9685500000	-3.0016710000	-1.8753170000
O	0.0536570000	0.9436170000	-1.6889140000
C	0.2619700000	0.0091300000	-2.6918120000
H	1.3288800000	-0.1995590000	-2.8833080000
H	-0.2050480000	0.3033910000	-3.6420930000

$TS_{9,7}$

Mn	2.0749750000	8.2592600000	4.5873840000
P	1.2597170000	8.4424360000	6.7152200000
P	2.8303830000	8.3829120000	2.4105790000
O	4.7792670000	7.7831600000	5.5613870000
O	1.4756130000	5.3850520000	4.3964500000
N	2.3455010000	10.3533820000	4.8033150000
H	1.0211670000	10.1373860000	4.3313060000
C	1.3473060000	10.2409250000	7.0210310000
H	0.4134560000	10.6825950000	6.6724920000
H	1.4502140000	10.4849630000	8.0768280000
C	2.4953890000	10.7781320000	6.1900040000
H	2.5076390000	11.8746130000	6.2527340000
H	3.4558050000	10.4444970000	6.6150480000
C	3.3394970000	11.0136780000	3.9593220000

H	4.3113840000	10.4944520000	4.0077330000
H	3.5318390000	12.0228140000	4.3423730000
C	2.9005880000	11.1569110000	2.5287750000
C	2.7108560000	12.4336620000	2.0068090000
H	2.8891270000	13.2877080000	2.6497480000
C	2.3007370000	12.6290310000	0.6971900000
H	2.1568900000	13.6334690000	0.3202500000
C	2.0727720000	11.5349160000	-0.1219060000
H	1.7523640000	11.6726870000	-1.1464540000
C	2.2548430000	10.2553780000	0.3777840000
H	2.0644550000	9.4056060000	-0.2653350000
C	2.6697450000	10.0505950000	1.6934470000
C	2.1536080000	7.6359010000	8.0831830000
C	2.8489780000	6.4490090000	7.8524570000
H	2.8826350000	6.0319610000	6.8532100000
C	3.4929970000	5.7937610000	8.8913060000
H	4.0271860000	4.8730310000	8.6955280000
C	3.4550490000	6.3196390000	10.1738220000
H	3.9634950000	5.8129300000	10.9839000000
C	2.7606010000	7.4965870000	10.4166990000
H	2.7222740000	7.9086180000	11.4168830000
C	2.1089470000	8.1469590000	9.3809690000
H	1.5582090000	9.0558860000	9.5896340000
C	-0.4589900000	7.9141160000	7.0079890000
C	-1.5469350000	8.7812730000	6.9104680000
H	-1.3922130000	9.8358480000	6.7244150000
C	-2.8424150000	8.3040520000	7.0515010000
H	-3.6747590000	8.9923970000	6.9792740000
C	-3.0713330000	6.9566210000	7.2840520000
H	-4.0828200000	6.5874920000	7.3933580000
C	-1.9960400000	6.0833160000	7.3759870000
H	-2.1644500000	5.0294190000	7.5566110000
C	-0.7022650000	6.5569120000	7.2371520000
H	0.1291990000	5.8641840000	7.3079600000
C	2.0629210000	7.2735620000	1.1806270000
C	0.7723470000	6.7933770000	1.3971010000
H	0.2464760000	7.0630390000	2.3052700000
C	0.1621960000	5.9641150000	0.4677880000
H	-0.8394590000	5.5974510000	0.6514060000
C	0.8381240000	5.5992040000	-0.6869120000
H	0.3653270000	4.9462590000	-1.4092030000
C	2.1252160000	6.0682370000	-0.9119030000
H	2.6578180000	5.7856550000	-1.8108510000
C	2.7336030000	6.9001220000	0.0141380000
H	3.7373840000	7.2649290000	-0.1702540000
C	4.6000990000	7.9819990000	2.2301920000

C	4.9996650000	6.6735750000	2.5148510000
H	4.2557050000	5.9335340000	2.7927570000
C	6.3350640000	6.3170560000	2.4522250000
H	6.6301780000	5.2985370000	2.6695060000
C	7.2941310000	7.2657110000	2.1177000000
H	8.3393400000	6.9882990000	2.0740350000
C	6.9075640000	8.5672540000	1.8407810000
H	7.6491190000	9.3106250000	1.5777930000
C	5.5660160000	8.9246960000	1.8917730000
H	5.2741680000	9.9427270000	1.6631280000
C	3.6878460000	7.9261180000	5.1681570000
C	1.6741340000	6.5370920000	4.4487040000
O	0.2152520000	9.3870190000	4.1440220000
C	-0.5407840000	9.5937370000	2.9743780000
H	-1.0055530000	10.5847620000	2.9898910000
H	0.0520690000	9.5173970000	2.0563820000
H	-1.3408980000	8.8522990000	2.9175370000

$TS_{7,10}$

Mn	2.1247870000	8.2314860000	4.6108530000
P	1.3075730000	8.4324990000	6.7353540000
P	2.8526150000	8.3442010000	2.4274580000
O	4.8253840000	7.7554960000	5.5853880000
O	1.5293150000	5.3524170000	4.4796850000
N	2.4123150000	10.3143290000	4.8344680000
H	1.0109900000	10.1051710000	4.3727000000
C	1.4780880000	10.2208570000	7.0739500000
H	0.5534890000	10.7103800000	6.7666300000
H	1.6255280000	10.4313200000	8.1318890000
C	2.6204690000	10.7276340000	6.2154780000
H	2.6661680000	11.8230520000	6.2821730000
H	3.5817600000	10.3651010000	6.6151460000
C	3.3703910000	10.9727100000	3.9485670000
H	4.3435720000	10.4529180000	3.9464000000
H	3.5824430000	11.9820330000	4.3211250000
C	2.8479730000	11.1129790000	2.5454820000
C	2.5807400000	12.3836930000	2.0451020000
H	2.7813150000	13.2420600000	2.6756790000
C	2.0623920000	12.5677680000	0.7720820000
H	1.8596240000	13.5679340000	0.4111400000
C	1.8007650000	11.4686550000	-0.0297850000
H	1.3940640000	11.5986770000	-1.0242880000
C	2.0619990000	10.1938320000	0.4477260000
H	1.8484610000	9.3392740000	-0.1818170000

C	2.5887460000	10.0014640000	1.7241310000
C	2.1415890000	7.5674760000	8.1050980000
C	2.8572410000	6.3951570000	7.8667720000
H	2.9452260000	6.0138480000	6.8571070000
C	3.4528950000	5.7062890000	8.9132950000
H	4.0049610000	4.7974820000	8.7115370000
C	3.3421120000	6.1821040000	10.2106910000
H	3.8118740000	5.6485230000	11.0268480000
C	2.6235380000	7.3432760000	10.4612370000
H	2.5274650000	7.7155160000	11.4730600000
C	2.0223710000	8.0277960000	9.4176460000
H	1.4510800000	8.9237580000	9.6291620000
C	-0.4376190000	7.9787700000	6.9964940000
C	-1.4870440000	8.8838970000	6.8397810000
H	-1.2857280000	9.9261940000	6.6302330000
C	-2.8044050000	8.4596520000	6.9400560000
H	-3.6059940000	9.1772610000	6.8190060000
C	-3.0939300000	7.1277330000	7.1937000000
H	-4.1222970000	6.7996670000	7.2720690000
C	-2.0573370000	6.2165920000	7.3452340000
H	-2.2730570000	5.1742050000	7.5416180000
C	-0.7417450000	6.6371250000	7.2443800000
H	0.0595010000	5.9149660000	7.3572450000
C	2.1107910000	7.2031210000	1.2113390000
C	0.8431490000	6.6717290000	1.4427410000
H	0.3185090000	6.9159480000	2.3589180000
C	0.2523760000	5.8245490000	0.5170110000
H	-0.7316940000	5.4185300000	0.7123310000
C	0.9252980000	5.4927240000	-0.6492290000
H	0.4679820000	4.8260250000	-1.3688870000
C	2.1894820000	6.0135870000	-0.8896440000
H	2.7194910000	5.7577720000	-1.7980830000
C	2.7781920000	6.8635990000	0.0326690000
H	3.7634270000	7.2698830000	-0.1646560000
C	4.6340950000	8.0241710000	2.2077530000
C	5.1042420000	6.7487040000	2.5308510000
H	4.4072830000	5.9908090000	2.8750240000
C	6.4502010000	6.4470070000	2.4216940000
H	6.8002020000	5.4533520000	2.6702370000
C	7.3495280000	7.4192590000	2.0006980000
H	8.4031570000	7.1851390000	1.9206720000
C	6.8930200000	8.6889220000	1.6846760000
H	7.5878830000	9.4502380000	1.3541710000
C	5.5406180000	8.9906880000	1.7825510000
H	5.1932210000	9.9833770000	1.5223910000
C	3.7334150000	7.8986560000	5.1945030000

C	1.7223480000	6.5062450000	4.5016610000
O	0.2579430000	9.3791600000	4.1067900000
C	-0.4921830000	9.7450060000	2.9834060000
H	-0.0850680000	10.6487350000	2.5215660000
H	-0.4831870000	8.9300960000	2.2523890000
O	-1.8169760000	10.0659170000	3.3476530000
H	-2.2353820000	9.2516130000	3.6544300000

$TS_{10,8}$

Mn	-0.3178860000	-0.4378630000	-0.2376200000
P	-0.0849800000	-2.7520930000	-0.6806070000
P	0.1637010000	1.6105350000	0.6635440000
O	-3.1820790000	-0.1279610000	-0.6705550000
O	-1.2462520000	-1.1246490000	2.4302570000
N	2.6321000000	-0.7794610000	1.3899520000
H	2.3592190000	-0.5210910000	0.4495590000
C	1.6702880000	-3.2974650000	-0.6824770000
C	2.1682910000	-3.9872070000	-1.7907680000
H	1.5134240000	-4.2098600000	-2.6220000000
C	3.4923210000	-4.3866240000	-1.8635810000
H	3.8439130000	-4.9191370000	-2.7376490000
C	4.3519920000	-4.1064010000	-0.8148070000
H	5.3865160000	-4.4221370000	-0.8518730000
C	3.8764750000	-3.4114940000	0.2833870000
H	4.5436300000	-3.1824320000	1.1063160000
C	2.5530720000	-2.9837760000	0.3727960000
C	2.1828870000	-2.1609270000	1.5794360000
H	1.1067890000	-2.2242500000	1.7799420000
H	2.6694540000	-2.5835000000	2.4632600000
C	2.0499420000	0.2049410000	2.3053120000
H	2.7486820000	0.4100040000	3.1216230000
H	1.1463430000	-0.1804540000	2.7908790000
C	1.7580430000	1.5195560000	1.5850400000
H	2.5435850000	1.7095520000	0.8504600000
H	1.7895660000	2.3560450000	2.2845340000
C	-2.0373880000	-0.2604100000	-0.4821650000
C	-0.8166020000	-0.8698340000	1.3721240000
C	-1.0452790000	2.2371350000	1.8717970000
H	0.1200250000	1.8500680000	3.6431170000
C	-1.8169570000	2.6087970000	4.1283030000
H	-1.6310160000	2.5755410000	5.1941380000
C	-3.0372040000	3.0573890000	3.6480050000
H	-3.8083130000	3.3770640000	4.3368400000
C	-3.2676130000	3.0935750000	2.2787460000

H	-4.2180660000	3.4424550000	1.8959670000
C	-2.2821870000	2.6809780000	1.3981590000
H	-2.4727180000	2.7051800000	0.3304530000
C	-0.8248340000	2.2006000000	3.2471470000
C	0.3709430000	3.0090100000	-0.4812380000
H	1.7635560000	4.1194150000	0.7309480000
C	1.3021410000	5.1494320000	-1.0862020000
H	1.9502380000	5.9853110000	-0.8560520000
C	0.5756730000	5.1369090000	-2.2692300000
H	0.6590650000	5.9626480000	-2.9640160000
C	-0.2550740000	4.0651050000	-2.5605270000
H	-0.8228180000	4.0506580000	-3.4818680000
C	-0.3540610000	3.0047940000	-1.6724110000
H	-0.9966790000	2.1602960000	-1.9004750000
C	1.1990110000	4.0944090000	-0.1930990000
C	-0.8857560000	-3.7849110000	0.6016060000
H	0.8763970000	-4.7479360000	1.3741110000
C	-0.8814450000	-5.3623290000	2.4269500000
H	-0.3319760000	-6.0211140000	3.0870740000
C	-2.2570700000	-5.2468540000	2.5450900000
H	-2.7873610000	-5.8115090000	3.3008950000
C	-2.9535980000	-4.4052200000	1.6866560000
H	-4.0287680000	-4.3116310000	1.7693960000
C	-2.2735180000	-3.6791330000	0.7250370000
H	-2.8236920000	-3.0176360000	0.0635510000
C	-0.1976350000	-4.6379910000	1.4586020000
C	-0.8697260000	-3.4782600000	-2.1615570000
H	-1.4990000000	-1.5771060000	-2.9306940000
C	-2.1942880000	-3.1918790000	-4.1587960000
H	-2.6951450000	-2.5370940000	-4.8599780000
C	-2.2311860000	-4.5655670000	-4.3381980000
H	-2.7594130000	-4.9884690000	-5.1829890000
C	-1.5967270000	-5.4011470000	-3.4271780000
H	-1.6297330000	-6.4748460000	-3.5596640000
C	-0.9267330000	-4.8626160000	-2.3424470000
H	-0.4445010000	-5.5193780000	-1.6269380000
C	-1.5180000000	-2.6509400000	-3.0741000000
O	1.5256450000	-0.1135980000	-1.1262180000
C	0.9829390000	-0.0232560000	-2.3618870000
H	-0.1453260000	-0.0148320000	-2.3495300000
H	1.2578760000	0.9196430000	-2.8673200000
O	1.3056750000	-1.1216770000	-3.2112480000
H	2.2360560000	-1.3200530000	-3.0370470000

$TS_{7,11}$

Mn	2.1568230000	8.3049310000	4.6190590000
P	1.1832590000	8.4981940000	6.6709520000
P	2.9916250000	8.4021440000	2.4692100000
O	4.6323780000	7.3809380000	5.8201350000
O	1.3447900000	5.5020060000	4.1791770000
N	2.4180430000	10.2582710000	4.8202970000
H	-0.5370030000	10.1127950000	4.1255170000
C	1.2498030000	10.2914590000	6.9636730000
H	0.3222660000	10.6964690000	6.5514430000
H	1.2913600000	10.5640760000	8.0180250000
C	2.4260130000	10.8160240000	6.1693290000
H	2.3722000000	11.9119390000	6.1258500000
H	3.3664690000	10.5913360000	6.7023440000
C	3.3838390000	10.9966450000	4.0035830000
H	4.3862250000	10.5355280000	4.0507880000
H	3.5173440000	12.0045870000	4.4111890000
C	2.9741480000	11.1542250000	2.5668570000
C	2.7924530000	12.4276390000	2.0364190000
H	2.9554220000	13.2870530000	2.6764360000
C	2.4039550000	12.6108310000	0.7176080000
H	2.2628780000	13.6120890000	0.3309770000
C	2.1907140000	11.5113680000	-0.0989840000
H	1.8868770000	11.6440020000	-1.1292620000
C	2.3720300000	10.2343150000	0.4091900000
H	2.2100020000	9.3770260000	-0.2327570000
C	2.7669730000	10.0457710000	1.7325070000
C	2.1103650000	7.6912440000	8.0127420000
C	2.0883140000	6.2963920000	8.0868480000
H	1.4694030000	5.7288670000	7.3995910000
C	2.8568300000	5.6286500000	9.0248860000
H	2.8249010000	4.5477730000	9.0740870000
C	3.6712760000	6.3431050000	9.8943880000
H	4.2763260000	5.8211670000	10.6243160000
C	3.7072730000	7.7266890000	9.8214140000
H	4.3409160000	8.2901840000	10.4943490000
C	2.9314750000	8.3999540000	8.8868930000
H	2.9747180000	9.4808080000	8.8434570000
C	-0.5406310000	8.0105100000	7.0058380000
C	-1.3641880000	8.8017570000	7.8076820000
H	-0.9822590000	9.7162550000	8.2441200000
C	-2.6763570000	8.4271280000	8.0534590000
H	-3.3050470000	9.0536730000	8.6731130000
C	-3.1812970000	7.2545560000	7.5096500000
H	-4.2066610000	6.9654410000	7.7006040000
C	-2.3688900000	6.4569430000	6.7176070000

H	-2.7564060000	5.5423510000	6.2874460000
C	-1.0585800000	6.8340310000	6.4632450000
H	-0.4385550000	6.2120200000	5.8305570000
C	2.2609730000	7.2780200000	1.2326180000
C	0.8842220000	7.0533360000	1.2767040000
H	0.2925610000	7.5130840000	2.0602420000
C	0.2699150000	6.2482470000	0.3315510000
H	-0.7990090000	6.0839030000	0.3787910000
C	1.0250880000	5.6491450000	-0.6674900000
H	0.5473600000	5.0134920000	-1.4018190000
C	2.3936490000	5.8665040000	-0.7209190000
H	2.9873600000	5.4047290000	-1.4993410000
C	3.0088670000	6.6802690000	0.2196300000
H	4.0765760000	6.8516490000	0.1599760000
C	4.7776470000	8.0742270000	2.3317530000
C	5.2365250000	6.8156620000	2.7297570000
H	4.5272490000	6.0739350000	3.0837170000
C	6.5847630000	6.5092060000	2.6748190000
H	6.9258540000	5.5279460000	2.9788090000
C	7.4973020000	7.4613060000	2.2368140000
H	8.5526710000	7.2241390000	2.2004560000
C	7.0523930000	8.7147980000	1.8476950000
H	7.7583370000	9.4600110000	1.5042590000
C	5.6982080000	9.0203320000	1.8887430000
H	5.3596490000	9.9990810000	1.5706290000
C	3.6316010000	7.7212150000	5.3157350000
C	1.6078330000	6.6330610000	4.3428830000
O	-0.5982460000	9.2268550000	3.7233700000
C	-1.7962860000	8.7082010000	3.9797280000
H	-1.8609360000	7.6920370000	3.5663060000
O	-2.6873260000	9.2719920000	4.5644780000

$TS_{11,8}$

Mn	-0.2889500000	-0.4910660000	-0.1577930000
P	-0.0958990000	-2.7792290000	-0.6196790000
P	0.1947140000	1.6100210000	0.6739460000
O	-3.1256030000	-0.1418910000	-0.7016730000
O	-1.2916560000	-1.1296840000	2.4972860000
N	2.6291840000	-0.7722320000	1.4027150000
H	2.3506640000	-0.5190040000	0.4624710000
C	1.6688010000	-3.2863790000	-0.6483120000
C	2.1592920000	-3.9509610000	-1.7752300000
H	1.4953720000	-4.1763280000	-2.5982620000
C	3.4871110000	-4.3339000000	-1.8698480000

H	3.8337440000	-4.8481950000	-2.7565770000
C	4.3554590000	-4.0626370000	-0.8267310000
H	5.3926710000	-4.3666110000	-0.8811060000
C	3.8866580000	-3.3899640000	0.2884910000
H	4.5619860000	-3.1649860000	1.1057180000
C	2.5611140000	-2.9769420000	0.4000910000
C	2.2025560000	-2.1598130000	1.6127390000
H	1.1329650000	-2.2403080000	1.8421200000
H	2.7204580000	-2.5657770000	2.4857040000
C	2.0699810000	0.2183280000	2.3254740000
H	2.7839250000	0.4167090000	3.1295280000
H	1.1689450000	-0.1565830000	2.8246940000
C	1.7856440000	1.5309940000	1.6010980000
H	2.5734480000	1.7084830000	0.8650120000
H	1.8208200000	2.3740820000	2.2924550000
C	-1.9916520000	-0.2832850000	-0.4818710000
C	-0.8330010000	-0.8892180000	1.4535420000
C	-1.0293290000	2.2380530000	1.8637120000
H	0.1418990000	1.8831620000	3.6386240000
C	-1.8009480000	2.6306420000	4.1155240000
H	-1.6132710000	2.6125490000	5.1813280000
C	-3.0255740000	3.0620080000	3.6307960000
H	-3.7988890000	3.3833470000	4.3163010000
C	-3.2575250000	3.0790140000	2.2617400000
H	-4.2112040000	3.4145010000	1.8752850000
C	-2.2697310000	2.6645250000	1.3845750000
H	-2.4638230000	2.6752170000	0.3178840000
C	-0.8060520000	2.2202930000	3.2389920000
C	0.4054910000	2.9737920000	-0.5113760000
H	1.9902290000	3.9798640000	0.5470730000
C	1.4653690000	5.0094090000	-1.2516890000
H	2.2034490000	5.7863760000	-1.0998800000
C	0.6424040000	5.0393920000	-2.3686150000
H	0.7397260000	5.8391030000	-3.0913710000
C	-0.3020260000	4.0414550000	-2.5590880000
H	-0.9455050000	4.0583980000	-3.4290780000
C	-0.4161390000	3.0114690000	-1.6390240000
H	-1.1461990000	2.2247750000	-1.7969840000
C	1.3468240000	3.9863030000	-0.3236400000
C	-0.8887270000	-3.8386940000	0.6354540000
H	0.8851440000	-4.8012760000	1.3818380000
C	-0.8644530000	-5.4428350000	2.4345750000
H	-0.3088250000	-6.1089010000	3.0819300000
C	-2.2402270000	-5.3363100000	2.5603070000
H	-2.7636280000	-5.9159340000	3.3095110000
C	-2.9464630000	-4.4840110000	1.7205460000

H	-4.0214070000	-4.3970920000	1.8117350000
C	-2.2759090000	-3.7380280000	0.7676190000
H	-2.8312440000	-3.0659900000	0.1213150000
C	-0.1891410000	-4.7002350000	1.4745800000
C	-0.8387150000	-3.4359580000	-2.1484740000
H	-1.2294730000	-1.4992030000	-2.9942700000
C	-1.9051070000	-3.0700030000	-4.2818240000
H	-2.2705640000	-2.3858530000	-5.0363850000
C	-2.0360670000	-4.4379870000	-4.4576910000
H	-2.5021180000	-4.8275820000	-5.3534650000
C	-1.5762560000	-5.3102100000	-3.4787640000
H	-1.6816460000	-6.3793260000	-3.6098860000
C	-0.9847620000	-4.8141220000	-2.3304990000
H	-0.6294190000	-5.4986310000	-1.5684890000
C	-1.3114690000	-2.5701030000	-3.1308520000
O	1.6775560000	-0.2320000000	-1.3505780000
C	0.9544900000	0.0766120000	-2.3367930000
H	-0.1650530000	0.1329170000	-2.1104720000
O	1.2727240000	0.3231730000	-3.4969880000

$TS_{11,12}$

Mn	1.8322100000	8.2576900000	4.5786110000
P	0.9196360000	8.3718930000	6.6760430000
P	2.6839920000	8.4543300000	2.4142470000
O	4.4636470000	7.8690530000	5.7541140000
O	1.6829610000	5.3286310000	4.4058630000
N	1.8968160000	10.4017940000	4.8110790000
H	0.9470740000	10.6435100000	4.5134770000
C	0.9396380000	10.1623080000	7.0502890000
H	-0.0336280000	10.5538850000	6.7493280000
H	1.0602220000	10.3693710000	8.1126250000
C	2.0332380000	10.8055140000	6.2273200000
H	1.9832100000	11.8950790000	6.3081410000
H	3.0247620000	10.5012940000	6.5706260000
C	2.8530450000	11.1388980000	3.9514340000
H	3.8290670000	10.6527340000	4.0418400000
H	2.9713500000	12.1560510000	4.3331810000
C	2.3901190000	11.2164580000	2.5269620000
C	2.0257790000	12.4548720000	2.0096210000
H	2.0951050000	13.3262690000	2.6495750000
C	1.5785190000	12.5865090000	0.7041980000
H	1.2958600000	13.5603330000	0.3262810000
C	1.4902700000	11.4681950000	-0.1082600000
H	1.1409260000	11.5578730000	-1.1285190000

C	1.8465920000	10.2251190000	0.3899290000
H	1.7625360000	9.3545450000	-0.2478850000
C	2.2997190000	10.0834030000	1.7001420000
C	1.9079060000	7.5568110000	7.9623360000
C	1.8520080000	6.1641850000	8.0530540000
H	1.1678010000	5.6103350000	7.4189010000
C	2.6632940000	5.4862000000	8.9469220000
H	2.6070330000	4.4072580000	9.0115230000
C	3.5470180000	6.1887490000	9.7561040000
H	4.1832980000	5.6587180000	10.4528390000
C	3.6118350000	7.5705900000	9.6682830000
H	4.2986110000	8.1237670000	10.2959160000
C	2.7987360000	8.2541820000	8.7748900000
H	2.8695470000	9.3329130000	8.7140100000
C	-0.7696230000	7.7953540000	7.0032390000
C	-1.3990250000	6.9346370000	6.1070100000
H	-0.8831440000	6.6248720000	5.2052950000
C	-2.6820350000	6.4727620000	6.3630490000
H	-3.1625340000	5.8037350000	5.6610830000
C	-3.3443880000	6.8690920000	7.5146610000
H	-4.3458840000	6.5103980000	7.7141820000
C	-2.7228360000	7.7258660000	8.4142170000
H	-3.2379920000	8.0346000000	9.3146630000
C	-1.4405070000	8.1850460000	8.1638150000
H	-0.9567850000	8.8464980000	8.8733650000
C	2.1221480000	7.2513530000	1.1689530000
C	0.8841210000	6.6282010000	1.3148210000
H	0.2735970000	6.8386890000	2.1849520000
C	0.4296320000	5.7339840000	0.3574220000
H	-0.5326460000	5.2554050000	0.4845380000
C	1.2101510000	5.4489830000	-0.7530200000
H	0.8579230000	4.7461420000	-1.4969310000
C	2.4456530000	6.0625010000	-0.9066700000
H	3.0582900000	5.8428890000	-1.7714190000
C	2.8996520000	6.9591970000	0.0467300000
H	3.8626620000	7.4390850000	-0.0822090000
C	4.4959740000	8.3055920000	2.3186560000
C	5.0645420000	7.0871360000	2.6976430000
H	4.4247480000	6.2740360000	3.0259770000
C	6.4365540000	6.9124690000	2.6573210000
H	6.8658560000	5.9624770000	2.9481470000
C	7.2593950000	7.9554020000	2.2498740000
H	8.3327360000	7.8196230000	2.2230880000
C	6.7028670000	9.1690490000	1.8786480000
H	7.3386120000	9.9843090000	1.5583040000
C	5.3257750000	9.3444510000	1.9077290000

H	4.8995000000	10.2934330000	1.6053700000
C	3.4148000000	8.0251230000	5.2733840000
C	1.6991400000	6.4910260000	4.4526180000
O	-0.8770320000	9.9519090000	4.1135260000
C	-0.9848610000	9.1709740000	3.1341180000
H	-1.0184100000	8.0795920000	3.3864590000
O	-1.0662770000	9.4714740000	1.9253200000

$TS_{12,8}$

Mn	2.0370410000	8.2555800000	4.6197830000
P	1.0373960000	8.3881580000	6.6294410000
P	2.7546090000	8.4728720000	2.4714520000
O	4.7148960000	7.9522390000	5.7958410000
O	1.8131860000	5.3377100000	4.5150760000
N	2.0670660000	10.4385950000	4.8472910000
C	1.1402140000	10.1692040000	7.0825710000
H	0.1766370000	10.6141590000	6.8254160000
H	1.2924860000	10.3180150000	8.1507310000
C	2.2356970000	10.8161550000	6.2643170000
H	2.2024590000	11.9051590000	6.3696960000
H	3.2264310000	10.4890410000	6.5864640000
C	3.0058980000	11.1632520000	3.9592830000
H	3.9727870000	10.6531130000	3.9998710000
H	3.1617350000	12.1784740000	4.3341300000
C	2.4472640000	11.2377800000	2.5700920000
C	2.0257150000	12.4687870000	2.0806110000
H	2.1696190000	13.3497230000	2.6949220000
C	1.4186230000	12.5796250000	0.8390210000
H	1.0938330000	13.5476330000	0.4804460000
C	1.2198960000	11.4451320000	0.0701210000
H	0.7389570000	11.5160920000	-0.8967980000
C	1.6431080000	10.2101480000	0.5389500000
H	1.4837870000	9.3308380000	-0.0720090000
C	2.2657280000	10.0894150000	1.7796280000
C	1.8424340000	7.4852790000	7.9888260000
C	1.4850690000	6.1612520000	8.2481900000
H	0.6692490000	5.7054660000	7.6976450000
C	2.1599760000	5.4253870000	9.2095830000
H	1.8677720000	4.4013340000	9.4038810000
C	3.2032270000	5.9997840000	9.9223890000
H	3.7280500000	5.4257100000	10.6749210000
C	3.5701240000	7.3130410000	9.6665740000
H	4.3830020000	7.7677400000	10.2182120000
C	2.8981470000	8.0507980000	8.7034390000

H	3.2030220000	9.0723210000	8.5083810000
C	-0.7147410000	7.9456030000	6.8500060000
C	-1.3692500000	7.1676510000	5.8981700000
H	-0.8321260000	6.8369460000	5.0163830000
C	-2.7023340000	6.8211260000	6.0694250000
H	-3.2006000000	6.2164110000	5.3225370000
C	-3.3926320000	7.2517220000	7.1920000000
H	-4.4338430000	6.9866380000	7.3233340000
C	-2.7466250000	8.0230470000	8.1498590000
H	-3.2823540000	8.3587130000	9.0285290000
C	-1.4142950000	8.3635780000	7.9836590000
H	-0.9132380000	8.9589080000	8.7386340000
C	2.1564330000	7.2629310000	1.2418550000
C	0.9640480000	6.5754240000	1.4604270000
H	0.4094230000	6.7448500000	2.3747670000
C	0.4839590000	5.6793930000	0.5166400000
H	-0.4431520000	5.1520080000	0.7013100000
C	1.1918970000	5.4560050000	-0.6547880000
H	0.8194350000	4.7528360000	-1.3885890000
C	2.3816260000	6.1339100000	-0.8822110000
H	2.9384900000	5.9641780000	-1.7947530000
C	2.8606580000	7.0315990000	0.0581410000
H	3.7871390000	7.5612670000	-0.1307380000
C	4.5606190000	8.3977230000	2.2102870000
C	5.2149980000	7.2304080000	2.6118660000
H	4.6423960000	6.4188900000	3.0507680000
C	6.5839400000	7.1010550000	2.4555340000
H	7.0768730000	6.1884370000	2.7652110000
C	7.3233350000	8.1430200000	1.9088250000
H	8.3948150000	8.0446180000	1.7919980000
C	6.6843550000	9.3080430000	1.5150730000
H	7.2543550000	10.1228590000	1.0873300000
C	5.3086230000	9.4344110000	1.6591830000
H	4.8186310000	10.3451150000	1.3359460000
C	3.6518140000	8.0913320000	5.3313520000
C	1.8866370000	6.5055890000	4.5329720000
H	0.5000710000	8.5288250000	3.8973770000
C	-0.8552640000	9.3848530000	3.3032390000
O	-1.3782780000	8.5731590000	2.6241980000
O	-0.7553160000	10.4536650000	3.8025430000
H	1.1240200000	10.7167170000	4.5773580000

H_2

H	-5.8396930000	-0.7819170000	0.0000000000

H	-5.0990220000	-0.8555790000	0.0000000000

CO_2

C	-1.9599830000	1.8399080000	-1.1392880000
O	-1.0077800000	1.8641200000	-1.7997490000
O	-2.9121860000	1.8156960000	-0.4788270000

H_2O

O	-8.2273610000	-1.6391570000	6.2520650000
H	-7.2664980000	-1.5889830000	6.2467410000
H	-8.5000790000	-0.7215740000	6.1546980000

CH_2O

O	-7.5819630000	-3.0691900000	-0.0036400000
C	-8.7851380000	-3.0691900000	-0.0036400000
H	-9.3755840000	-2.5974690000	0.8054590000
H	-9.3755840000	-3.5409110000	-0.8127390000

$CH_2(OH)_2$

O	-8.4420360000	-1.6741980000	6.0551800000
C	-7.0903640000	-1.5485590000	6.4285300000
H	-8.8452480000	-0.8000780000	6.1217260000
H	-6.7182780000	-2.5683930000	6.5446080000
H	-6.9877710000	-1.0079040000	7.3715470000
O	-6.3269770000	-0.8286130000	5.4899850000
H	-6.2526040000	-1.3725520000	4.6967210000

HCOOH

C	-2.2575870000	1.8757180000	-1.4049120000
O	-2.6970630000	1.8583660000	-0.2819790000
H	-2.6493410000	2.4990400000	-2.2193890000
O	-1.2324930000	1.1478570000	-1.8426890000
H	-0.9006070000	0.6031350000	-1.1085260000

MeOH

C	-5.9166700000	-0.7875860000	-0.0044060000
O	-4.4984890000	-0.7983990000	-0.0166570000
H	-6.3327610000	-1.7398220000	0.3353800000
H	-6.3356020000	-0.5573210000	-0.9877310000
H	-6.2306320000	-0.0110400000	0.6903470000
H	-4.2222350000	-1.4906830000	-0.6256000000

9. References

(1) Chakraborty, S.; Gellrich, U.; Diskin-Posner, Y.; Leitus, G.; Avram, L.; Milstein, D. Manganese-Catalyzed N-Formylation of Amines by Methanol Liberating H_2: A Catalytic and Mechanistic Study. *Angew. Chem. Int. Ed.* **2017,** *56*, 4229-4233.

(2) Das, U. K.; Ben-David, Y.; Leitus, G.; Diskin-Posner, Y.; Milstein, D. Dehydrogenative Cross-Coupling of Primary Alcohols to Form Cross-Esters Catalyzed by a Manganese Pincer Complex. *ACS Catal.* **2018,** *9*, 479-484.

(3) Espinosa-Jalapa, N. A.; Kumar, A.; Leitus, G.; Diskin-Posner, Y.; Milstein, D. Synthesis of Cyclic Imides by Acceptorless Dehydrogenative Coupling of Diols and Amines Catalyzed by a Manganese Pincer Complex. *J. Am. Chem. Soc.* **2017,** *139*, 11722-11725.

(4) Nerush, A.; Vogt, M.; Gellrich, U.; Leitus, G.; Ben-David, Y.; Milstein, D. Template Catalysis by Metal-Ligand Cooperation. C-C Bond Formation Via Conjugate Addition of Non-Activated Nitriles under Mild, Base-Free Conditions Catalyzed by a Manganese Pincer Complex. *J. Am. Chem. Soc.* **2016,** *138*, 6985-6997.

(5) Sonnenberg, J. F.; Lough, A. J.; Morris, R. H. Synthesis of Iron P-N-P′ and P-NH-P′ Asymmetric Hydrogenation Catalysts. *Organometallics* **2014,** *33*, 6452-6465.

(6) Rigaku Corporation.

(7) Sheldrick, G. M. SHELXT - Integrated Space-Group and Crystal-Structure Determination. *Acta Cryst.* **2015,** *A71*, 3-8.

(8) Sheldrick, G. M. A Short History of SHELXT. *Acta Cryst.* **2008,** *A64*, 112-122.

(9) Dolomanov, O. V.; Bourhis, L. J.; Gildea, R. J.; Howard, J. A. K.; Puschmann, H. OLEX 2: A Complete Structure Solution, Refinement and Analysis Program. *J. Appl. Cryst.* **2009,** *42*, 339-341.

(10) Polidano, K.; Allen, B. D. W.; Williams, J. M. J.; Morrill, L. C. Iron-Catalyzed Methylation Using the Borrowing Hydrogen Approach. *ACS Catal.* **2018,** *8*, 6440-6445.

(11) Latham, D. E.; Polidano, K.; Williams, J. M. J.; Morrill, L. C. One-Pot Conversion of Allylic Alcohols to A-Methyl Ketones via Iron-Catalyzed Isomerization-Methylation. *Org. Lett.* **2019,** *21*, 7914-7918.

(12) Paul, B.; Shee, S.; Panja, D.; Chakrabarti, K.; Kundu, S. Direct Synthesis of N,N-Dimethylated and β-Methyl N,N-Dimethylated Amines from Nitriles Using Methanol: Experimental and Computational Studies. *ACS Catal.* **2018,** *8*, 2890-2896.

(13) Cui, X.; Dai, X.; Deng, Y.; Shi, F. Development of a General Non-Noble Metal Catalyst for the Benign Amination of Alcohols with Amines and Ammonia. *Chem. Eur. J.* **2013,** *19*, 3665-3675.

(14) Liang, R.; Li, S.; Wang, R.; Lu, L.; Li, F. *N*-Methylation of Amines with Methanol Catalyzed by a Cp*Ir Complex Bearing a Functional 2,2'-Bibenzimidazole Ligand. *Org. Lett.* **2017,** *19*, 5790-5793.
(15) Mbofana, C. T.; Chong, E.; Lawniczak, J.; Sanford, M. S. Iron-Catalyzed Oxyfunctionalization of Aliphatic Amines at Remote Benzylic C-H Sites. *Org. Lett.* **2016,** *18*, 4258-4261.
(16) Wang, M.-Y.; Wang, N.; Liu, X.-F.; Qiao, C.; He, L.-N. Tungstate Catalysis: Pressure-Switched 2- and 6-Electron Reductive Functionalization of CO_2 with Amines and Phenylsilane. *Green Chem.* **2018,** *20*, 1564-1570.
(17) Frisch, M. J.; Trucks, G. W.; Schlegel, H. B.; Scuseria, G. E.; Robb, M. A.; Cheeseman, J. R.; Scalmani, G.; Barone, V.; Petersson, G. A.; Nakatsuji, H.; Li, X.; Caricato, M.; Marenich, A. V.; Bloino, J.; Janesko, B. G.; Gomperts, R.; Mennucci, B.; Hratchian, H. P.; Ortiz, J. V.; Izmaylov, A. F.; Sonnenberg, J. L.; Williams-Young, D.; Ding, F.; Lipparini, F.; Egidi, F.; Goings, J.; Peng, B.; Petrone, A.; Henderson, T.; Ranasinghe, D.; Zakrzewski, V. G.; Gao, J.; Rega, N.; Zheng, G.; Liang, W.; Hada, M.; Ehara, M.; Toyota, K.; Fukuda, R.; Hasegawa, J.; Ishida, M.; Nakajima, T.; Honda, Y.; Kitao, O.; Nakai, H.; Vreven, T.; Throssell, K.; Montgomery, J. A., Jr.; Peralta, J. E.; Ogliaro, F.; Bearpark, M. J.; Heyd, J. J.; Brothers, E. N.; Kudin, K. N.; Staroverov, V. N.; Keith, T. A.; Kobayashi, R.; Normand, J.; Raghavachari, K.; Rendell, A. P.; Burant, J. C.; Iyengar, S. S.; Tomasi, J.; Cossi, M.; Millam, J. M.; Klene, M.; Adamo, C.; Cammi, R.; Ochterski, J. W.; Martin, R. L.; Morokuma, K.; Farkas, O.; Foresman, J. B.; Fox, D. J. *Gaussian 16, Revision C.01;* Gaussian, Inc., Wallingford CT, 2016.
(18) Zhao, Y.; Truhlar, D. G. A New Local Density Functional for Main-Group Thermochemistry, Transition Metal Bonding, Thermochemical Kinetics, and Noncovalent Interactions. *J. Chem. Phys.* **2006,** *125*, 194101.
(19) Weigend, F.; Ahlrichs, R. Balanced Basis Sets of Split Valence, Triple Zeta Valence and Quadruple Zeta Valence Quality for H to Rn: Design and Assessment of Accuracy. *Phys. Chem. Chem. Phys.* **2005,** *7*, 3297-3305.
(20) Weigend, F. Accurate Coulomb-Fitting Basis Sets for H to Rn. *Phys. Chem. Chem. Phys.* **2006,** *8*, 1057-1065.
(21) Grimme, S.; Antony, J.; Ehrlich, S.; Krieg, H. A Consistent and Accurate *ab initio* Parametrization of Density Functional Dispersion Correction (DFT-D) for the 94 Elements H-Pu. *J. Chem. Phys.* **2010,** *132*, 154104.
(22) Neese, F. Software Update: The ORCA Program System—Version 5.0. *WIREs Comput. Mol. Sci.* **2022,** *12*, e1606.
(23) Mardirossian, N.; Head-Gordon, M. ωB97X-V: A 10-Parameter, Range-Separated Hybrid, Generalized Gradient Approximation Density Functional with Nonlocal Correlation, Designed by a Survival-of-the-Fittest Strategy. *Phys. Chem. Chem. Phys.* **2014,** *16*, 9904-9924.
(24) Vydrov, O. A.; Van Voorhis, T. Nonlocal van der Waals Density Functional: The Simpler the Better. *J. Chem. Phys.* **2010,** *133*, 244103.
(25) Hujo, W.; Grimme, S. Performance of the van der Waals Density Functional VV10 and (hybrid) GGA Variants for Thermochemistry and Noncovalent Interactions. *J. Chem. Theory Comput.* **2011,** *7*, 3866-3871.
(26) Hellweg, A.; Hättig, C.; Höfener, S.; Klopper, W. Optimized Accurate Auxiliary Basis Sets for RI-MP2 and RI-CC2 Calculations for the Atoms Rb to Rn. *Theor. Chem. Acc.* **2007,** *117*, 587-597.

(27) Iron, M. A.; Janes, T. Evaluating Transition Metal Barrier Heights with the Latest Density Functional Theory Exchange-Correlation Functionals: The MOBH35 Benchmark Database. *J. Phys. Chem. A* **2019,** *123*, 3761-3781.
(28) Marenich, A. V.; Cramer, C. J.; Truhlar, D. G. Universal Solvation Model Based on Solute Electron Density and on a Continuum Model of the Solvent Defined by the Bulk Dielectric Constant and Atomic Surface Tensions. *J. Phys. Chem. B* **2009,** *113*, 6378-6396.
(29) Cramer, C. J. *Essentials of Computational Chemistry: Theories and Models. 2nd Ed.;* John Wiley & Sons Ltd: West Sussex, England, 2004.
(30) Sparta, M.; Riplinger, C.; Neese, F. Mechanism of Olefin Asymmetric Hydrogenation Catalyzed by Iridium Phosphino-Oxazoline: A Pair Natural Orbital Coupled Cluster Study. *J. Chem. Theory Comput.* **2014,** *10*, 1099-1108.
(31) Hopmann, K. H. How Accurate Is Dft for Iridium-Mediated Chemistry? *Organometallics* **2016,** *35*, 3795-3807.
(32) Gusev, D. G. Revised Mechanisms of the Catalytic Alcohol Dehydrogenation and Ester Reduction with the Milstein PNN Complex of Ruthenium. *Organometallics* **2020,** *39*, 258-270.
